# Paving the way towards effective plant-based inhibitors of hyaluronidase and tyrosinase: a critical review on a structure–activity relationship

**DOI:** 10.1080/14756366.2022.2061966

**Published:** 2022-04-26

**Authors:** Jakub Gębalski, Filip Graczyk, Daniel Załuski

**Affiliations:** Department of Pharmaceutical Botany and Pharmacognosy, Ludwik Rydygier Collegium Medicum, Nicolaus Copernicus University, Bydgoszcz, Poland

**Keywords:** Hyaluronidase, tyrosinase plant-based inhibitors, structure–activity relationship, polyphenols

## Abstract

Human has used plants to treat many civilisation diseases for thousands of years. Examples include reserpine (hypertension therapy), digoxin (myocardial diseases), vinblastine and vincristine (cancers), and opioids (palliative treatment). Plants are a rich source of natural metabolites with multiple biological activities, and the use of modern approaches and tools allowed finally for more effective bioprospecting. The new phytochemicals are hyaluronidase (Hyal) inhibitors, which could serve as anti-cancer drugs, male contraceptives, and an antidote against venoms. In turn, tyrosinase inhibitors can be used in cosmetics/pharmaceuticals as whitening agents and to treat skin pigmentation disorders. However, the activity of these inhibitors is *stricte* dependent on their structure and the presence of the chemical groups, e.g. carbonyl or hydroxyl. This review aims to provide comprehensive and in-depth evidence related to the anti-tyrosinase and anti-Hyal activity of phytochemicals as well as confirming their efficiency and future perspectives.

## Graphical Abstract

**Figure:**
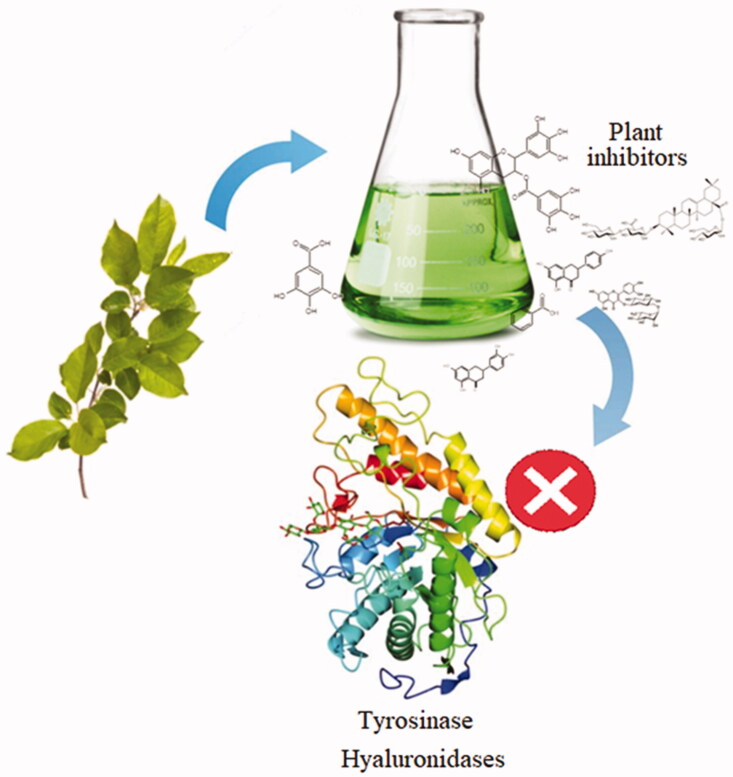

## 1. Introduction

Before the advancement of modern science, herbal medicines were widely used in ethnomedicine. Modern medicine primarily uses plant active ingredients that can be divided into primary and secondary metabolites. Secondary metabolites are defined as substances that are not directly necessary for the organism’s growth and development. It is believed that they play an important role in adapting plants to changing external conditions. Secondary metabolites are formed in various metabolic pathways, including amino acids and sugars intermediates, therefore, plants can synthesise many structurally diverse metabolites. It is estimated that plants produce at least 250,000 natural products, many of which have not yet been identified and characterised in structure and biological activity^1^. The enormous diversity in the plant world (250,000–300,000 species) has provided many substances currently used in treatment^2^^,^^3^. The isolation of morphine and codeine from *Papaver somniferum* L. ensured an effective pain treatment^4^. Obtaining digoxin from *Digitalis purpurea* L. allowed for an effective treatment of atrial fibrillation and heart failure^5^. Salicylic acid, which is found, among others, in *Salix alba* L., after esterification forms acetylsalicylic acid, used in pain and thrombotic diseases^6^. The isolated artimizin from *Artemisia annua* L. is used in the treatment of a resistant form of malaria^7^. Other compounds used in the treatment are capsaicin from *Capsicum annuum* L.^8^, quinine from *Cinchona* L.^9^, and inulin^10^. Additionally, some compounds, after structure modification, have contributed to a new group of drugs. A good example is a phlorizin, which has served as the host structure for SGLT2 sodium-glucose co-transporter inhibitors, e.g. dapagliflozin^11^. The development of new branches of science, such as genetics, biotechnology, and molecular biology, allowed for a better understanding of diseases’ pathophysiology^12^^,^^13^. Hyals and tyrosinases are an underestimated group of enzymes commonly found in the world of organisms. Hyal, due to its participation in many biological processes, such as fertilisation, diffusion of toxins and microorganisms, inflammatory and allergic reactions, and cancer development, is perceived as a potential therapeutic target^14^. Besides, the inhibitors of tyrosinases and Hyals can be used in cosmetology to develop cosmetics or drugs used in dermatological diseases, such as eczema, acne, discolouration, and photoaging^15–17^.

This study aims to systematise knowledge about the Hyal and tyrosinase inhibitors. We have hypothesised that there are the plant-based compounds and their chemically modified derivatives which inhibit the Hyal and tyrosinase.

## 2. Methods

To confirm our hypothesis we have searched for the different available databases (ScienceDirect, PubMed, Scopus, Web of Science, Google Scholar, and ClinicalTrials) in the regard to the relationship between the structure and activity of inhibitors, their action’s mechanism, and the prospects for their use in treatment. Search terms included “natural substances”, “plant substances”, “polyphenols”, “phenolic acids”, “chalcones”, “stilbenes”, “lignans”, “terpenes”, “alkaloids”, “glycosides”, “tyrosinase”, “tyrosinase inhibitors”, “hyaluronidase” (Hyal), “hyaluronidase inhibitors”, and “bacterial lyase”. The search equation was defined according to the formula [tyrosinase inhibitor OR tyrosinase OR Hyal inhibitor OR Hyal OR bacterial lyase inhibitor OR bacterial lyase] AND [natural substances OR plant substances OR polyphenols OR phenolic acids OR chalcones OR stilbenes OR lignans OR terpenes OR alkaloids OR glycosides]. Publications were searched from January 1990 to December 2021.

## 3. Types of inhibition

Inhibition is the process that slows down or completely stops a chemical reaction by a substance called an inhibitor. Depending on the way of binding of the inhibitor with the enzyme, one can distinguish reversible and irreversible inhibition (Table 1).

**Table 1.** Effect of the inhibitor on *K*_m_ and *V*_max_.

Types of inhibitor	*K*_m_	*V*_max_
Competitive inhibition	↑	Unchanged
Non-competitive inhibition	Unchanged	↓
Uncompetitive inhibition	↓	↓
Mixed inhibition	↑	↓
Irreversible inhibition	Unchanged	↓

### 3.1. Irreversible inhibition

An irreversible inhibitor is a compound with a structure similar to the substrate or product that forms a covalent bond with a group present in the active centre. In the case of irreversible inhibition, it is not necessary to maintain the inhibitor concentration at a sufficient level to ensure enzyme-substrate interaction. The complex formed does not dissociate, so the enzyme is inactive even when the inhibitor is absent. In contrast to irreversible inhibition, reversible inhibition is characterised by dissociating the enzyme-inhibitor complex. There are the following types of reversible inhibition: competent, incompetent, acompetent, and mixed^18^.

#### 3.1.1. Competent inhibition

The inhibitor shows a structural similarity to the substrate with which it competes for access to the enzyme’s active centre. The enzyme-inhibitor complex converts to an enzyme-substrate complex, and the inhibitor is displaced from the enzyme’s active site. The degree of inhibition of the enzyme depends on the concentration of the substrate. The higher substrate concentration relative to the inhibitor, the fewer enzyme molecules will bind to the inhibitor. Therefore, the inhibition of the reaction caused by a competent inhibitor can be reversed by increasing the substrate concentration.

#### 3.1.2. Noncompetent inhibition

A noncompetent inhibitor usually bears no resemblance to the structure of the substrate. The inhibitor binds to the enzyme at a different site than the substrate. The inhibitor can attach to the free enzyme or the enzyme-substrate complex. Unlike competent inhibition, incompetent inhibition does not depend on the concentration of the substrate.

#### 3.1.3. Accompetent inhibition

The inhibitor binds to the enzyme-substrate complex, forming an enzyme-inhibitor-substrate complex.

#### 3.1.4. Mixed inhibition

It is a case of partially competent and incompetent inhibition. The inhibitor can bind to both the free enzyme and the ES complex, reducing the maximum rate^19^.

## 4. Inhibitors of hyaluronidase

### 4.1. Hyaluronic acid and hyaluronidase

**Figure 1.**
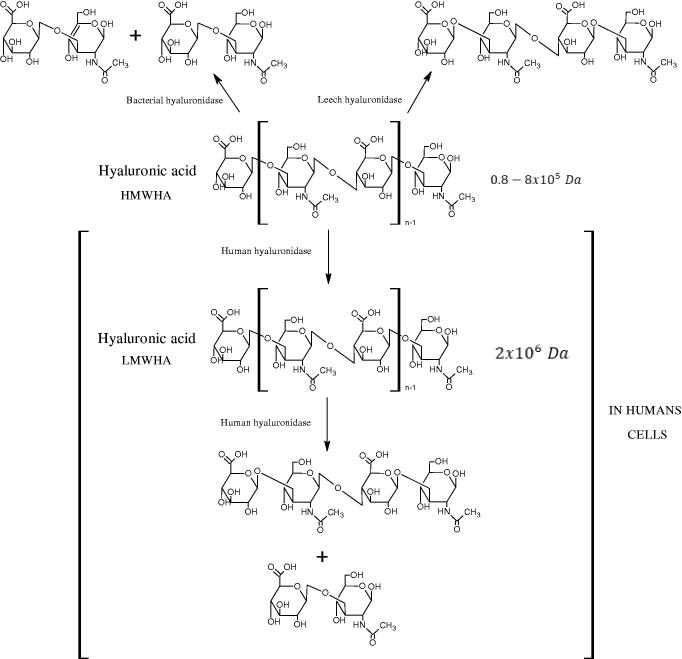
Classification of hyaluronidases (HMWHA: long-chain hyaluronic acid; LMWHA: short-chain hyaluronic acid).

Hyal is responsible for the hydrolysis of hyaluronic acid (HA). HA is a linear, high molecular weight unsulfated polysaccharide composed of alternating N-acetyl D-glucosamine and D-glucuronic acid residues linked by glycosidic bonds. That compound is present both in prokaryotes and eukaryotes, commonly found in many tissues and body fluids, such as muscles, joints, synovial fluid, skin, and vitreous body. In addition to its structural functions, it is involved in wound healing, inflammation, and tumour development. Hyals are a group of enzymes commonly found in nature, e.g. they are an important component of the venom of bees, spiders, and snakes^20–22^. Based on the structure and mechanism of action, there are three classes of Hyals (Figure 1). The first class includes endo-*β*-N-acetylhexosaminidases (EC 3.2.1.35) present in mammals and in snake and hymenoptera venom. They hydrolyse *β*-1,4-glycosidic bonds in HA. The second class is endo-*β*-D-glucuronidases (EC 3.2.1.36), which hydrolyse the *β*-1,3-glycosidic linkages in the HA and are present in invertebrates. As a result of the activity of both enzymes, tetra, and hexasaccharide of HA are formed. The third class includes endo-*β*-N-acetylhexosaminidases (EC 4.2.2.1), present only in prokaryotes. Bacterial enzymes break *β*-1,4-glycosidic bonds in HA using a *β*-elimination reaction. As a result of their action, unsaturated di-, tetra-, and hexasaccharide are formed. There are five types of Hyals found in humans: HYAL-1, HYAL-2, HYAL-3, HYAL-4, and HYAL-5. HYAL-1 and HYAL-2 are found in most tissues and are involved in the circulation of HA^23^. HYAL-2 degrades long-chain hyaluronic acid (HMWHA) found in the extracellular matrix to short-chain hyaluronic acid (LMWHA), which after binding to the CD44 receptor, is transported into the cell. HYAL-1 is found inside the cell and is responsible for the degradation of LMWHA into tetra and hexasaccharide^24^.

Hyals are involved in the regulation of important physiological processes, such as fertilisation and skin ageing. Present in sperm, HYAL-5 plays a key role in fertilisation in mammals as it enables the sperm to connect to the egg cell by breaking down HA in the granule layer. The overactivity of collagenases, Hyal, and elastase leads to the formation of wrinkles^25^.

Hyals are an important virulence factor for many bacterial species, such as *Staphylococcus spp.*, *Streptococcus spp.,* and *Streptomyces spp.* The decomposition of HA increases the viscosity of body fluids and reduces tissues’ integrity, facilitating the penetration of microorganisms and toxins into the skin^26^.

Hyals are an important component of the venom of Hymenoptera, spiders, and snakes. Hyal, present in the venom, helps to distribute the toxins throughout the body. Several studies have shown Hyal to be involved in tumour growth, metastasis, and angiogenesis^27^. However, the conducted studies do not provide unambiguous results showing that Hyals can function both as a suppressor and a promoter of carcinogenesis. HA promotes tumour metastasis, so the enzyme that breaks down hyaluronan (Hyal) inhibits their growth. On the other hand, low molecular weight HA stimulates angiogenesis, promoting tumour growth and metastasis formation^28^.

### 4.2. Polyphenols as inhibitors of hyaluronidase: structure–activity relationships (SARS)

#### 4.2.1. Flavonoids

Plant-based metabolites represent the chemically-different groups of compounds, of which many have been isolated for the first time from the plants used in the ethnomedicine of indigenous tribes. Those steps have allowed for their identification, biotechnological modification of their structure or to develop a synthesis path involving bacteria or fungi.

Phenolic compounds include a very numerous and important group of compounds commonly found in the world of plants. Polyphenols are plant secondary metabolites with very diverse chemical structures, containing at least two hydroxyl groups attached to an aromatic ring. Due to the number and way of connecting aromatic rings, we can distinguish phenolic acids, flavonoids, stilbenes, and lignans^29^^,^^30^. The presence of multiple hydroxyl groups gives phenolic compounds antioxidant properties^31^. Phenolic compounds also show antitumor, anti-inflammatory, antiviral, antibacterial, antifungal, hepatoprotective, antiallergic, anticoagulant, and blood vessel sealing properties^32^. Besides, many plant-derived polyphenols affect Hyal and other enzymes that regulate the metabolism of the extracellular matrix^33–36^.

**Figure 2.**
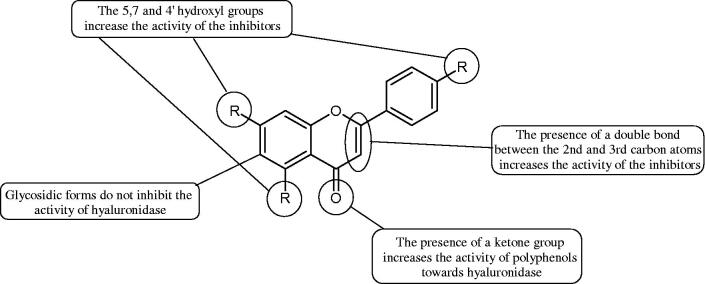
Chemical groups of flavonoids involved in the inhibition of hyaluronidase.

Inhibition of Hyal activity by polyphenolic compounds is related to, among others, the presence of hydroxyl groups (Figure 2). Hertel et al. investigated the effect of flavonoids with a different number of hydroxyl groups on Hyals’ activity [flavones (apigenin and luteolin) and flavonols (kaempferol and quercetin) in concentration 0.1 mM]. In the case of bovine testicular Hyal (4 U/mL), the activity of the tested compounds was low (less than 20% for apigenin and luteolin). The inhibition of Hyal was only slightly enhanced by hydroxyl groups (quercetin or myricetin). Phenolic compounds with an additional hydroxyl group in the 3-position (quercetin ca. 90% inhibition or myricetin – ca. 70% inhibition) more strongly inhibited *Streptococcus agalactiae* hyaluronan lyase. Additionally, the introduction of a sugar moiety significantly reduces the test compounds’ activity (e.g. rutin). Aglycones were more potent inhibitors than their corresponding glycosides, what may probably result from the inhibitor’s difficult access to the Hyal active site in case of glycosides^37^.

Another study^38^^,^^39^ determined the effect of luteolin, apigenin, kaempferol, myricetin, quercetin, and morine on bovine testicular hyaluronidase (BTH). Flavonoids containing a double bond between the 2 and 3 carbon atoms showed a greater potency than flavonoids without the double bond. Additionally, a ketone group in the 4-position or hydroxyl group at 5,7, and 4′ positions may increase the inhibition of Hyal. In turn, methoxylation of the 4′-OH group in hesperitin and diosmetin reduces their activity. On the other hand, the presence of the 3-OH group did not affect the potency of Hyal inhibition. In addition, the presence of a catechol system in the B ring (3'4′-OH) may show a beneficial effect on the inhibitory activity of flavonoids. As in other studies, glycoside substituent presence completely reverses the inhibitory effect of flavonoids on BTH. The compounds possessing the malonyl group in the C-6 position of the sugar moiety, such as apigenin-7-O-(6′′-O-malonyl)glucoside (IC_50_ = 360 µM) or luteolin-7-O-(6′′-O-malonyl)glucoside (IC_50_ = 324 µM) show stronger inhibitory properties than compounds lacking this group, i.e. apigenin-7-O-rutinoside (IC_50_ > 1000 µM), naringenin7-O-rutinoside (IC_50_ > 1000 µM) and luteolin-7-O-glucoside (IC_50_ = 695 µM)^40^. In another study, a relationship between the position of the sugar moiety and an activity against Hyal was found [apigenin 7-O-(3′’-O-acetyl)-glucuronide (200 µM, inhibition 10.0%) and apigenin 5-O-(3′’-O-acetyl)-glucuronide (200 µM, inhibition 13.9%)]^41^.

Compounds containing a sugar residue in the C-3 position weakly block Hyal [kaempferol-3-O-α-L-rhamno pyranoside (18.26%), quercetin-3-O-α-L-rhamno pyranoside (6.88%), and quercetin-3-O-α-L-arabino pyranoside (13.81%)] (Table 2)^42^.

**Table 2.** Structure of flavonoid glycosides with an anti-hyaluronidase activity.

Structure	Name	IC_50_ (μM)	References
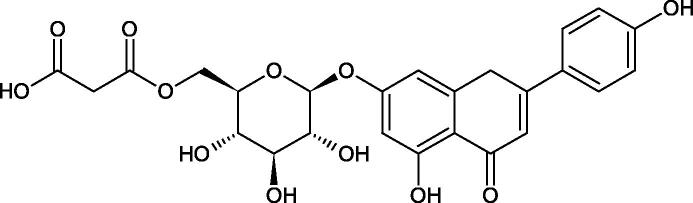	Apigenin-7-O-(6′’-O-malonyl)glucoside	360	Aoshima et al.^40^
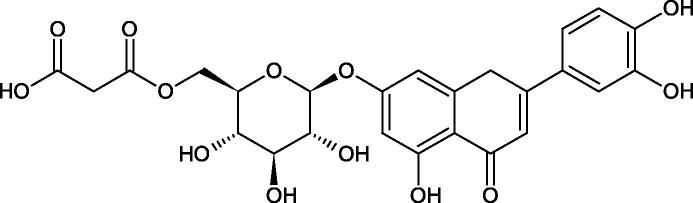	Luteolin-7-O-(6′’-O-malonyl)glucoside	324	Aoshima et al.^40^
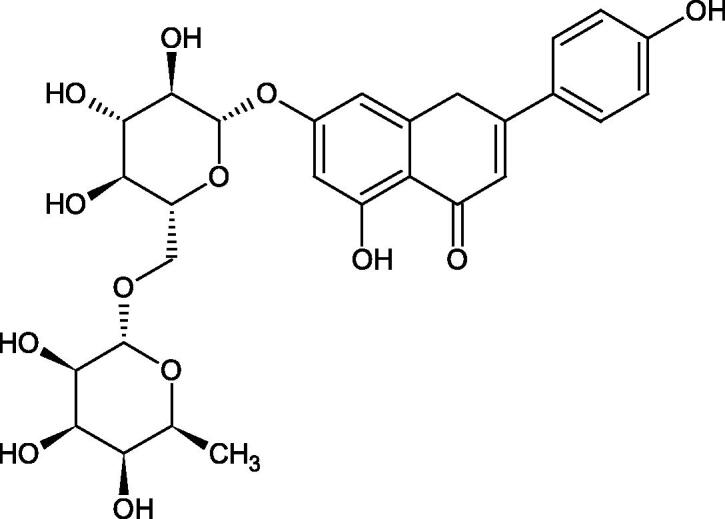	Apigenin-7-O-rutinoside	>1000	Aoshima et al.^40^
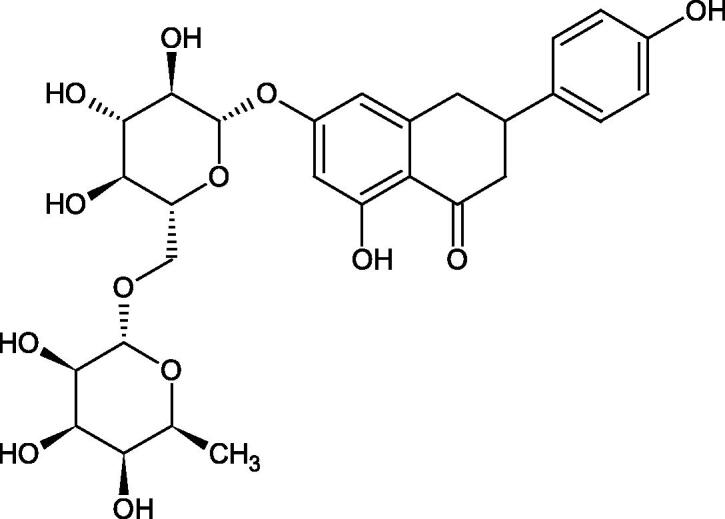	Naringenin7-O-rutinoside	>1000	Aoshima et al.^40^
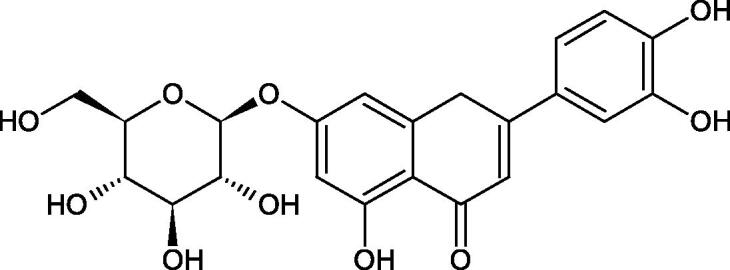	Luteolin-7-O-glucoside	695	Aoshima et al.^40^
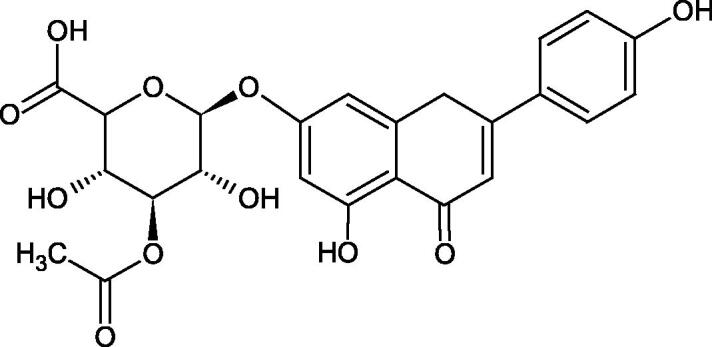	Apigenin 7-O-(3′’-O-acetyl)-glucuronide	–	Kubínová et al.^41^
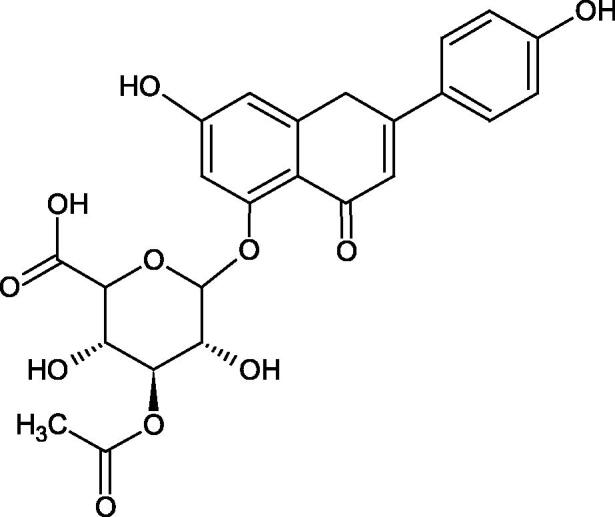	Apigenin 5-O-(3′’-O-acetyl)-glucuronide	–	Kubínová et al.^41^
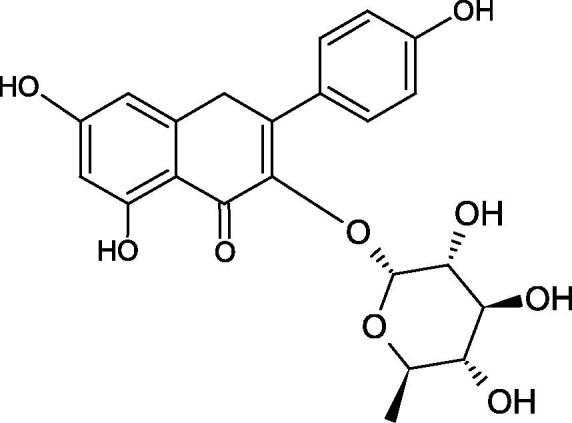	Kaempferol-3-O-α-L-rhamno pyranoside	–	Karakaya et al.^42^
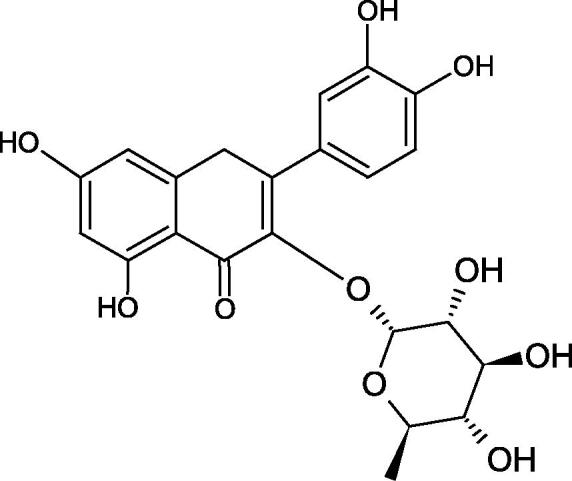	Quercetin-3-O-α-L-rhamno pyranoside	–	Karakaya et al.^42^
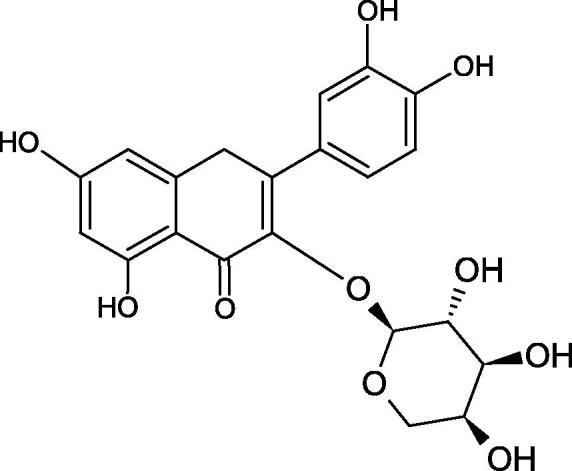	Quercetin-3-O-α-L-arabino pyranoside	–	Karakaya et al.^42^

Another possible mechanism involved in an inhibition of Hyals’ activity is based on the ability to associate compounds of low molecular weight. The acid function (hydroxyl, carboxyl, phosphate, or sulphate) is necessary to form an aggregate with multiple negative charges. More research is needed to determine the possibility of aggregating by low molecular weight flavonoids that can act as effective inhibitory units.

Bralley et al. determined antioxidants’ influence (phenolic acids, flavonoids, and condensed tannins) in Sorghum on Hyal activity. Amongst tested compounds, i.e. condensed tannin, apigenin, luteolin, kaempferol, quercetin, and rutin, only the four first were effective. Probably condensed tannins not only denature the enzyme but also interact with the hydrophobic channel^43^. Tatemoto et al. investigated the effect of tannin, apigenin, and quercetin on Hyal activity and the fertilisation process *in vitro*. Tannins showed the greatest inhibitory properties at concentrations of 2–10 µg/mL. These data suggest that an adequate concentration of tannic acid prevents polyspermia by inhibiting sperm Hyal activity during IVF of porcine oocytes. However, the presence of apigenin or quercetin in the same concentrations as tannic acid could not prevent polyspermy^44^.

The study by Zeng et al. determined how apigenin, luteolin, kempferol, quercetin, morin, naringenin, daidzein, and genistein bind to HAase. It was shown that those compounds interacted with the active centre of the enzyme through electrostatic forces, hydrophobic interactions, and hydrogen bonds. The binding of flavonoids caused changes in the active centre structure, resulting in inhibition of HAase (Table 3)^45^.

**Table 3.** Structures of the active compounds.

		Substituent			
Flavones	Compounds	$R_{1}$	$R_{2}$	$R_{3}$	$R_{4}$
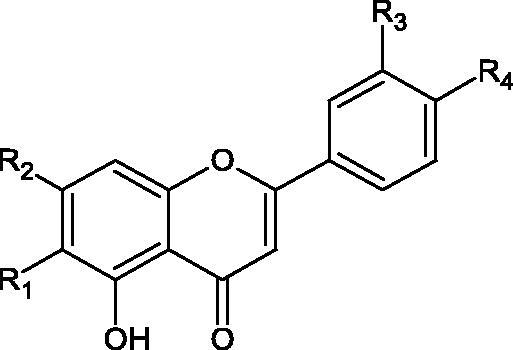	Apigenin	H	OH	H	OH
	Luteolin	H	OH	OH	OH
	Baicalein	OH	OH	H	H
	Baicalin	OH	Glucuronide	H	H
Flavonols					
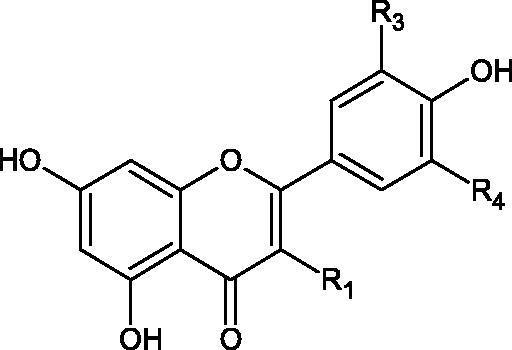	Kaempferol	OH		H	H
	Quercetin	OH		OH	H
	Myricetin	OH		OH	OH
	Rutin	Rutinose		H	OH
Flavanones					
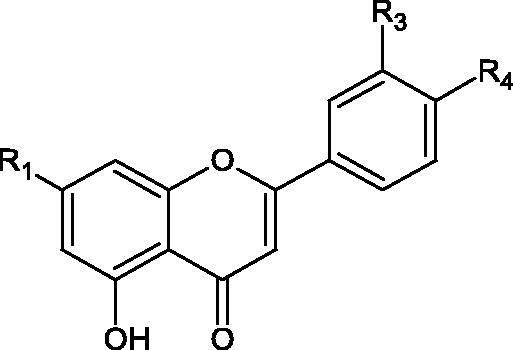	Naringenin	OH		H	OH
	Hesperetin	OH		OH	$O{CH}_{3}$
	Naringin	Neohesperidose		H	OH
	Hesperidin	Rutinoside		OH	$O{CH}_{3}$
Isoflavone					
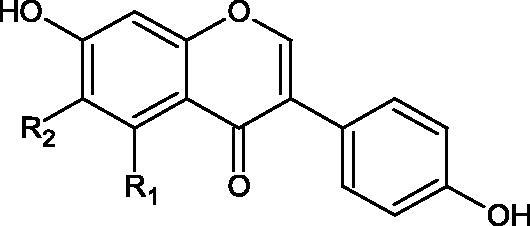	Genistein	OH	H		
Flavan-3-ols					
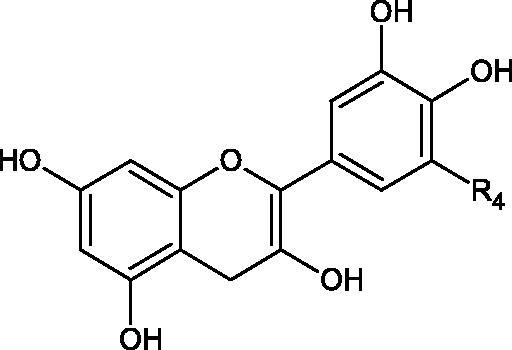	Catechin				H
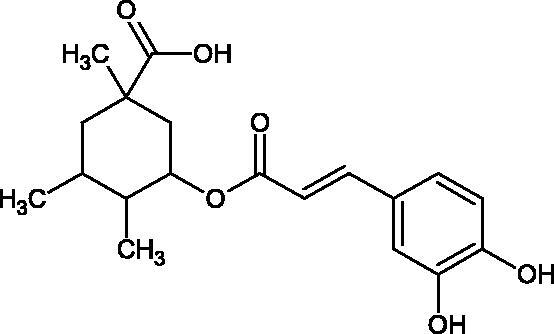	Chlorogenic acid				

#### 4.2.2. Phenolic acids

Phenolic acids have demonstrated potent anti-Hyal properties. The impact of rosmarinic acid (IC_50_ = 24.3 µg/mL), protocatechuic acid (IC_50_ = 107.6 µg/mL), ferulic acid (IC_50_ = 396.1 µg/mL), and chlorogenic acid (IC_50_ = 162.4 µg/mL) on the activity of Hyal was noted.

Iwanaga et al. investigated the composition and effects of aqueous-acetone extracts from the aerial parts of *Cimicifuga simplex* and *Cimicifuga japonica* on HAase. The newly isolated fukiic acid derivatives (IC_50_ of compound 1. 255 µM; 2. 102 µM; 3. 173 µM; 4. 120 µM) inhibited Hyal more potently than rosmarinic acid (IC_50_ = 545 µM), caffeic acid (IC_50_ > 2000 µM), ferulic acid (IC_50_ > 2000 µM) and isoferulic acid (IC_50_ > 2000 µM). Based on the structure of compounds nr 2 and 4, the methoxy groups at the C-3′′ and C-4′′ positions may participate in the Hyal inhibition^46^.

Oligomers composed of caffeic acid exhibited interesting anti-Hyal properties. Caffeic acid trimer isolated from *Dracocephalum foetidum* inhibited Hyal more strongly than disodium cromoglycate (IC_50_ = 220 µM and IC_50_ = 650 µM). Similar results were obtained by Aoshima’s team studying coffee acid oligomers isolated from *Clinopodium gracile*. It was appeared that coffee acid oligomers, such as clinopodic acid M, showed more potent inhibitory activity than rosmarinic acid (IC_50_ = 19 and 226 µM, respectively). The most active compounds possessed 3–(3,4-dihydroxyphenyl)-2-hydroxypropionic acid – danshensu grouping. The activity of other compounds with the structure of danshensu amounted to: clinopodic acid E (IC_50_ = 40 µM), clinopodic acid I (IC_50_ = 112 µM), clinopodic acid K(IC_50_ = 63 µM), clinopodic acid L(IC_50_ = 26 µM), clinopodic acid N(IC_50_ = 161 µM), clinopodic acid O(IC_50_ = 66 µM), clinopodic acid P(IC_50_ = 25 µM), clinopodic acid Q(IC_50_ = 165 µM), lithospermic acid (IC_50_ = 36 µM), salvianolic acid B (IC_50_ = 107 µM), and salvianolic acid A (IC_50_ = 206 µM). The IC_50_ values for compounds without the danshensu structure were equal 206 and 653 µM for clinopodic acid J and 8-epiblechnic acid, respectively. An interesting structure for further studies may be clinopodic acid E, which has instead of the structure of 2,3-dihydrobenzofuran, 1,4-benzodioxane. This compound is four times more potent than the analogue having the structure of 2,3-dihydrobenzofuran (clinopodic acid N). Additionally, as in other studies, a beneficial effect of the increase in oligomer mass on the inhibition of Hyal activity is seen^40^^,^^47^.

**Figure 3.**
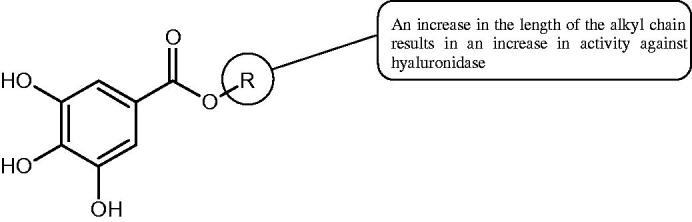
Effect of alkyl chain length in phenolic acids on activity against hyaluronidase.

**Figure 4.**
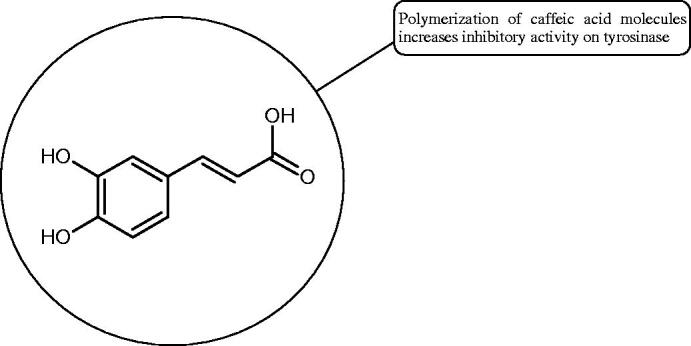
Potential groups engaged in an interaction oligomers phenolic acids-hyaluronidase.

The structure of 1,4-benzodioxane is present in clinopodic acid C (IC_50_ = 80.1 µM) and clinopodic acid E (IC_50_ = 82.8 µM), isolated from the herbal drug takuran, which is produced from *Lycopus lucidas*. These compounds inhibited Hyal more strongly than rosmarinic acid (IC_50_ = 309 µM). It was found that an esterification of the carboxyl group associated with the structure of 1,4-benzodioxane decreased the activity of the compounds (lycopic acid A; IC_50_ = 134 µM; lycopic acid B; IC_50_ = 141 µM). Rosmarinic acid oligomers (trimer IC_50_ = 275 µM; tetramer IC_50_ = 183 µM) blocked Hyal more potent than rosmarinic acid (IC_50_ = 309 µM). Activity against hyalurinidase is also shown by seric acid A (IC_50_ = 119 µM), F (IC_50_ = 1330 µM), and G (IC_50_ = 1270 µM) isolated from the *Oenanthe javanica* root (Figures 3 and 4) (Table 4)^48^.

**Table 4.** Structure and activity of phenolic acids against hyaluronidase.

Structure	Name	IC_50_(μM)	References
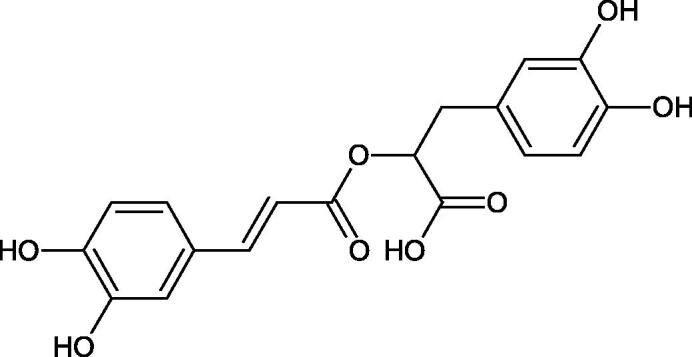	Rosmarinic acid	545	Iwanaga et al.^46^
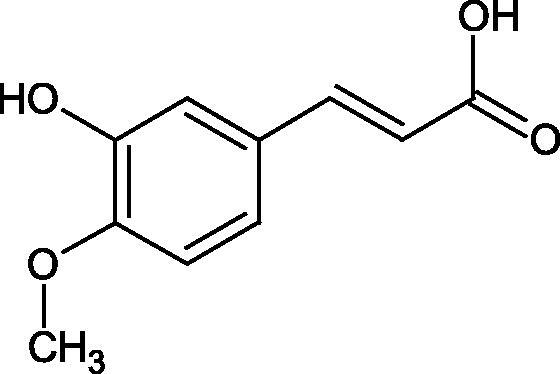	Isoferulic acid	>2000	Iwanaga et al.^46^
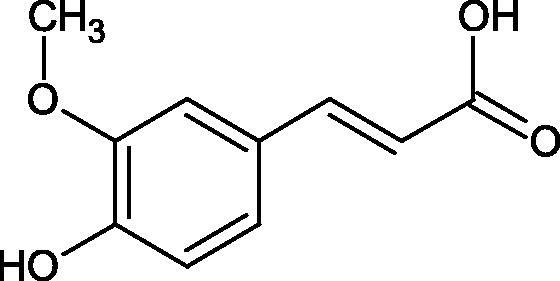	Ferulic acid	>2000	Iwanaga et al.^46^
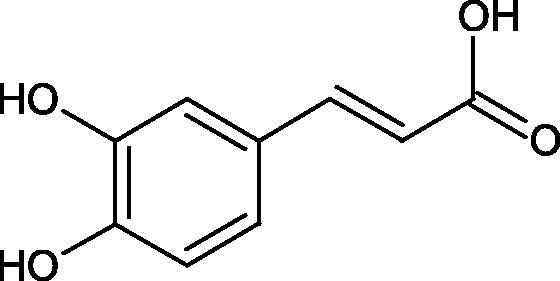	Caffeic acid	>2000	Iwanaga et al.^46^
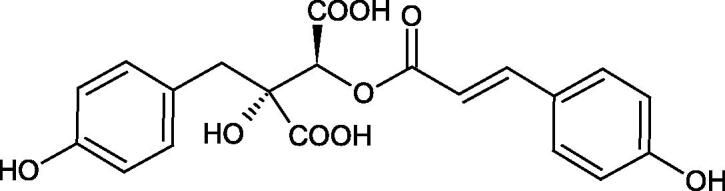	Cimicifugic acid K	255	Iwanaga et al.^46^
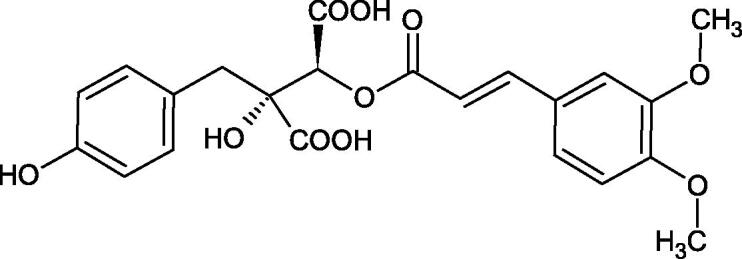	Cimicifugic acid L	102	Iwanaga et al.^46^
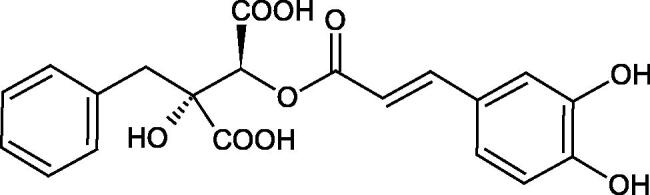	Cimicifugic acid M	173	Iwanaga et al.^46^
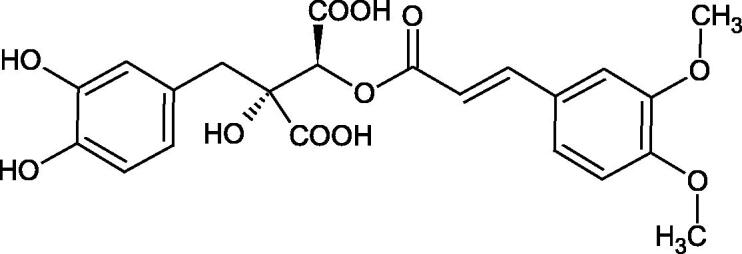	Cimicifugic acid N	120	Iwanaga et al.^46^
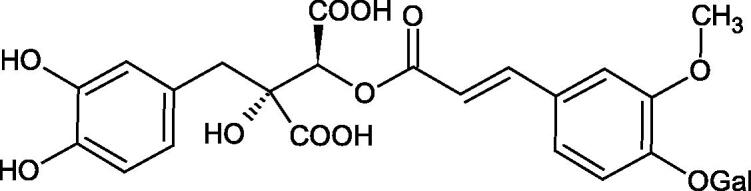	Shomaside A	573	Iwanaga et al.^47^
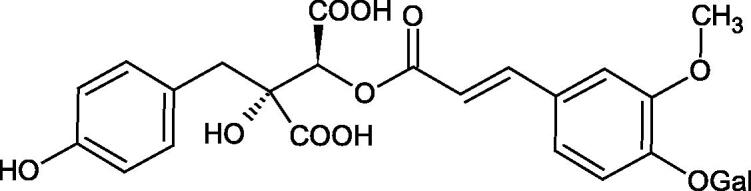	Shomaside B	430	Iwanaga et al.^47^
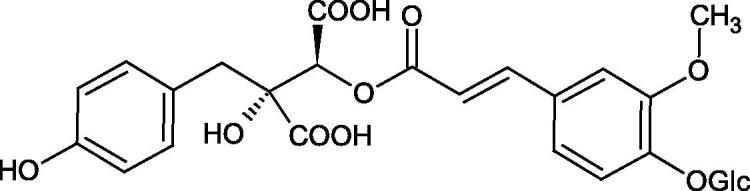	Shomaside C	663	Iwanaga et al.^47^
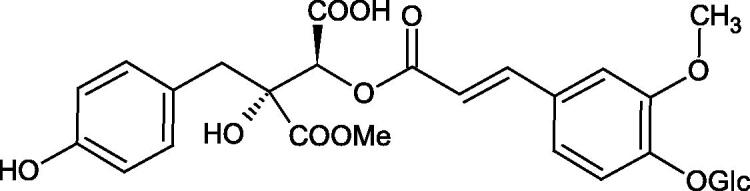	Shomaside D	>658	Iwanaga et al.^47^
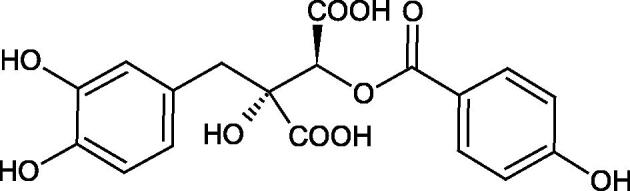	Cimicifugic acid H	525	Iwanaga et al.^47^
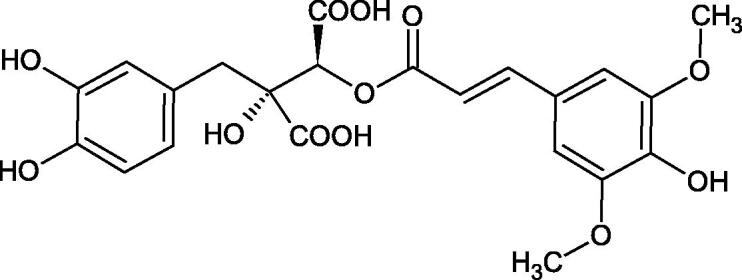	Cimicifugic acid I	143	Iwanaga et al.^47^
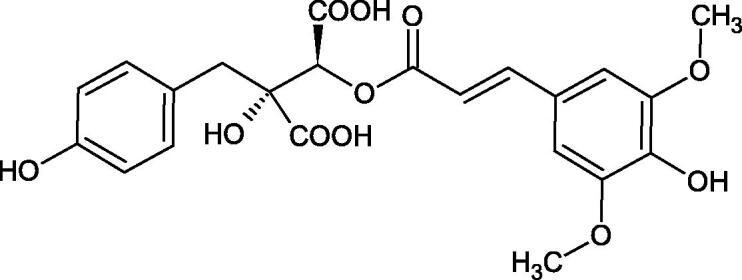	Cimicifugic acid J	193	Iwanaga et al.^47^
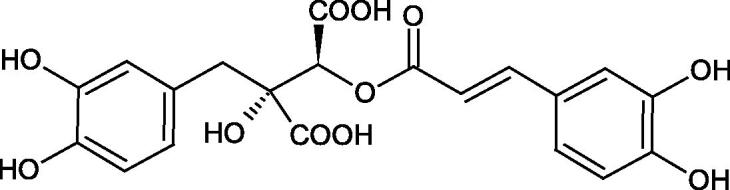	Fukinolic acid	144	Iwanaga et al.^47^
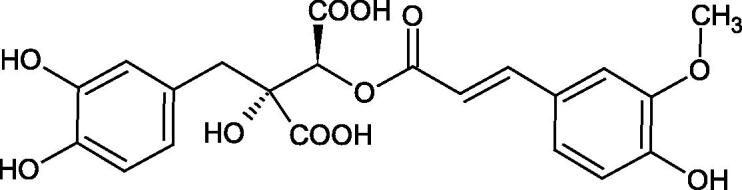	Cimicifugic acid A	112	Iwanaga et al.^47^
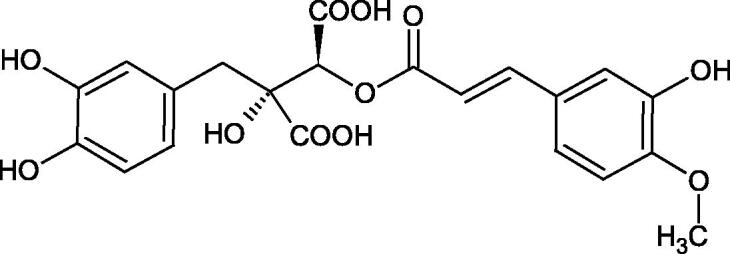	Cimicifugic acid B	82	Iwanaga et al.^47^
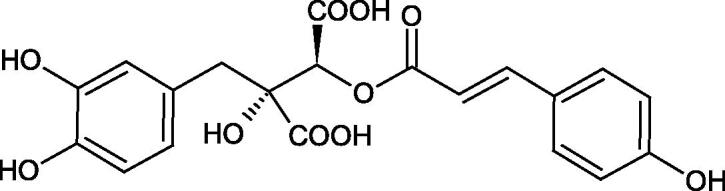	Cimicifugic acid C	251	Iwanaga et al.^47^
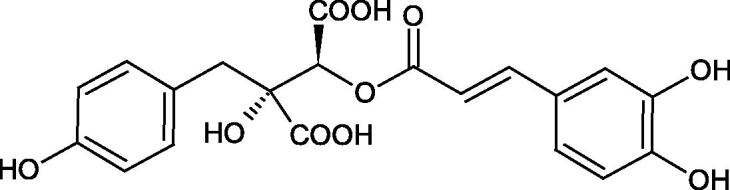	Cimicifugic acid D	153	Iwanaga et al.^47^
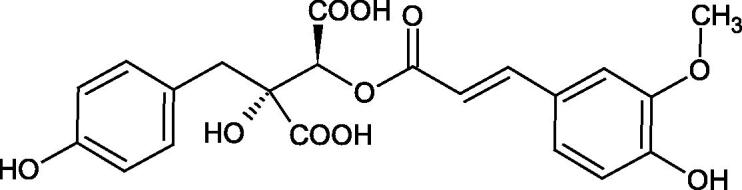	Cimicifugic acid E	120	Iwanaga et al.^47^
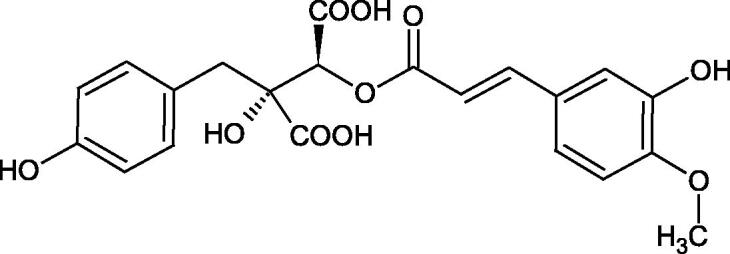	Cimicifugic acid F	92	Iwanaga et al.^47^
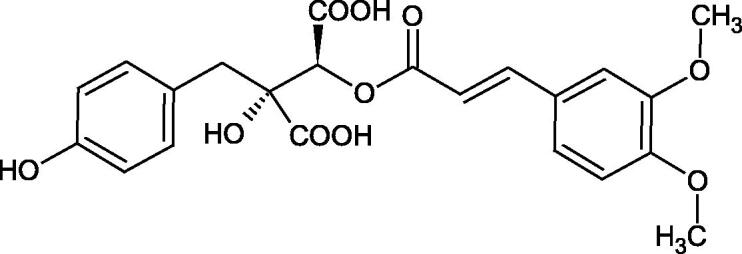	Cimicifugic acid G	138	Iwanaga et al.^47^
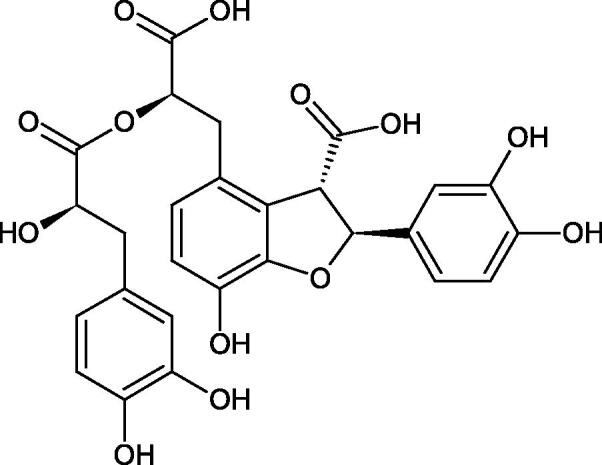	Clinopodic J	206	Aoshima et al.^40^
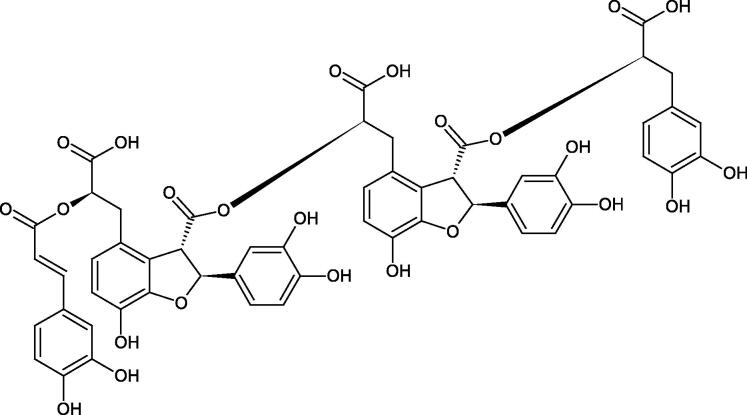	Clinopodic K	63	Aoshima et al.^40^
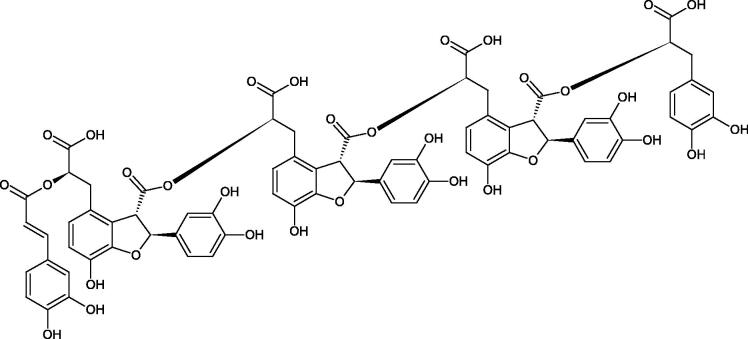	Clinopodic L	26	Aoshima et al.^40^
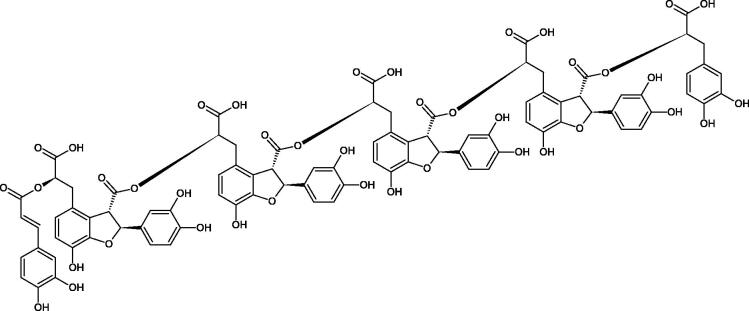	Clinopodic M	19	Aoshima et al.^40^
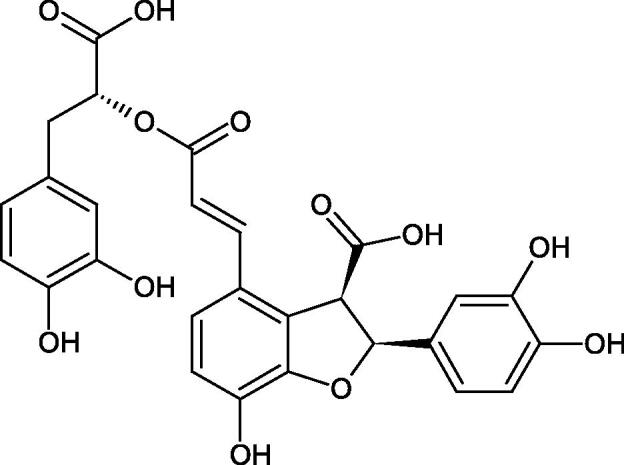	Clinopodic N	161	Aoshima et al.^40^
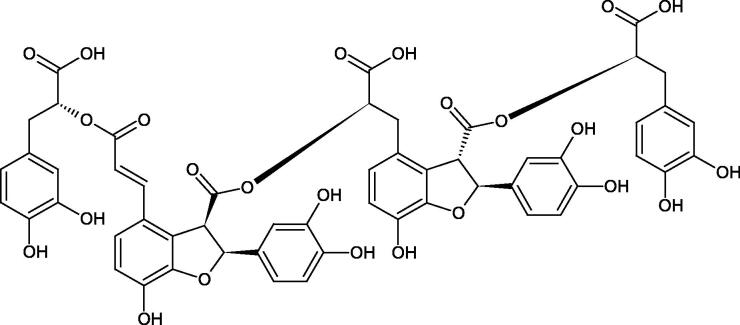	Clinopodic O	66	Aoshima et al.^40^
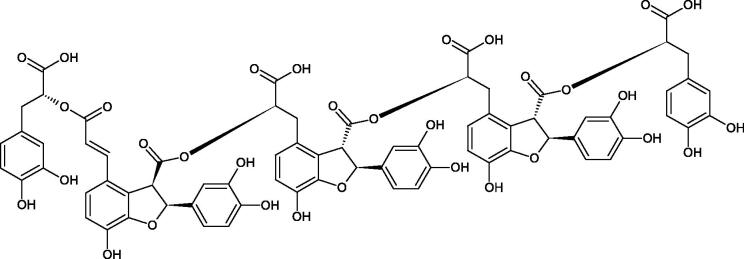	Clinopodic P	25	Aoshima et al.^40^
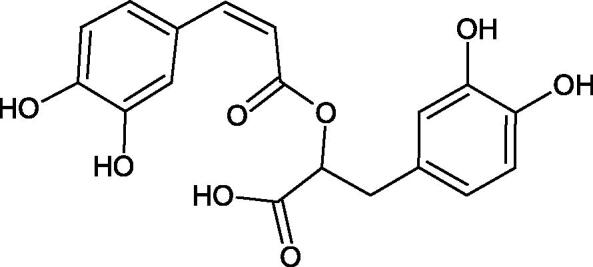	Rosmarinic acid		Aoshima et al.^40^
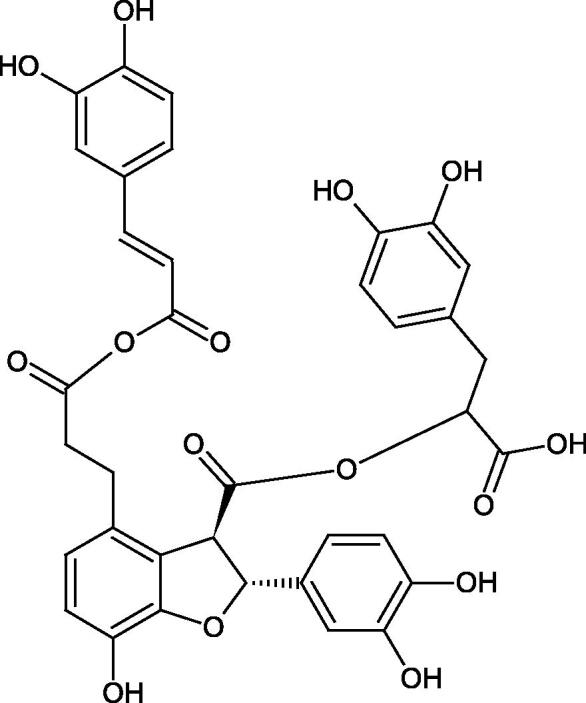	Clinopodic acid I	112	Aoshima et al.^40^
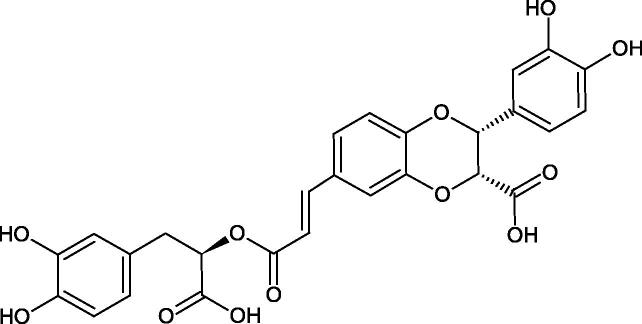	Clinopodic acid E	40	Aoshima et al.^40^
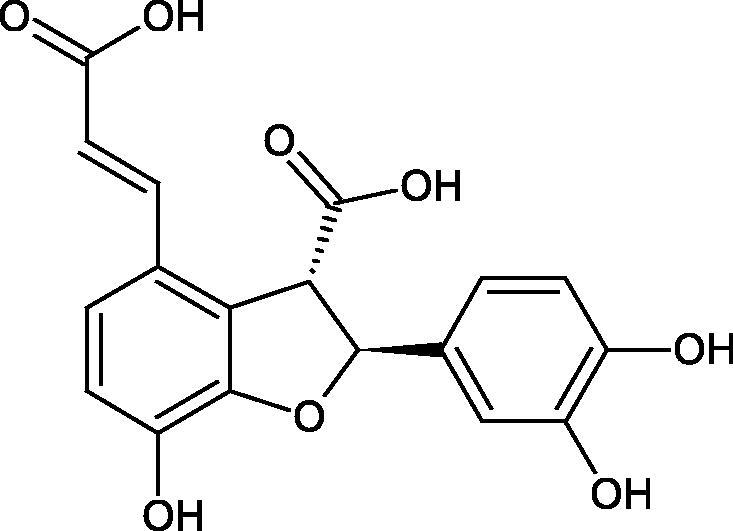	8-Epiblechnic acid	653	Aoshima et al.^40^
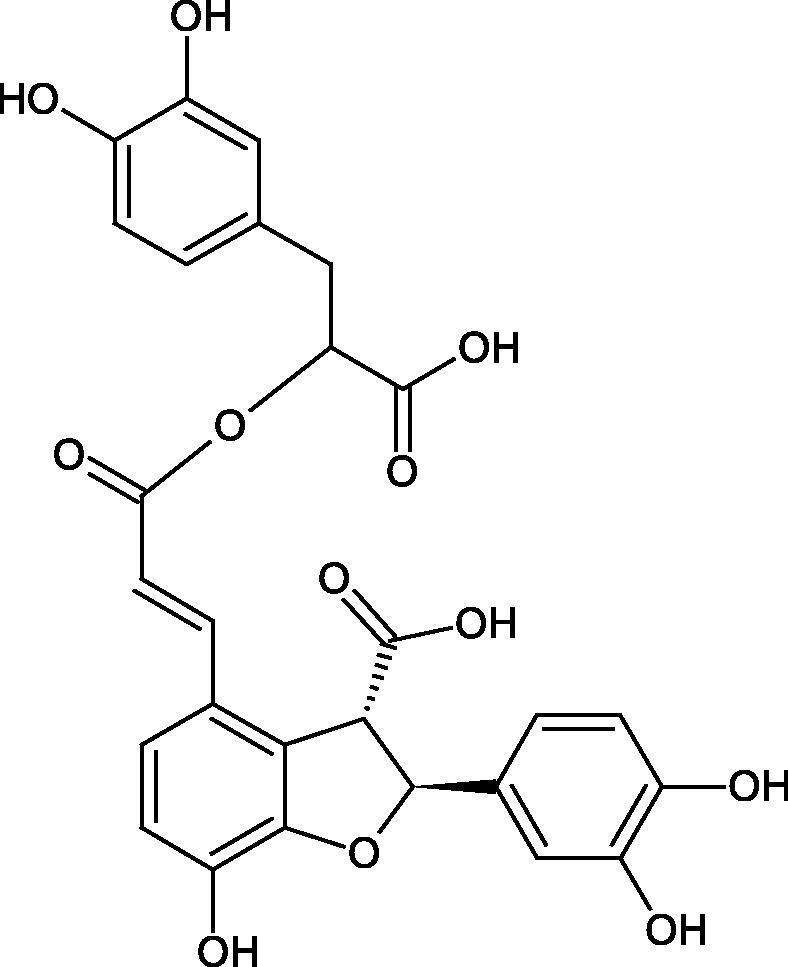	Lithospermic acid	36	Aoshima et al.^40^
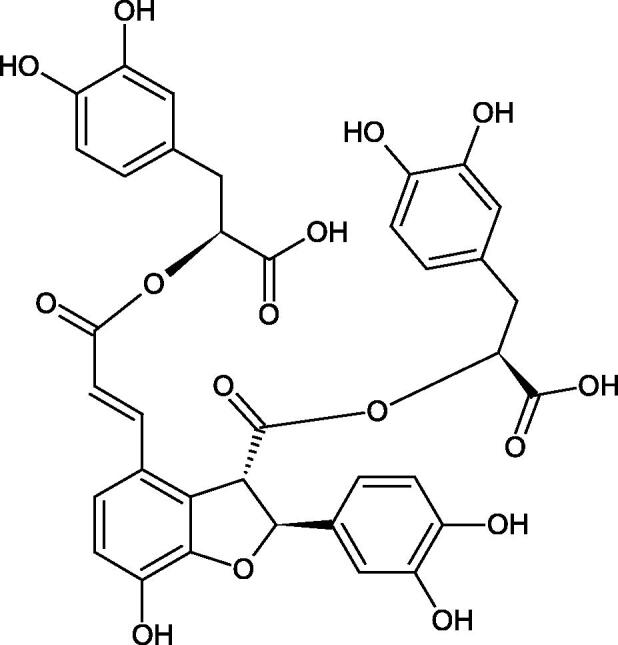	Salvianolic acid B	107	Aoshima et al.^40^
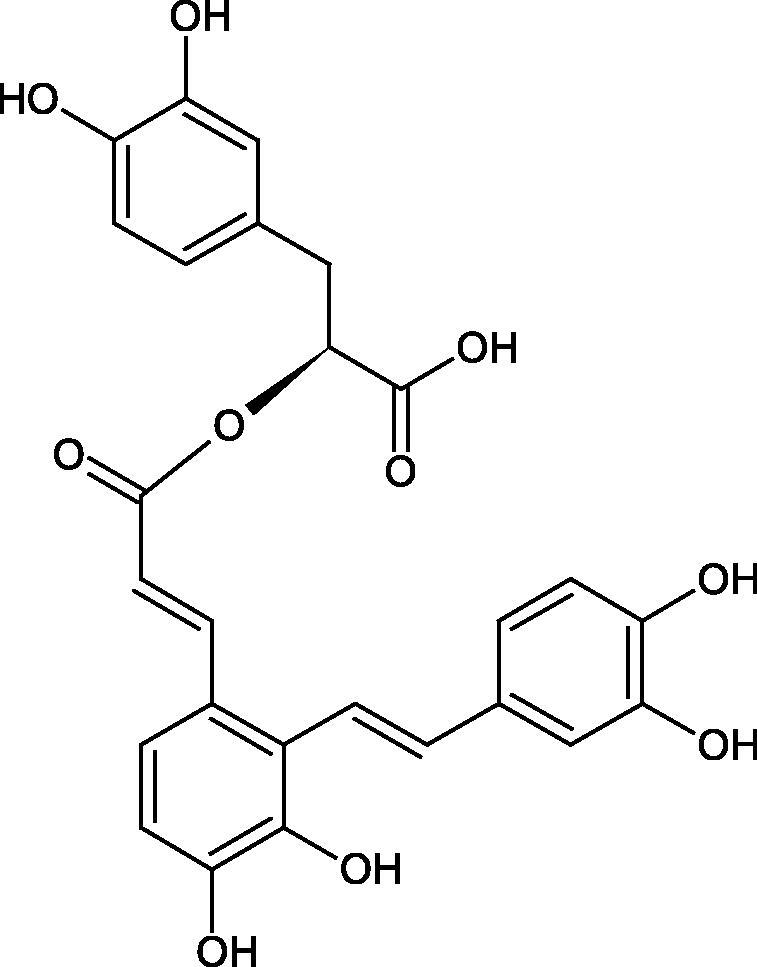	Salvianolic acid A	206	Aoshima et al.^40^
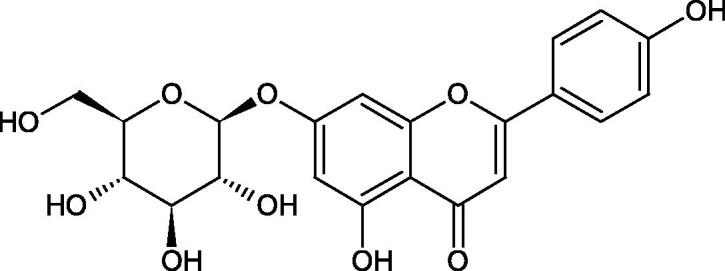	Cosmosiin	>1000	Aoshima et al.^40^

#### 4.2.3. Tannins

**Figure 5.**
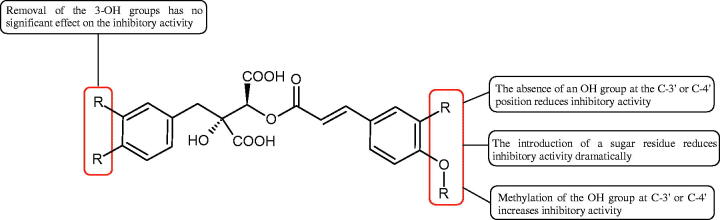
Potential groups engaged in an interaction fukiic acid derivative-hyaluronidase.

Tannins are nitrogen-free plant substances of high molecular weight (500 − 3000), having numerous hydroxyl groups. Due to their chemical structure, they are divided into two groups: hydrolysing and non-hydrolysing (condensed) (Figure 5). The first group is divided into galotannins (ester combinations of gallic acid and its derivatives) or elagotannins (ester combinations of ellagic acid). The second group is formed by the condensation of catechins (flavan-3-ol products). Tannins show the ability to form complexes with proteins, resulting in an astringent effect on the skin and mucous membranes. Tannins have been shown to have many properties, such as antibacterial, antiviral, anticancer, antioxidant, anti-inflammatory, and anti-hemorrhagic^49–51^.

Sugimoto et al. investigated the effect of hydrolysing tannins isolated from an ethanolic extract of *Eucalyptus globulus* Labill. on Hyal activity (400 units/mL; from bovine testis Type IV-S). The following tannins were used in the study: pedunculagin (${\mathrm{IC}}_{50}$= 1.51 mM), tellimagrandin I (${\mathrm{IC}}_{50}$= 0.9 mM), tellimagrandin II (${\mathrm{IC}}_{50}$= 0.58 mM), heterophylliin A (${\mathrm{IC}}_{50}$= 0.89 mM), 1,3-di-O-galloyl-4,6-hexahydroxydiphenoyl-*β*-D-glucose (${\mathrm{IC}}_{50}$= 0.74 mM), 1,2,4-tri-O-galloyl-*β*-D-glucose (${\mathrm{IC}}_{50}$= 1.57 mM), 1,2,3,6-tetra-O-galloyl-*β*-D-glucose (${\mathrm{IC}}_{50}$= 0.35 mM), 1,2,4,6-tetra-O-galloyl-*β*-D-glucose (${\mathrm{IC}}_{50}$= 0.68 mM), 1,2,3,4,6-penta-O-galloyl-*β*-D-glucose (${\mathrm{IC}}_{50}$= 0.55 mM), ellagic acid (${\mathrm{IC}}_{50}$= 4.66), gallic acid (${\mathrm{IC}}_{50}$= 5.0 mM), and disodium cromoglycate (${\mathrm{IC}}_{50}$= 0.45 mM) as a control. The activity of the tested compounds increases with the number of gallic acid residues attached to the sugar grouping (1,2,4-tri-O-galloyl-*β*-D-glucose − ${\mathrm{IC}}_{50}$= 1.57 mM, 1,2,4,6-tetra-O-galloyl-*β*-D-glucose − ${\mathrm{IC}}_{50}$= 0.68 mM, 1,2,3,4,6-penta-O-galloyl-*β*-D-glucose − ${\mathrm{IC}}_{50}$= 0.55 mM). Also, the localisation of gallic acid residues affects the inhibitory activity (1,2,3,6-tetra-O-galloyl-*β*-D-glucose − ${\mathrm{IC}}_{50}$= 0.35 mM *vs.* 1,2,4,6-tetra-O-galloyl-*β*-D-glucose − ${\mathrm{IC}}_{50}$= 0.68 mM). The inhibition level was similar for both ellagotannins and gallotannins. Another gallotannin, agrimoniin (${\mathrm{IC}}_{50}$= 2.65 µM), also showed strong inhibition of Hyal activity^52^.

**Figure 6.**
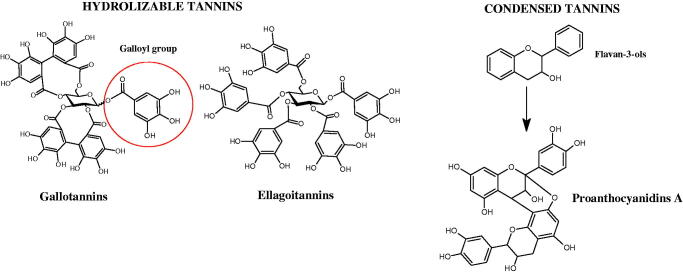
Chemical classification of tannins.

In another study^53^, gallic acid esters with different n-alkanol chain lengths (from C-1 to C-12) were examined to determine their inhibitory activity against Hyal. With an increase of the alkyl chain length, the inhibitory activity was increased. Cromoglycan disodium (${\mathrm{IC}}_{50}$= 450 µM) was used as a positive control. Hexyl (${\mathrm{IC}}_{50}$= 253 µM), heptyl (${\mathrm{IC}}_{50}$= 112 µM), octyl (${\mathrm{IC}}_{50}$= 106 µM), nonyl (${\mathrm{IC}}_{50}$= 167 µM), and decyl (${\mathrm{IC}}_{50}$= 580 µM) gallates inhibited Hyal. Next, the impact of hydroxyl groups in octyl gallate on an activity was checked. Octyl 3-hydroxybenzoate and octyl 4-hydroxybenzoate did not inhibit Hyal. Octyl 3,4-dihydroxybenzoate (${\mathrm{IC}}_{50}$= 902 µM) blocked the enzyme to a small extent. The strongest inhibitor was octyl 3,5-dihydroxybenzoate (${\mathrm{IC}}_{50}$= 113 µM). This shows the significance of the 3,5-OH grouping in gallic acid (Table 5). The type of inhibition was determined only for octyl gallate, which inhibited the enzyme in a competitive manner. The *K_i_* value was estimated to be 45 µM (Figure 6).

**Table 5.** Structures and activity of tannins and their esters with n-alkanol chain lengths against hyaluronidase.

Structure	Compound	$R_{1}$	$R_{2}$	$R_{3}$	$R_{4}$	${IC}_{50}$ (µM)	References
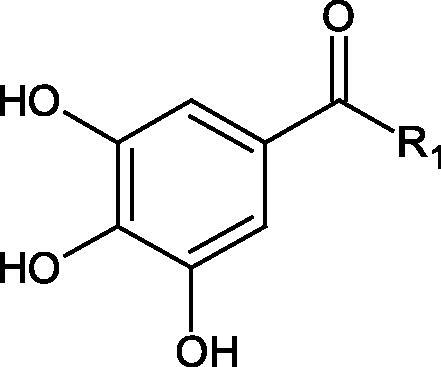	Gallic acid	H				>1000	Barla et al.^53^
	Methyl gallate	O${\mathrm{CH}}_{3}$				>1000	
	Ethyl gallate	O${\mathrm{CH}}_{2}{\mathrm{CH}}_{3}$				>1000	
	Propyl gallate	${{O(CH}_{2})}_{2}{CH}_{3}$				>1000	
	Butyl gallate	${{O(CH}_{2})}_{3}{CH}_{3}$				>1000	
	Hexyl gallate	${{O(CH}_{2})}_{5}{CH}_{3}$				253	
	Heptyl gallate	${{O(CH}_{2})}_{6}{CH}_{3}$				112	
	Octyl gallate	${{O(CH}_{2})}_{7}{CH}_{3}$				106	
	Nonyl gallate	${{O(CH}_{2})}_{8}{CH}_{3}$				167	
	Decyl gallate	${{O(CH}_{2})}_{9}{CH}_{3}$				580	
	Dodecyl gallate	${{O(CH}_{2})}_{11}{CH}_{3}$				>1000		
	
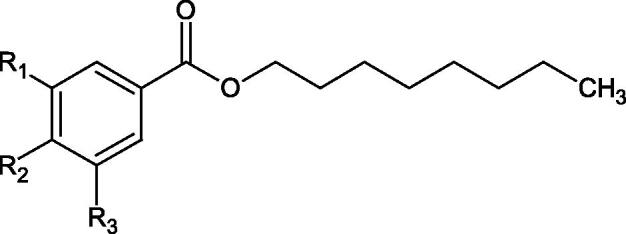	Octyl 3-hydroxybenzoate	OH	H	H		>1000		
	Octyl 4-hydroxybenzoate	H	OH	H		>1000		
	Octyl 3,4-dihydroxybenzoate	OH	OH	H		902		
	Octyl 3,5-dihydroxybenzoate	OH	H	OH		113		
	
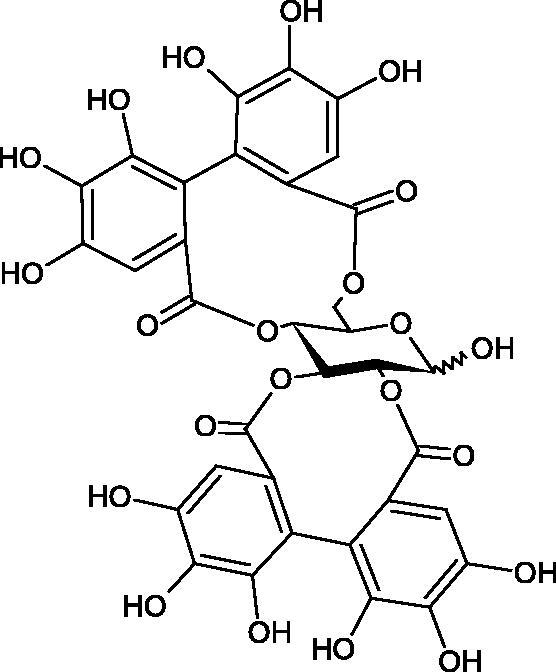	Pedunculagin					1.51	Tokeshi et al.^54^	
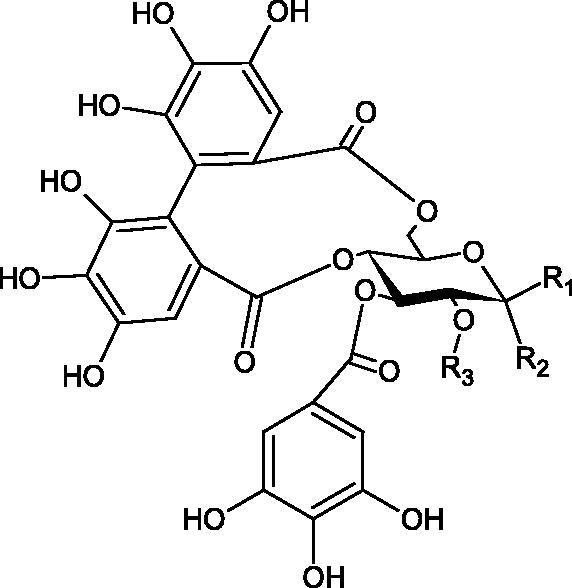	Tellimagrandin I	H	OH	G		0.9	Sugimoto et al.^52^	
	Tellimagrandin II	OG	H	G		0.58		
	Heterophylliin A	H	OG	H		0.89		
	1,3-Di-O-galloyl-4,6-hexahydroxydiphenoyl-β-D-glucose	OG	H	H		0.74		
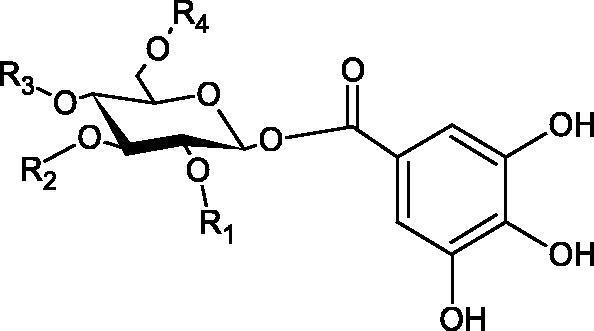	1,2,4-Tri-O-galloyl-β-D-glucose	G	H	G	H	1.57		
	1,2,3,6-Tetra-O-galloyl-β-D-glucose	G	G	H	G	0.35		
	1,2,4,6-Tetra-O-galloyl-β-D-glucose	G	H	G	G	0.68		
	1,2,3,4,6-Penta-O-galloyl-β-D-glucose	G	G	G	G	0.55		
	
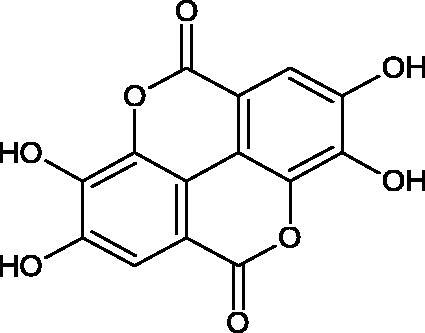	Ellagic acid					4.66		
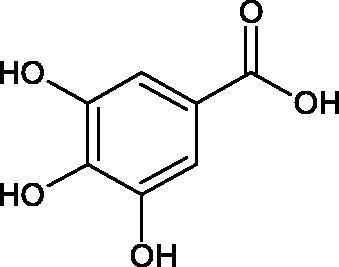	Gallic acid					5.00		
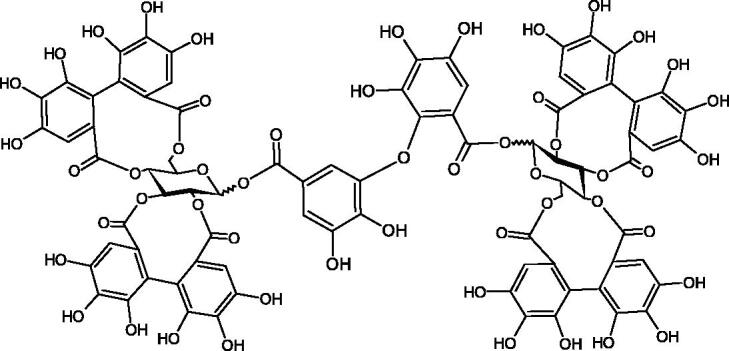	Agrimoniin					2.65		
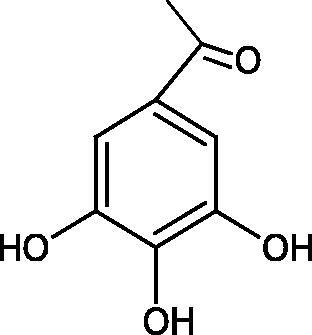	Galloyl group (G)							
Phloroglucinol	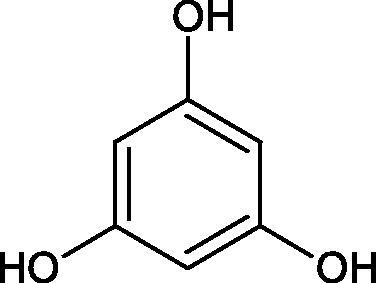							
Phloroglucinol tetramer	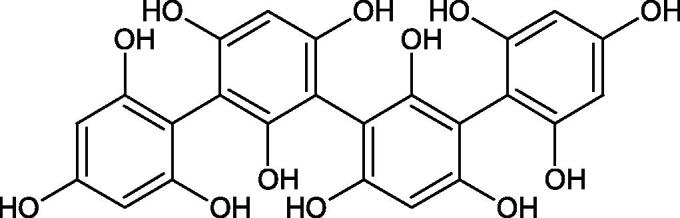							
Phlorofucofuroeckol A	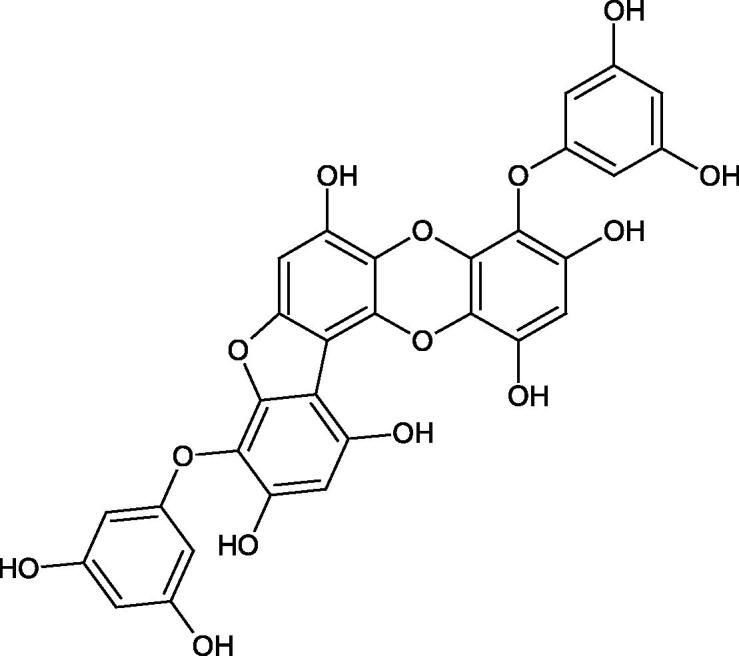							
Dieckol	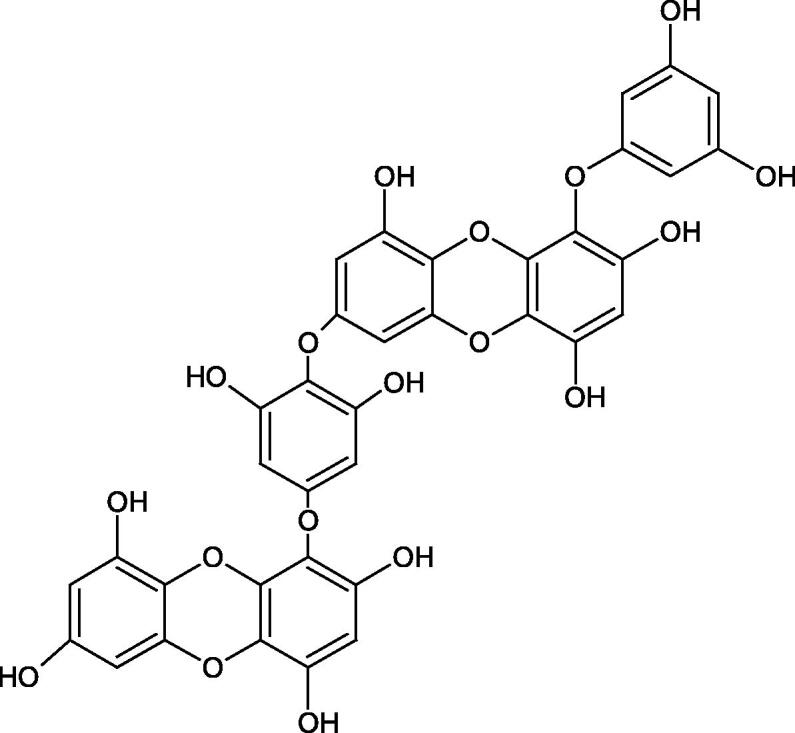							
8,8′-Bieckol	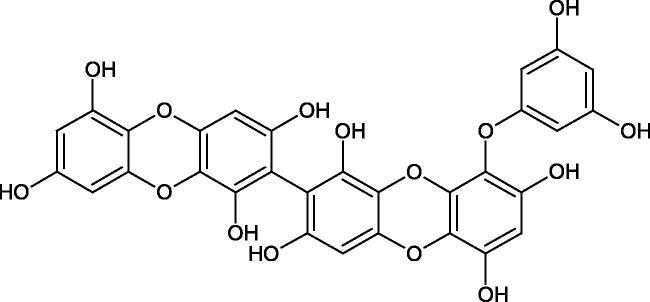							

Tokeshi et al. examined the effect of three tannins (tannic acid TA, gallic acid GA, and ellagic acid EA) on boar sperm Hyal. TA and EA strongly inhibited Hyal in the concentration range of 2–10 µM^54^. The phlorotannins present in *Eisenia bicyclis* and *Ecklonia kurome* inhibited Hyal activity more strongly than standard substances such as disodium cromoglycate (IC_50_ = 270 µM), catechin (IC_50_ = 620 µM), and epigallocatechin gallate (IC_50_ = 190 µM). The IC_50_ values for phloroglucinol, phloroglucinol tetramer, eckol (trimer), phlorofucofuroeckol A (pentamer), dieckol, and 8,8′-bieckol (hexamers) were at the level of 280, 650, >800, 140, 120, and 40 µM, respectively. In the case of phlorofucofuroeckol A, dieckol, and 8,8′-bieckol an inhibition type (competitive inhibition) and the inhibition constant (*K_i_*) values (130, 115, and 35 µM, respectively) were also established. Additionally, it was confirmed that the higher molecular weight of inhibitor the stronger inhibition is observed. This is probably associated with a stronger effect on the dimensional structure of the enzyme^55^.

Procyanidin B1, procyanidin B3, epicatechin, and catechin exhibited a comparable inhibitory activity to disodium cromoglycate (51.1%) at the concentration of 250 µM (50.1, 28.9, 45.9, and 29.9%, respectively). At the concentration of 125 µM, epicatechin (30.3%), and procyanidin B1 (33.5%) showed a higher activity than DSCG (22.9%).

### 4.3. Non-polyphenols as inhibitors of hyaluronidase: structure–activity relationships (SARS)

#### 4.3.1. Alkaloids

Natural alkaline nitrogen compounds are synthesised by plants, fungi, bacteria, and animals. Alkaloids, in minimal doses, have a strong physiological effect, especially on the central nervous system. Moreover, these compounds have antitumor, anaesthetic, antifungal, antibacterial, anti-inflammatory, and analgesic properties. Due to the heterocyclic ring system, we distinguish derivatives: pyridine and piperidine, tropane, quinoline, quinoline, indole, ergot, and purine^56^. A study by Girish et al. determined the effect of aristolochic acid on the activity of purified Indian cobra venom hyaluronidase (NNH1) and the activity of whole venom Hyal. The tested compound inhibited NNH1 non-competitively. Besides, the venom’s administration with aristolochic acid to mice showed more than a twofold increase in survival time compared to mice injected with the venom alone. Lower survival was obtained by splitting the application of the inhibitor over time (10 min). Aristolochic acid did not bind to the enzyme’s active site but interacted with exposed tyrosine and tryptophan HAase residues. Aristolochic acid (50, 100, and 200 µM) inhibited NNH1 at the level of 100% for each concentration. Other alkaloids, ajmaline, and reserpine inhibited Hyal weaker than aristolochic acid (ajmaline 11, 26, 40%; reserpine 9, 23, 31%, respectively) (Table 3)^57^.

**Figure 7.**
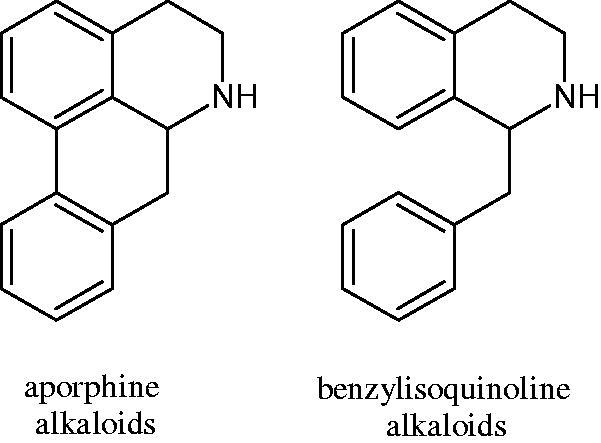
Structure of benzylisoquinoline alkaloids and apomorphine alkaloids.

Another study^58^ determined the effect of alkaloids isolated from the methanolic extract of *Nelumbo nucifera* Gaertn. flowers harvested at different stages of bloom (beginning of bloom, one-third in bloom, half in bloom, three-quarters in bloom, and full bloom). Samples flowering at half (52.69 mg per dried flower) had the highest alkaloid content (Figure 7). Among the alkaloids, nornuciferin (${\mathrm{IC}}_{50}$= 22.5 µM), asymilobin (${\mathrm{IC}}_{50}$= 11.7 µM), norarmepavin (${\mathrm{IC}}_{50}$ = 26.4 µM), coclaurin (${\mathrm{IC}}_{50}$= 11.4 µM), and norjuzyfin (${\mathrm{IC}}_{50}$= 24.3 µM) inhibited Hyal. The activity of alkaloids was more potent than that of the anti-allergic drug disodium cromoglycate (${\mathrm{IC}}_{50}$= 64.8 µM). The N-methyl group reduces the ability of alkaloids to inhibit Hyal, e.g. nuciferine ${\mathrm{IC}}_{50}$ > 100 µM < nornuciferine ${\mathrm{IC}}_{50}$= 22. µM or asimilobine ${\mathrm{IC}}_{50}$= 11.7 µM > N-methylasimilobine. On the other hand, demethylation of the hydroxyl groups increases their activity (asimilobine ${\mathrm{IC}}_{50}$= 11.7 µM > nornuciferine ${\mathrm{IC}}_{50}$= 22.5 µM). The observed structural relationships apply to both benzylisoquinoline alkaloids and apomorphine alkaloids (Tables 6 and 7).

**Table 6.** Structures of the active alkaloids towards hyaluronidase.

Structure	Compound
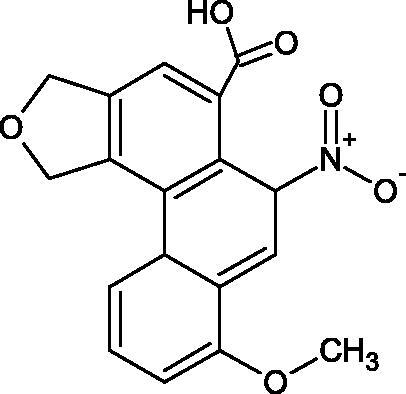	Aristolochic acid
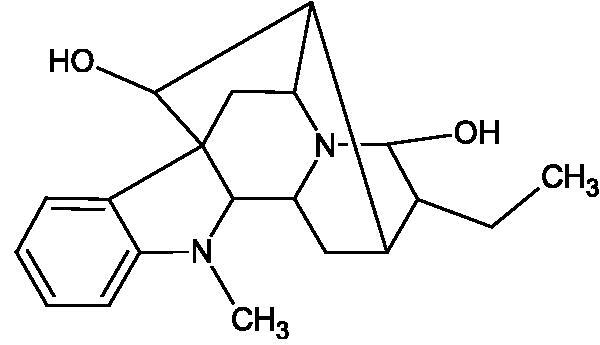	Ajmaline
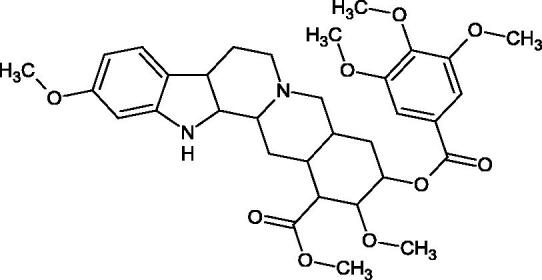	Rezerpine
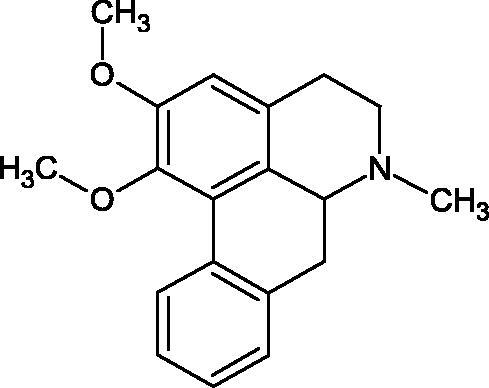	Nuciferine
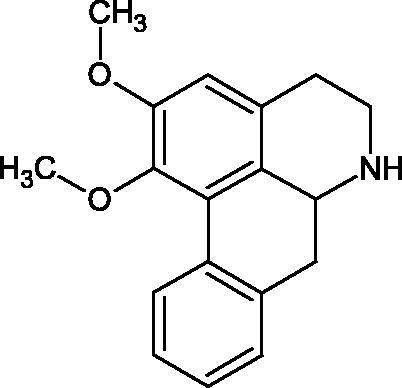	Nornuciferine
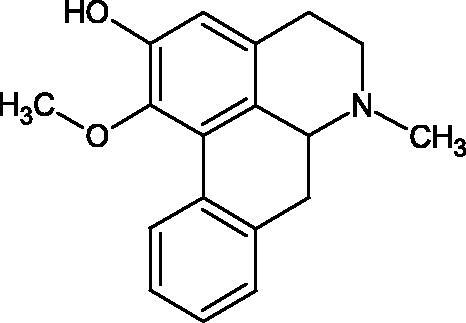	N-methylasimilobine
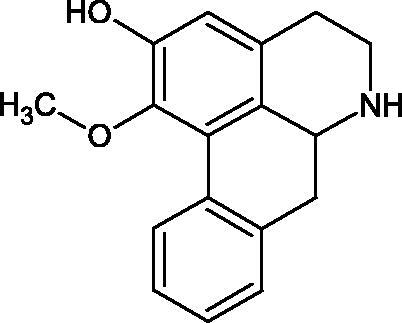	Asimilobine
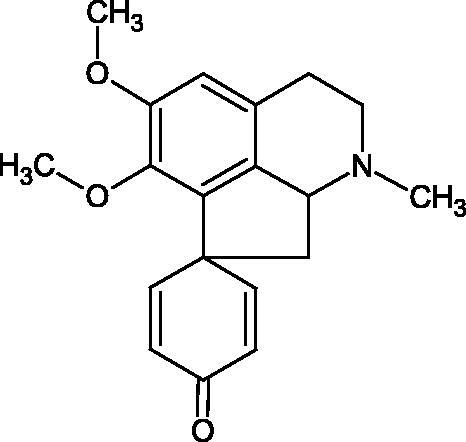	Pronuciferine
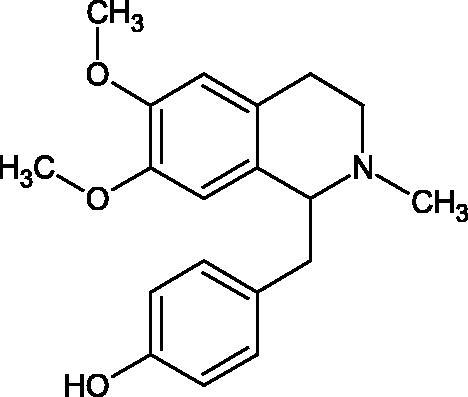	Armepavine
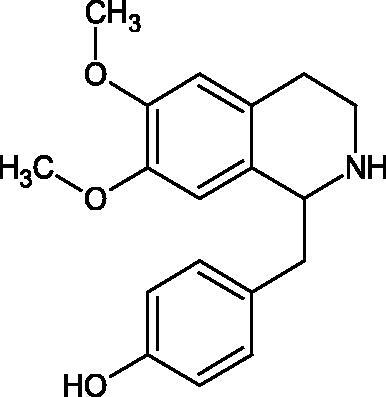	Norarmepavine
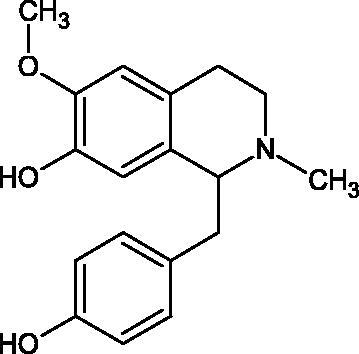	N-methylcoclaurine
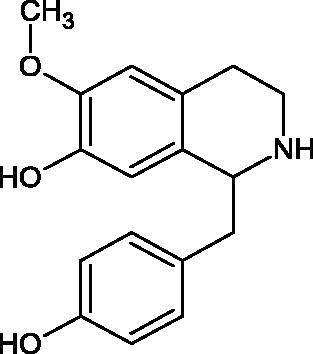	Coclaurine
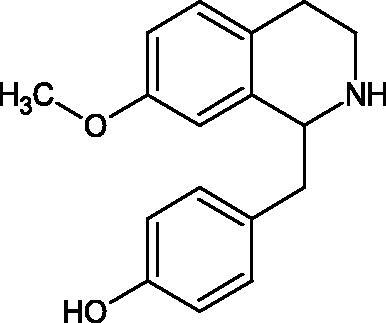	Norjuziphine

**Table 7.** Activity of benzylisoquinoline alkaloids and apomorphine alkaloids against hyaluronidase.

Compound	The half-maximal inhibitory concentration ${\mathrm{IC}}_{50}$ (µM)	Type of enzyme	Unit of enzyme activity (unit/mL)	References
Nuciferine	>100 μM	Type IV-S from bovine testes	340	Morikawa et al.^58^
Nornuciferine	22.5			
N-methylasimilobine	–			
Asimilobine	11.7			
Pronuciferine	–			
Armepavine	>100 μM			
Norarmepavine	26.4			
N-methylcoclaurine	>100 μM			
Coclaurine	11.4			
Norjuziphine	24.3			

#### 4.3.2. L-ascorbic acid

Ascorbic acid (vitamin C – AA) is a compound commonly found in the world of plants and animals. Human is incapable of synthesising vitamin C, therefore, it must be supplied in the diet (parsley, red pepper, black currant, and Brussels sprouts). The ability of vitamin C to create an oxidative system (AA < => AA < => dehydroascorbic acid) determines its antioxidant properties. AA is the most important antioxidant of extracellular fluids in the human body. It is present in high concentrations in the eyeball and lymphocytes, protecting cells against reactive forms of oxygen and nitrogen. Besides its antioxidant activity, AA is involved in the absorption of non-heme iron; in the metabolism of fats, cholesterol, and bile; regeneration of vitamin E in the cell membrane; in the synthesis of collagen, accelerating the wound healing process. Additionally, the presence of AA in the skin may constitute a defense mechanism against an invasion of pathogenic bacteria. Despite the fact that AA is one of the most known biologically-active compound its anti-Hyal activity and SAR are still unknown in details. Several studies involving AA and AA derivatives have found to be able to inhibit Hyals^59–61^.

In 2001, Li et al. first described a competitive type of AA inhibition on Hyal isolated from *Streptococcus pneumoniae* LHyal (hyaluronan lyase). The activity of AA towards Hyal is due to the structural similarity of vitamin C to glucuronic acid, being one of the basic building blocks of hyaluronan (HA) (β-1,4-glucuron-β-1,3-glucosamine). It was found that one AA molecule may bind to the enzyme’s active site. The AA carboxyl group provides a negative charge that directs the molecule to the positively charged enzyme gap. In the active centre of the enzyme, AA interacts with amino acids through hydrophobic (Trp-292), ionic (Arg-243, Arg-462, and Arg-466), and hydrogen (Tyr-408, Asn-290, and Asn-580) bonds^62^.

In 2003, Okorukwu et al. confirmed the inhibitory effect of AA and AA analogs on the activity of bovine testicular Hyal (BTH – final concentration 3.5 Units/mL) and LHyal (*Streptococcus zooepidemicus –* final concentration 2.5 Units/mL). Gel permeation chromatography (GPC) was used in this study to evaluate the inhibitory activity. The AA and AA derivatives (D-iso-ascorbic acid and dehydroascorbic acid) blocked the hyaluronan lyase more strongly than BTH. D-Saccharic-1,4-lactone and saccharic acid inhibited LHyal without affecting the enzymatic activity of testicular Hyal. The introduction of a carboxyl group that gives the molecule a negative charge positively affects the inhibitory effect of AA derivatives. Hydrogenation of the double bond between the 2nd and 3rd carbon atoms decreases the activity of the compounds. Saccharic acid can be used to develop selective inhibitors of bacterial hyaluronan lyase (Table 8)^63^.

**Table 8.** Structures of L-ascorbic acid and its derivatives with an anti-hyaluronidase activity.

Name	Structure
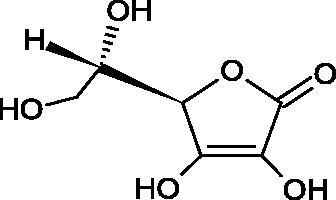	L-Ascorbic acid
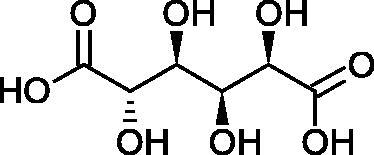	Saccharic acid
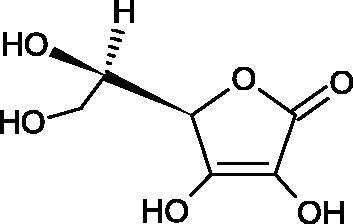	D-isoascorbic acid
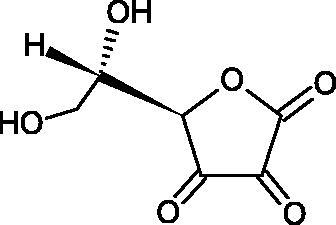	Dehydroascorbic acid
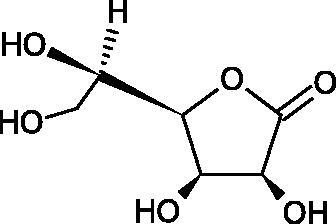	L-gulonic-γ-lactone
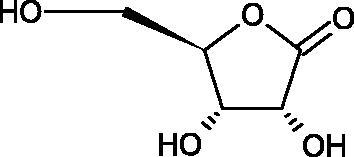	D-ribonic-γ-lactone
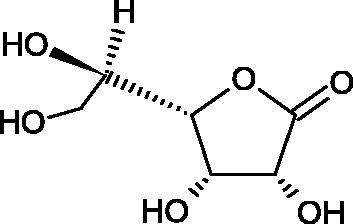	D-gulonic-γ-lactone
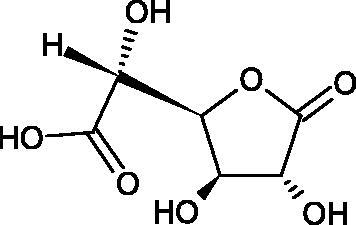	D-Saccharici-1,4-lactone
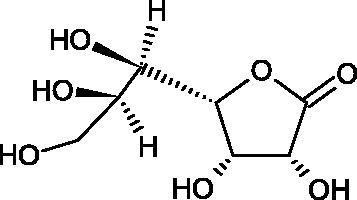	α-D-glucoheptonic-γ-lactone
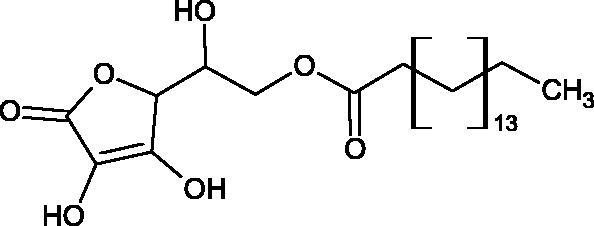	L-Ascorbyl palmitate

In a study conducted by Botzki et al., a positive correlation was confirmed between the inhibition of Hyal activity and the increased hydrophobic interactions. L-Ascorbyl palmitate, through an increase in hydrophobic interactions with Phe343, His399, and Thr400 in the active centre, led to increased inhibition of hyaluronan lyase (competitive inhibition). A similar effect was achieved with BTH. The long alkyl chain interacts with a hydrophobic channel formed primarily by the amino acids Ala-84, Leu-91, Tyr-93, Tyr-220, and Leu-344^64^.

The new LHyal inhibitors should have a larger ring system to favourably influence the hydrophobic bonding to the Trp-292 indole group and contain at least one negative charge group (carboxyl group), which brings the inhibitor to the cleft region (rich in positively charged arginine).

**Figure 8.**
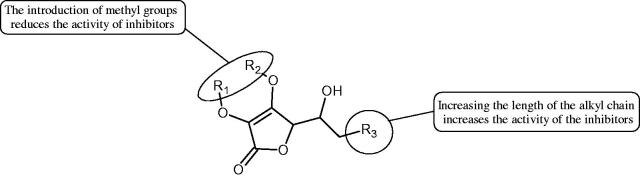
Chemical groups of L-ascorbic acid involved in the inhibition of hyaluronidase.

Spickenreither et al. examined the effect of 6-O-acylated AA derivatives on the Hyal activity of BTH and *Streptococcus agalactiae* strain 4755 (Sag Hyal 4755). All compounds showed more potent activity against bacterial lyase. Methylation of the endiol system reduces the activity of vitamin C analogs. On the other hand, 2 and 3 dibenzylated derivatives showed more potent inhibitory properties than AA. The increase in potency is due to additional hydrophobic interactions between the rings and the active centre. An increase in the length of the 6-O-acyl residue (13 b-j) results in increased inhibitory activity. The IC_50_ for octadecanoate was 0.9 and 39 µM for BTH and Sag Hyal 4755, respectively. Shortening of the aliphatic chain and adding phenyl, p-phenylene, or p-biphenyl groups leads to compounds with comparable inhibitory properties. Additionally, ether bonds in the synthesis of new inhibitors positively influence their activity. This is associated with the formation of additional hydrogen bonds in the active centre (Figure 8; Table 9)^65^.

**Figure 9.**
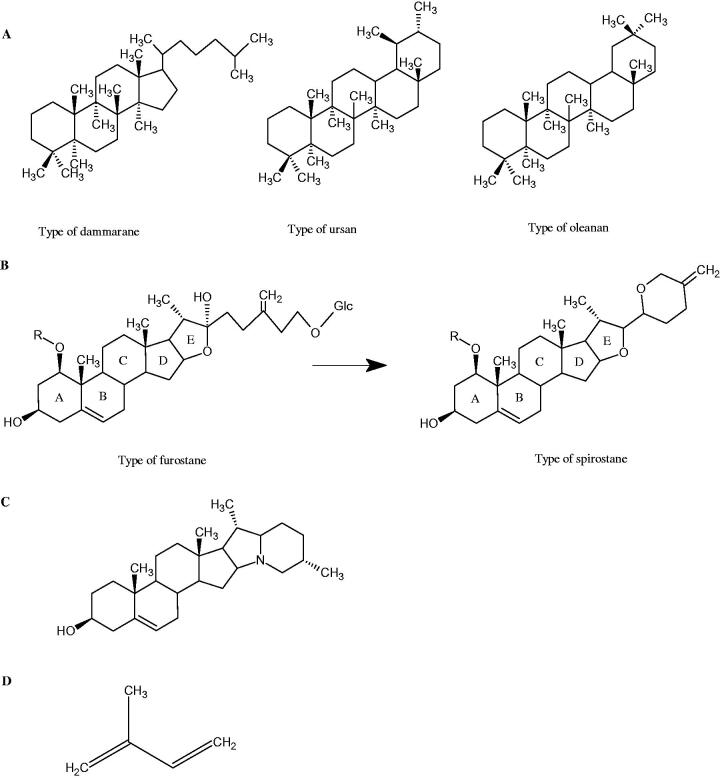
Chemical structures of saponins. A – triterpene saponins, B – steroid saponins, C – steroid alkaloids, and D – isoprene.

**Table 9.** Structures and activity of the vitamin C derivatives against hyaluronidase.^65^

	Compound	Substituent			${IC}_{50}$ (µM) or % inhibitiona, pH 5.0	
		$R_{1}$	$R_{2}$	$R_{3}$	SagHyal4755	BTH
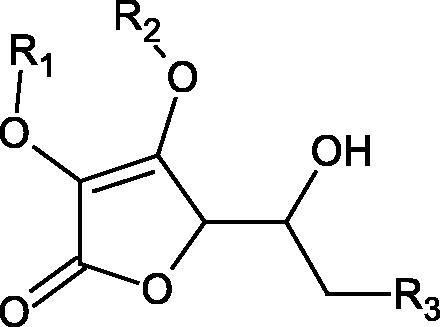	1	H	H	H	6100	Inactive
	6	Me	Me	H	Inactive	Inactive
	7	Bn	Bn	H	355	Inactive
	8	${\mathrm{CH}}_{2}{\mathrm{CH}}_{2}$		H	24% (2000)	Inactive
	9	Me	Me	$\mathrm{CO}({{\mathrm{CH}}_{2})}_{14}{\mathrm{CH}}_{3}$	5% (160)	Inactive
	10	Bn	Bn	$\mathrm{CO}({{\mathrm{CH}}_{2})}_{14}{\mathrm{CH}}_{3}$	Inactive	Inactive
	11	${\mathrm{CH}}_{2}{\mathrm{CH}}_{2}$		$\mathrm{CO}({{\mathrm{CH}}_{2})}_{14}{\mathrm{CH}}_{3}$	32% (190)	Inactive
	13a	H	H	$\mathrm{COC}({{\mathrm{CH}}_{3})}_{3}$	43% (1100)	Inactive
	13b	H	H	$\mathrm{CO}({{\mathrm{CH}}_{2})}_{4}{\mathrm{CH}}_{3}$	475	Inactive
	13c	H	H	$\mathrm{CO}({{\mathrm{CH}}_{2})}_{6}{\mathrm{CH}}_{3}$	772	Inactive
	13d	H	H	$\mathrm{CO}({{\mathrm{CH}}_{2})}_{8}{\mathrm{CH}}_{3}$	102	1380
	13e	H	H	$\mathrm{CO}({{\mathrm{CH}}_{2})}_{9}{\mathrm{CH}}_{3}$	72	580
	13f	H	H	$\mathrm{CO}({{\mathrm{CH}}_{2})}_{10}{\mathrm{CH}}_{3}$	47	208
	13g	H	H	$\mathrm{CO}({{\mathrm{CH}}_{2})}_{11}{\mathrm{CH}}_{3}$	14.3	96
	13h	H	H	$\mathrm{CO}({{\mathrm{CH}}_{2})}_{12}{\mathrm{CH}}_{3}$	8.4	71
	13i	H	H	$\mathrm{CO}({{\mathrm{CH}}_{2})}_{14}{\mathrm{CH}}_{3}$	4.2	57
	13j	H	H	$\mathrm{CO}({{\mathrm{CH}}_{2})}_{16}{\mathrm{CH}}_{3}$	0.9	39
	13k	H	H	CO−Ph	132	33% (1430)
	13l	H	H	$\mathrm{CO}{\mathrm{CH}}_{2}-pC_{6}H_{4}-\mathrm{Ph}$	358	2006
	13m	H	H	$\mathrm{CO}({{\mathrm{CH}}_{2})}_{5}O-\mathrm{Ph}$	717	Inactive
	13n	H	H	$\mathrm{CO}({{\mathrm{CH}}_{2})}_{5}O{\mathrm{CH}}_{2}Ph$	437	Inactive
	13o	H	H	$\mathrm{CO}({{\mathrm{CH}}_{2})}_{5}O-p-C_{6}H_{4}-\mathrm{Ph}$	61	188
	13p	H	H	$\mathrm{CO}({{\mathrm{CH}}_{2})}_{5}O{\mathrm{CH}}_{2}-pC_{6}H_{4}-\mathrm{Ph}$	102	543
	13q	H	H	$\mathrm{CO}({{\mathrm{CH}}_{2})}_{5}O-pC_{6}H_{4}-C_{2}H_{5}$	280	Inactive
	13r	H	H	$\mathrm{CO}({{\mathrm{CH}}_{2})}_{5}O-pC_{6}H_{4}-O{\mathrm{CH}}_{2}-\mathrm{Ph}$	76	210
	13s	H	H	$\mathrm{CO}({{\mathrm{CH}}_{2})}_{10}O-\mathrm{Ph}$	31	105
	13t	H	H	$\mathrm{CO}({{\mathrm{CH}}_{2})}_{10}pC_{6}H_{4}-\mathrm{Ph}$	7.5	37

Bn: benzyl group.

#### 4.3.3. Glycosides

Glycosides are a group of the organic compounds consisting of sugar and an aglycone part. The bond between the sugar and the aglycone is called a glycosidic bond. The chemical nature of aglycones is very different, they can be alcohols, lactones, phenolic acids, thiols, etc. The sugar portion may consist of 1–12 monosaccharide, disaccharide, or oligosaccharide molecules. The glycosides widely occur in the plant world, especially in higher plants. Because of the chemical diversity of glycoside, the plant-based sources of glycosides are important in phytotherapy^66^.

##### 4.3.3.1. Cyanogenic glycosides

Cyanogenic glycosides are one of the glycosides groups that have an inhibitory effect on Hyal. In a study by Tanyildizi et al., it was checked how different doses of linamarine and amygdalin affect Hyal activity, motility, and morphology of bull sperm obtained from the Holstein bulls aged 2–3 years (Table 10). The samples were divided into 5 equal parts and mixed with linamarine at doses of 0.5, 0.75, 1, and 2 µM and with amygdalin at doses of 0.4, 0.8, 1, and 2 µM. Incubation of compounds with sperm resulted in a significant reduction (dose-dependent) of sperm motility and Hyal activity compared to the control group (isotonic saline solution). Both linamarine and amygdalin did not change sperm morphology. The authors concluded that the fertilisation ability of bull sperm could be inhibited by the over-consumption of plants rich in cyanogenic glycosides^67^.

**Table 10.** Structures of the active cyanogenic glycosides towards hyaluronidase.

Structure	Compound
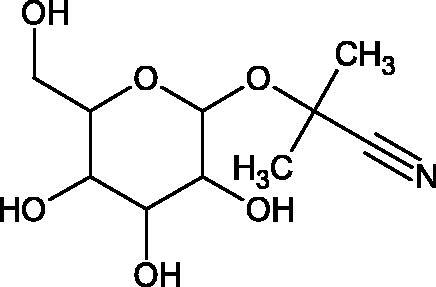	Linamarine
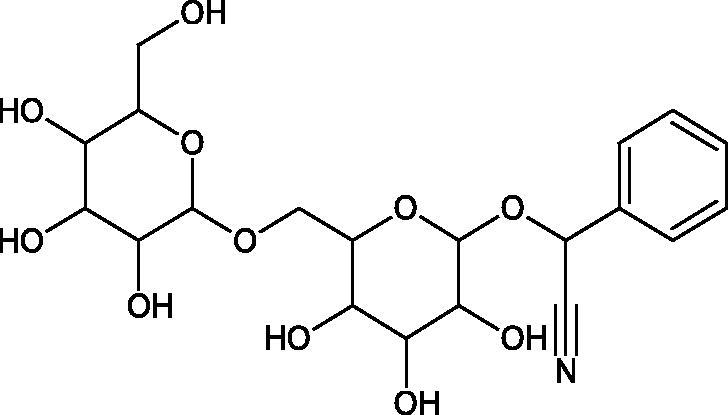	Amygdalin

#### 4.3.4. Terpene/terpenoids

Terpenes are composed of a varying number of isoprene units (Figure 9). They are commonly found in the plant world in hydrocarbons or oxidised forms (with hydroxyl, carbonyl, or carboxyl groups). Depending on the number of isoprene residues, we distinguish monoterpenes (C10), diterpenes (C-20), sesquiterpenes (C-15), triterpenes (C-20), and tetraterpenes (C-40). Due to their highly diverse chemical structure, terpenes exhibit a variety of biological activities, such as antibacterial, antiviral, anticancer, anti-inflammatory, and sedative effects^68^.

##### 4.3.4.1. Monoterpene/monoterpenoids

Morikawa et al. investigated the effect of methanolic extract of rhizome of *Picrorhiza kurroa* Royle ex Benth. on Hyal activity (Type IV-S from bovine testes). Seven new acylated iridoid glycosides (picrorhizaosides A–G) and six known iridoid glycosides were isolated from the extract. Among the isolates, picrorhizaosides D (${\mathrm{IC}}_{50}$= 43.4 µM), picrorhizaosides E (${\mathrm{IC}}_{50}$= 35.8 µM), picrosides I (${\mathrm{IC}}_{50}$= 60.7 µM), picrosides II (${\mathrm{IC}}_{50}$= 22.3 µM), picrosides IV (${\mathrm{IC}}_{50}$= 59.2 µM), and minecoside (${\mathrm{IC}}_{50}$= 57.2 µM), showed similar or stronger Hyal inhibitory effects than the anti-allergic drugs disodium cromoglycate (${\mathrm{IC}}_{50}$= 64.8 µM), ketotifen fumarate (${\mathrm{IC}}_{50}$= 76.5 µM), and tranilast (${\mathrm{IC}}_{50}$= 227 µM), but weaker than the alkaloids isolated from Nelumbo nucifera Gaertn., such as asimilobin (${\mathrm{IC}}_{50}$= 11.7 µM) and coclaurin (${\mathrm{IC}}_{50}$= 11.4 µM)^69^.

##### 4.3.4.2. Saponin

Saponins belong to the glycosides group composed of aglycone – sapogenin (sapogenol) and glycone-sugar. Depending on the type of sapogenin we distinguish triterpene saponins, steroidal saponins, and steroidal alkaloids (Figure 9). These compounds reduce the surface tension of water solutions. They show anti-inflammatory, antibacterial, protozoal, antifungal, and antiviral activity, stimulate secretion of gastric juice, bile, and intestinal juice. They can affect cholesterol level^70^.

In a study by Zhou et al., they determined the effects of esculeoside A and its aglycone esculeogenin A on Hyal activity *in vitro* and in mice dermatitis model. Esculeoside A, a spirosolate-type glycoside, is identified as a significant component of ripe tomato fruit. The ${\mathrm{IC}}_{50}$ for esculeogenin A and esculeoside A was about 2 and 9 µM, respectively. Administration of esculeoside A at a dose of 10 mg/kg for four weeks to mice with dermatitis significantly reduced diseases symptoms^71^. Esculeoside A is a competitive inhibitor of hyalurnidase (*K_i_* = 11.0 µM)^72^. Also, the other steroid alkaloids present in tomato juice showed beneficial inhibitory effects against Hyal. Administration of 10 mg/kg esculeoside B to mice with dermatitis for four weeks significantly reduced skin inflammation. In addition, it was found that esculeoside B administration significantly inhibited T-lymphocyte proliferation and decreased IL-4 production^73^.

**Figure 10.**
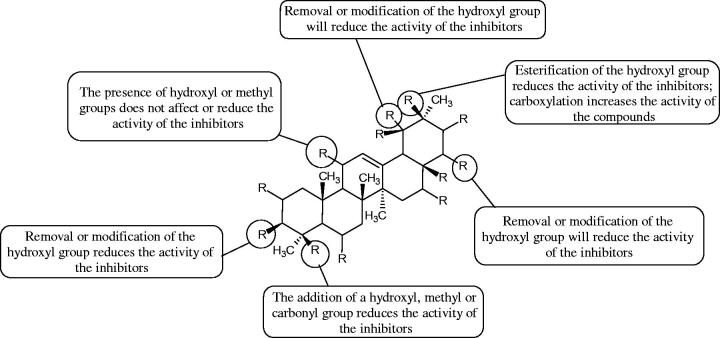
Chemical groups of triterpenic acids involved in the inhibition of hyaluronidase.

More information about the effect of saponin structure on Hyal activity was provided by QSAR studies of ursolic and oleanolic acids, the results of which exhibited the higher activity of ursolic acid than oleanolic acid. In that experiment, the effect of the position of methyl groups at 29 and 30 carbon atoms was checked. Both geminal and vicinal positions had no significant impact on an inhibitor activity. For oleanolic acid, the activity increased when the methyl group was introduced at C-17 or C-16 and decreased when the methoxyl group was introduced at C-23. The 3-OH acetylation reduced the activity of the compounds. Carboxylation of C-30 increased the activity of the compounds, while esterification of the same carbon (C-30). In addition, the introduction of a sugar moiety into the 3-OH decreased their activity. In the case of ursolic acid, the activity decreased when the hydroxyl group was modified at C-3 (3-oxo, 3-hydroxyimino, and 3-acetylate derivatives) and at C-28. Replacement of the methyl group at C-23 with a carboxyl or hydroxy methylene group decreased the activity of the compounds. As in the case of oleanolic acid, the introduction of a sugar group caused a decrease in the inhibitory activity (Figure 10; Table 11)^74^.

**Figure 11.**
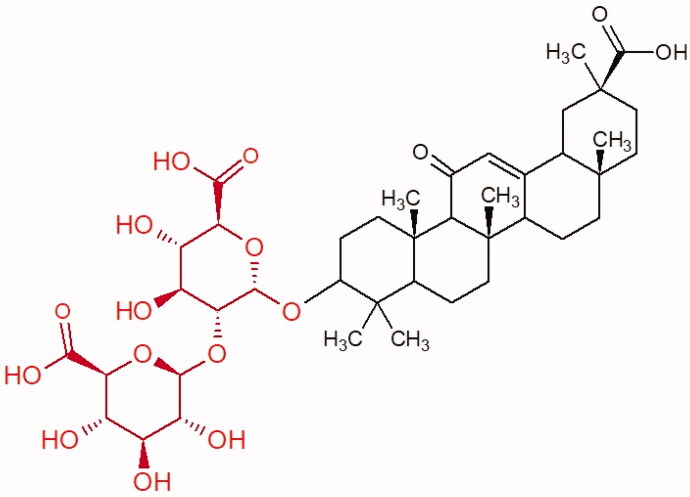
3-O-β-D-glucuronopyranoside group.

**Table 11.** Structures and an anti-hyaluronidase activity of oleanane-type saponins^73^.

	Compound	Substituent												${IC}_{50}$ (µM)
		$R_{1}$	$R_{2}$	$R_{3}$	$R_{4}$	$R_{5}$	$R_{6}$	$R_{7}$	$R_{8}$	$R_{9}$	$R_{10}$	$R_{11}$	$R_{12}$	
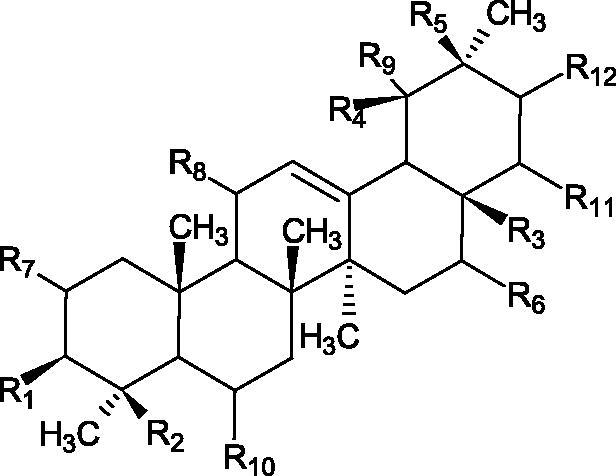 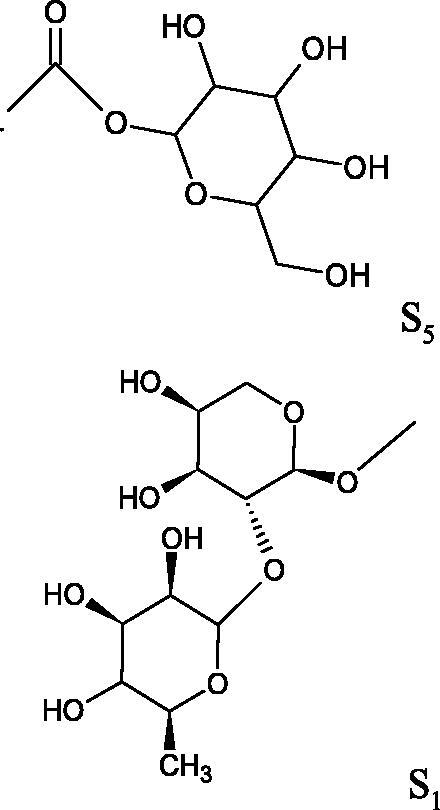 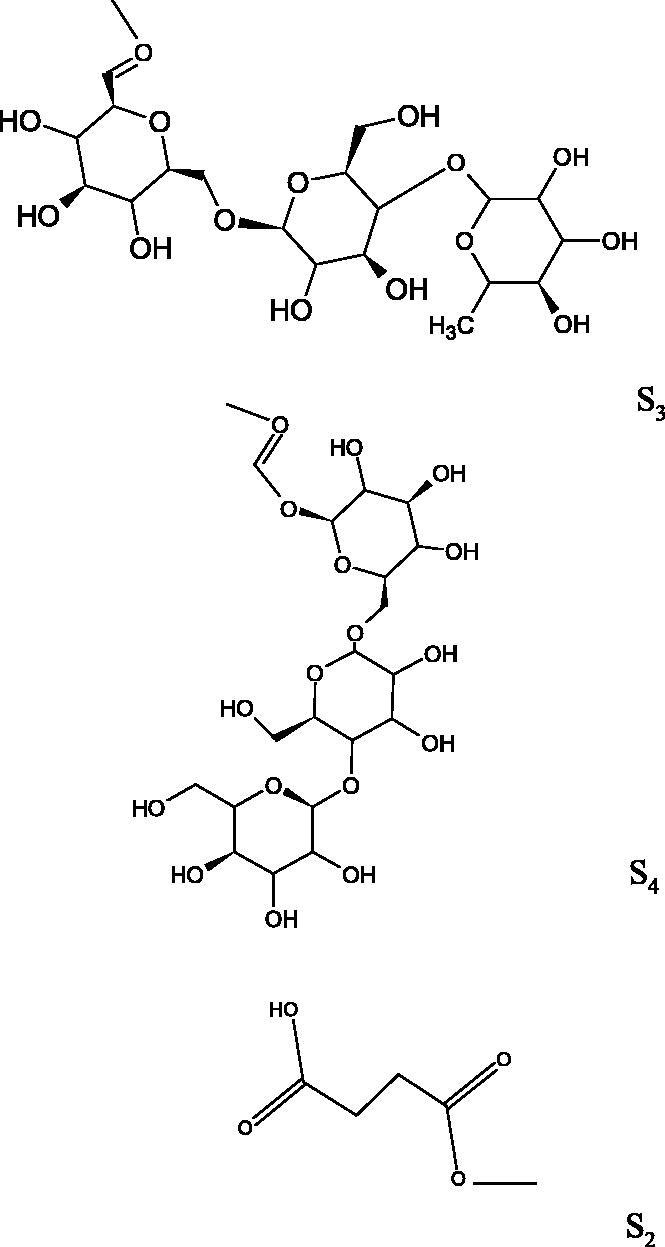	1	OH	${\mathrm{CH}}_{3}$	COOH	${\mathrm{CH}}_{3}$	H	H	H	H	H	H	H	H	103.18
	2	OH	${\mathrm{CH}}_{2}OH$	COOH	${\mathrm{CH}}_{3}$	H	H	H	H	OH	H	H	H	286.95
	3	OAc	${\mathrm{CH}}_{3}$	COOH	H	${\mathrm{CH}}_{3}$	H	H	H	H	H	H	H	1466.5
	4	=O	${\mathrm{CH}}_{3}$	COOH	${\mathrm{CH}}_{3}$	H	H	H	H	H	H	H	H	162.83
	5	NOH	${\mathrm{CH}}_{3}$	COOH	${\mathrm{CH}}_{3}$	H	H	H	H	H	H	H	H	190.94
	6	OAc	${\mathrm{CH}}_{3}$	COOH	${\mathrm{CH}}_{3}$	H	H	H	H	H	H	H	H	136.92
	7	=O	${\mathrm{CH}}_{3}$	${\mathrm{COOCH}}_{3}$	${\mathrm{CH}}_{3}$	H	H	H	H	H	H	H	H	1184.15
	8	NOH	${\mathrm{CH}}_{3}$	${\mathrm{COOCH}}_{3}$	${\mathrm{CH}}_{3}$	H	H	H	H	H	H	H	H	275.68
	9	OH	${\mathrm{CH}}_{3}$	${\mathrm{COOCH}}_{3}$	${\mathrm{CH}}_{3}$	H	H	H	H	H	H	H	H	182.51
	10	OAc	${\mathrm{CH}}_{3}$	${\mathrm{COOCH}}_{3}$	${\mathrm{CH}}_{3}$	H	H	H	H	H	H	H	H	812.93
	11	OH	${\mathrm{CH}}_{3}$	${\mathrm{CH}}_{3}$	H	H	H	H	=O	H	H	H	H	1750.91
	12	OH	${\mathrm{CH}}_{2}OH$	${\mathrm{CH}}_{3}$	${\mathrm{CH}}_{3}$	H	H	H	H	H	H	H	H	227.97
	13	OH	${\mathrm{CH}}_{3}$	${\mathrm{CH}}_{2}OH$	H	${\mathrm{CH}}_{3}$	H	H	H	H	H	H	H	206.21
	14	OH	${\mathrm{CH}}_{3}$	${\mathrm{CH}}_{3}$	${\mathrm{CH}}_{3}$	H	H	H	H	H	H	H	H	211.44
	15	OH	${\mathrm{CH}}_{3}$	COOH	H	${\mathrm{CH}}_{3}$	OH	H	H	H	H	H	H	140.91
	16	OH	${\mathrm{CH}}_{3}$	${\mathrm{COOCH}}_{3}$	H	${\mathrm{CH}}_{3}$	H	H	H	H	H	H	H	84.52
	17	$S_{1}$	${\mathrm{CH}}_{2}OH$	COOH	H	${\mathrm{CH}}_{3}$	H	H	H	H	H	H	H	842.54
	18	OH	${\mathrm{CH}}_{3}$	${\mathrm{CH}}_{3}$	H	${\mathrm{CH}}_{3}$	H	H	H	H	H	H	H	215.66
	19	OH	${\mathrm{CH}}_{2}OH$	COOH	${\mathrm{CH}}_{3}$	H	H	OH	H	H	H	H	H	115.96
	20	OH	${\mathrm{CH}}_{3}$	COOH	H	${\mathrm{CH}}_{3}$	H	H	H	H	H	H	H	227.97
	21	OH	${\mathrm{CH}}_{3}$	${\mathrm{CH}}_{3}$	H	COOH	H	H	=O	H	H	H	H	146.18
	22	$S_{2}$	${\mathrm{CH}}_{3}$	${\mathrm{CH}}_{3}$	H	COOH	H	H	=O	H	H	H	H	56.33
	23	OH	COOH	${\mathrm{CH}}_{3}$	H	${\mathrm{CH}}_{3}$	H	H	H	H	H	H	H	1482.56
	24	OH	${\mathrm{CH}}_{2}OH$	COOH	H	${\mathrm{CH}}_{3}$	H	H	H	H	H	H	H	230.00
	25	O-glucoside	${\mathrm{CH}}_{3}$	COOH	H	${\mathrm{CH}}_{3}$	OH	H	H	H	H	H	H	NA
	26	OH	COOH	${\mathrm{CH}}_{3}$	${\mathrm{CH}}_{3}$	H	H	H	H	H	H	H	H	NA
	27	OH	$S_{3}$	${\mathrm{CH}}_{2}OH$	${\mathrm{CH}}_{3}$	H	H	OH	H	H	H	H	H	NA
	28	OH	${\mathrm{CH}}_{2}OH$	${\mathrm{CH}}_{2}OH$	H	${\mathrm{CH}}_{3}$	OH	H	H	H	H	OH	OH	NA
	29	OH	${\mathrm{CH}}_{2}OH$	${\mathrm{COOS}}_{4}$	${\mathrm{CH}}_{3}$	H	H	OH	H	H	OH	H	H	NA

Similar results were obtained in another experiment aimed at an investigation of the impact of the oleanic acid structure modification on anti-Hyal activity. Oxidation of the 3-OH group in oleanic acid led to a decrease of the activity about 4-fold (59.3–15.9%). A similar effect was obtained by esterification of the COOH group (12.7% inhibition). The activity of the compounds was evaluated at a concentration of 40 µg/mL^75^.

In another study^76^, oleanane-type saponins, isoflavonoids, oxazoles, and glycosides (36 compounds) were isolated from the aerial part of *Oxytropis lanata* (Pall.) DC. The effect of saponins against Hyal (${\mathrm{IC}}_{50}$= 0.15–0.22 mM) was more potent than sodium cromoglycate, which was used as a positive control (${\mathrm{IC}}_{50}$= 0.37 mM). On the basis of the structure analysis, it was appeared that all isolated saponins contain a 3-O-β-D-glucuronopyranoside grouping in the sugar moiety, which shows a potent inhibition of Hyal, as well as the compounds with a ketone group at C-22 more strongly blocked the enzyme (Figure 11). Similar results were obtained when testing triterpene saponosides from the 3-O-β-D-glucuronopyranoside group, which blocked Hyal more strongly (2; ${\mathrm{IC}}_{50}$= 1.25 mM, 3: ${\mathrm{IC}}_{50}$= 0.68 mM, and 9: ${\mathrm{IC}}_{50}$= 0.82 mM) than rosmarinic acid used as a control (${\mathrm{IC}}_{50}$= 1.36 mM)^77^. The significance of the 3-O-β-D-glucuronopyranoside grouping was also noted by examining the triterpene saponosides camelliagenin A (IPS-1 and IPS-2) isolated from the methanolic root extract of Impatiens parviflora DC. A very interesting result has been obtained, because IPS-2 (${\mathrm{IC}}_{50}$= 286.7 µg/mL) inhibited BTH Hyal activity more strongly than escin (${\mathrm{IC}}_{50}$= 303.93 µg/mL). What is interesting, escin is recommended to be used as an anti-Hyal reference compound. The higher IC_50_ value was obtained for IPS-1; 368.1 µg/mL. Considering the structure of these compounds, a clear difference may be noticed significantly since the tested saponosides differed in their acetylation at C-16. The 16-O-acetylcameliagenin A derivative showed less activity. Therefore, the free OH group at position 16 may have a beneficial effect on Hyal inhibition, e.g. *via* participation in ions chelating in the reaction’s medium^78^.

Myose et al. demonstrated the effect of triterpene saponins isolated from methanolic extract of *Camellia sinensis* (L.) Kuntze seeds on Hyal activity. The isolated new saponin (Teaseedsaponin A-L) blocked Hyal more potently (${\mathrm{IC}}_{50}$= 19.3–55.6 µM) than rosmarinic acid (${\mathrm{IC}}_{50}$= 240.1 µM)^79^.

Another study examined how sugar moiety in saponins affects the activity of Hyals isolated from different bacterial species (*Streptococcus agalactiae* – Hyal B, *Streptomyces hyalurolyticus* – Hyal S, *Streptococcus equisimilis* – Hyal C) and bovine testes (BTH). Glycyrrhizin (1) and its aglycone glycyrrhetinic acid (2) were used as inhibitors. The tested compounds most potently blocked the activity of Hyal B [(1); ${\mathrm{IC}}_{50}$= 0.440 mM, (2); ${\mathrm{IC}}_{50}$= 0.060 mM]. For other Hyals, the action was weak [Hyal S – (1); 1.020 mM, (2); 0.260 mM; Hyal C – (1); NA, (2); NA; BTH – (1); 1.300 mM, (2); 0.090 mM). Considering the results obtained, glycyrrhetinic acid inhibited the activity of tested enzymes weaker, which may prove the significance of the sugar moiety in the inhibition of Hyal activity^25^.

Facino et al. investigated the inhibitory influence of saponins and sapogenins isolated from seeds of *Aesculus hippocastanum* L. (escin and escinol), leaves of *Hedera helix* L. (α-hederin, hederacoside C, oleanolic acid, and hederagenin), and rhizome of *Ruscus aculeatus* L. (ruscogenin). Of the *Hedera helix* L. components, only sapogenins inhibited Hyal in a dose-dependent manner. Hederagenin (${\mathrm{IC}}_{50}$= 280.4 µM) inhibited the enzyme at 100 µM (12.5%); 20.3% at 150 µM, 31% at 200 µM, 56.5% at 300 µM, and 74.2% at 500 µM (plateau). Oleanolic acid (${\mathrm{IC}}_{50}$= 300.2 µM), 29.1% at 200 µM; 48.5% at 300 µM, and 67% (plateau) at 400 µM. Glycyrrhizic acid (positive control) blocked BTH Hyal significantly less (${\mathrm{IC}}_{50}$= 550.2 µM). Hederacoside C and *α*-hederin showed no activity against Hyal. For Aesculus hippocastanurn L., escin had the highest activity (${\mathrm{IC}}_{50}$= 149.9 µM), inhibiting Hyal starting at 50 µM (4.2%); at higher concentrations of 100 (27.4%), 150 (52.0%), 200 (79.2%), and 300 µM (93.6%). Escinol was much less active (${\mathrm{IC}}_{50}$= 1.65 mM), and ruscogenin was utterly ineffective (Table 12)^80^.

**Table 12.** Structures and an anti-hyaluronidase activity of the chosen saponins.

Compounds	Name	${IC}_{50}$ mM	References
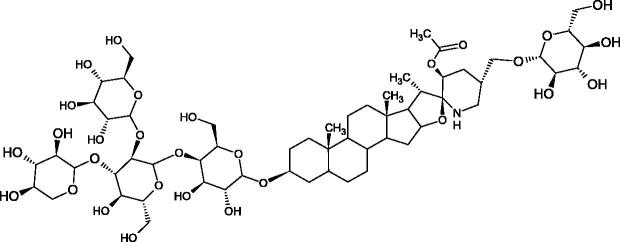	Esculeoside A	2	Zhou et al.^72^
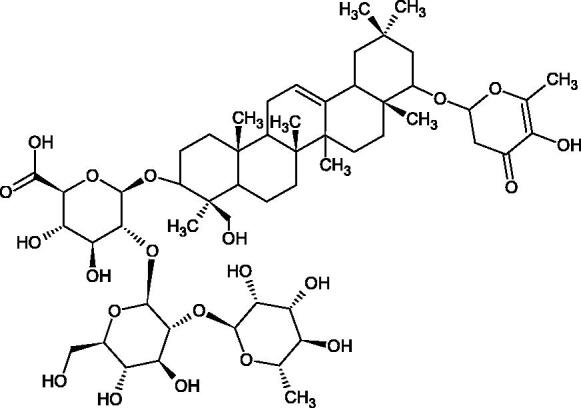	Compound 1	0.15	Buyankhishig et al.^75^
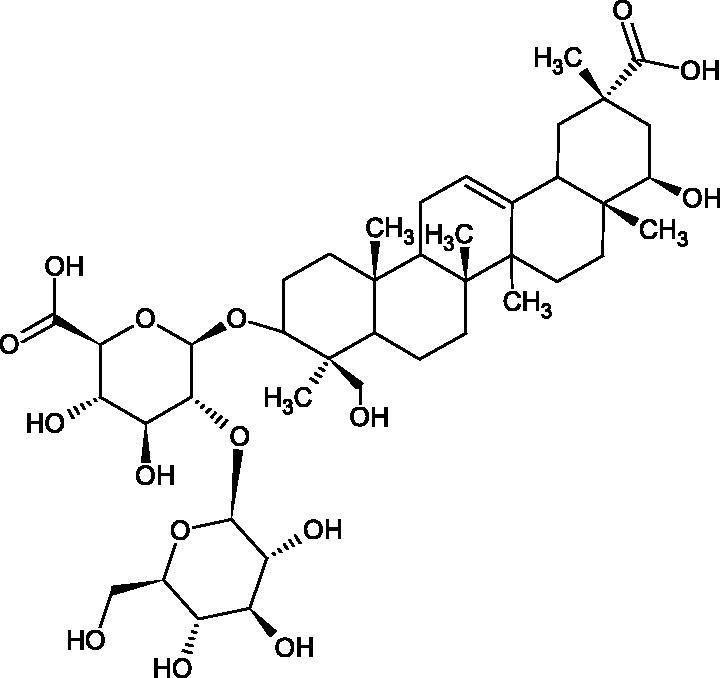	Compound 2	0.21	Buyankhishig et al.^75^
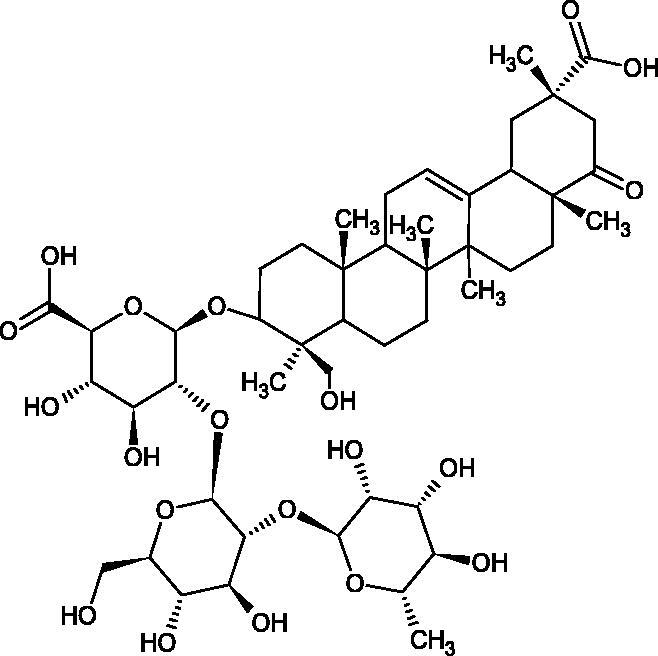	Compound 3	0.22	Buyankhishig et al.^75^
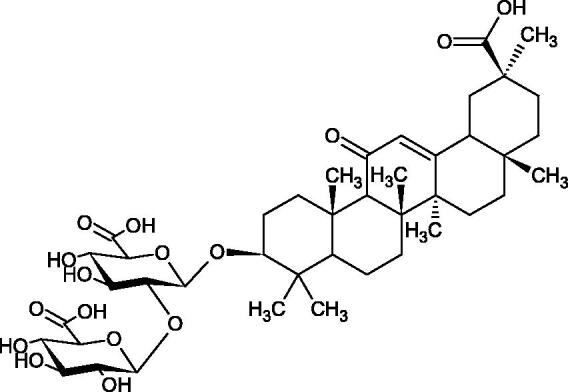	Glycyrrhizin	0.44	Hertel et al. ^37^
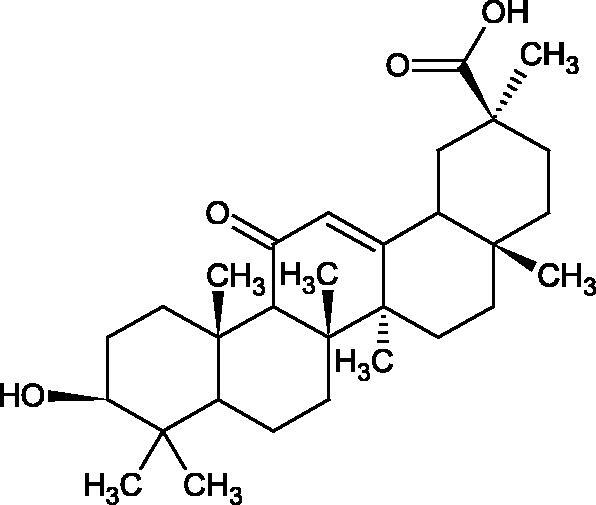	Glycyrrhetinic acid	0.06	Hertel et al. ^37^
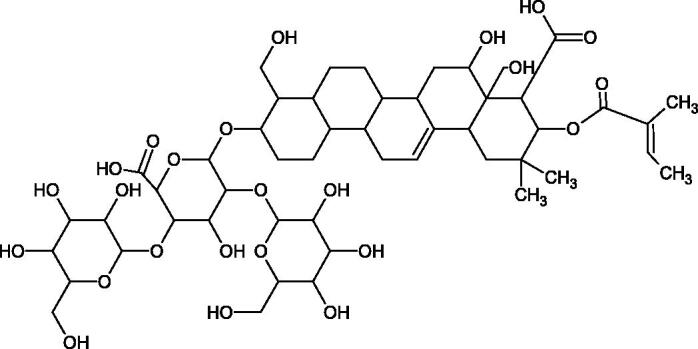	Escin	0.15	Facino et al.^79^
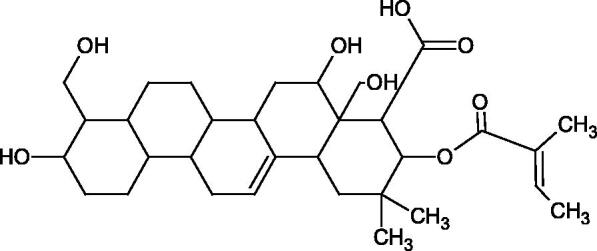	Escinol	1.65	Facino et al.^79^
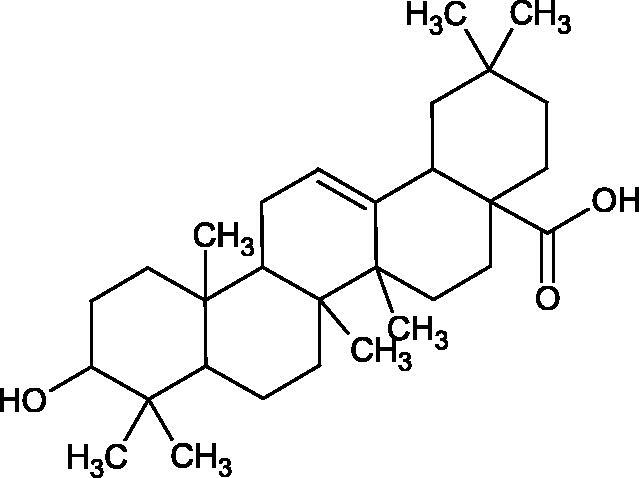	Oleanolic acid	0.300	Facino et al.^79^
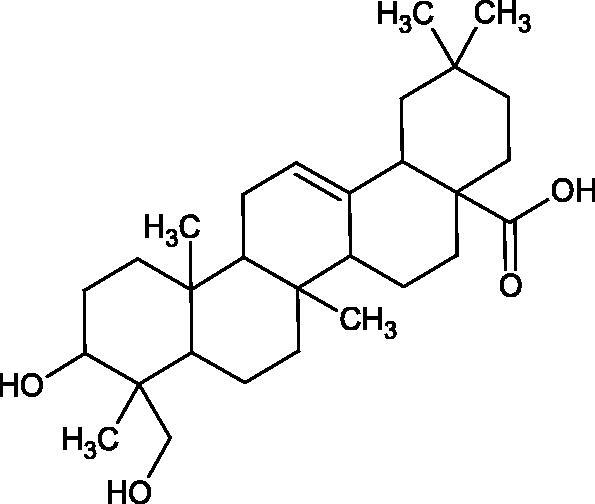	Hederagenin	0.280	Facino et al.^79^
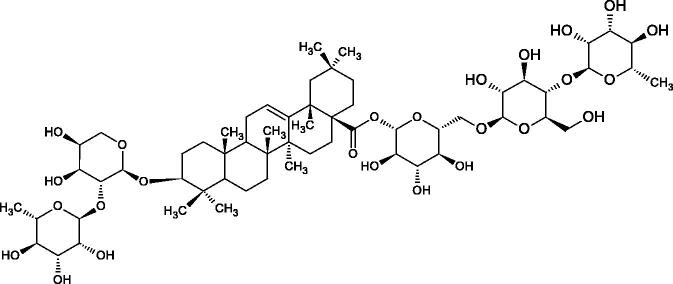	Hederacoside C	Non effect	Facino et al.^79^
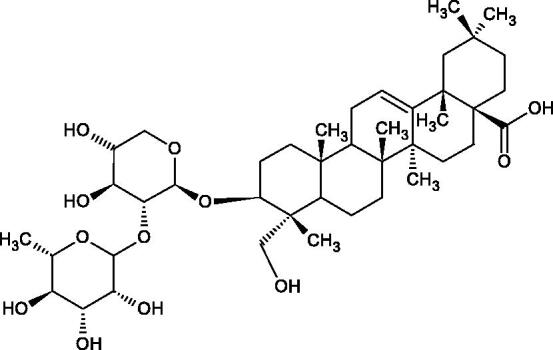	α-Hederin	Non effect	Facino et al.^79^
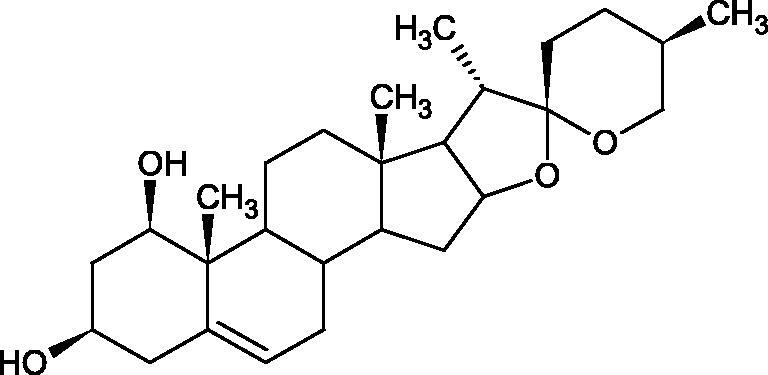	Ruscogenin	Non effect	Facino et al.^79^
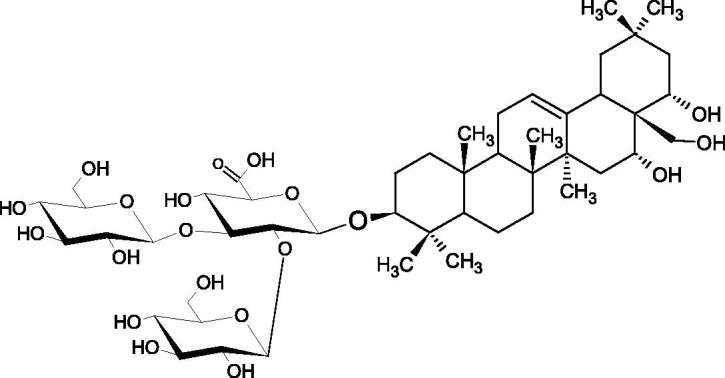	Glycoside camelliagenin A	303.93 µg/mL	Grabowska et al.^77^
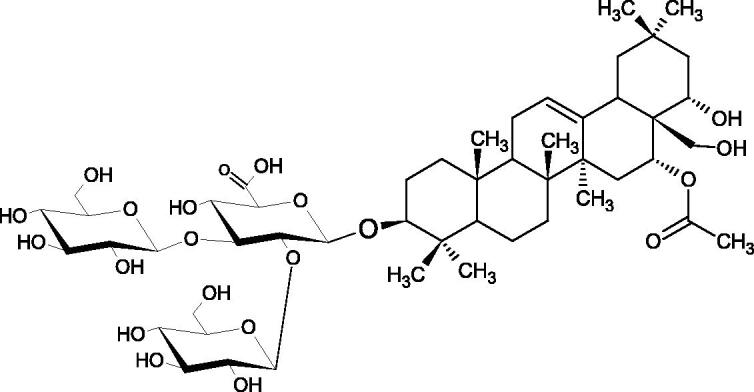	Glycoside 16-O- acetylcamelliagenin A	286.7 µg/mL	Grabowska et al.^77^

The olean-type saponins, spinasaponin A (lack of activity), spinasaponin A 28-O-glucoside (IC_50_ = 620 µM), udosaponin B (IC_50_ = 750 µM), and sandrosaponin IX (IC_50_ = 370 µM), isolated from the roots of *Oenanthe javanica*, showed moderate ability to inhibit Hyal. Esterification of the 28-COOH group with *β*-D-glucopranosyl increased the activity of the compounds^81^.

## 5. Inhibitors of tyrosinase

### 5.1. Tyrosine and tyrosinase

**Figure 12.**
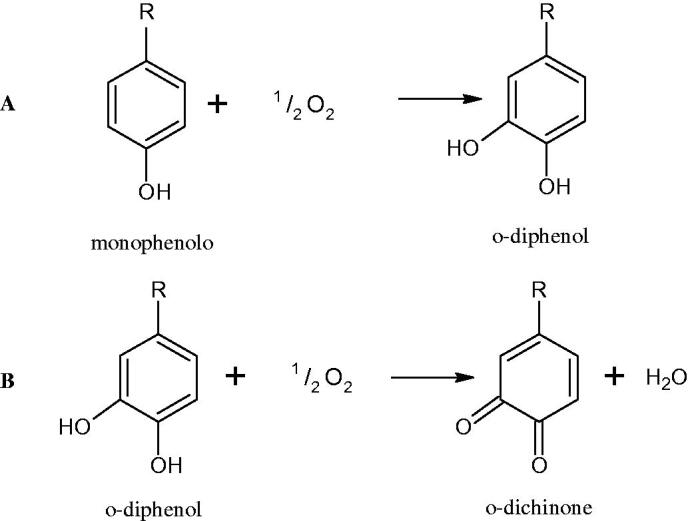
A – reaction hydroxylation of monophenols to *o*-diphenols; reaction B – oxidation of *o*-diphenols to *o*-quinones.

**Figure 13.**
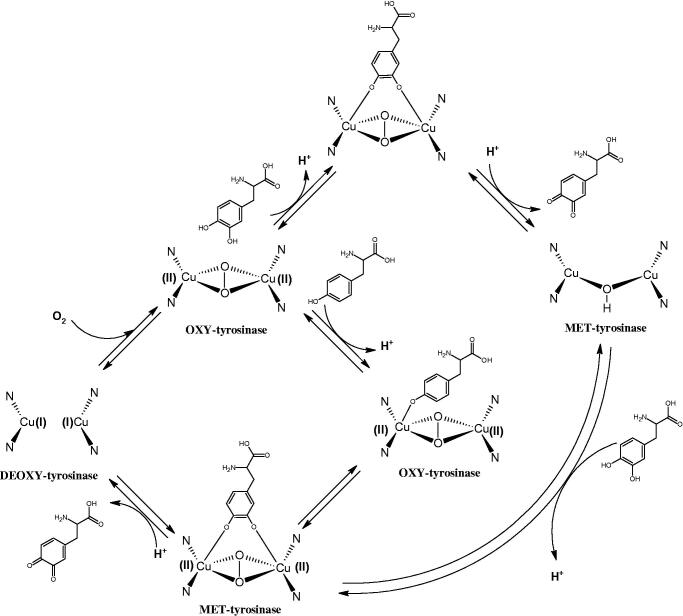
Mechanism of the tyrosinase action as monophenolase and diphenolase.

Tyrosinase is a key enzyme involved in melanogenesis. This enzyme belongs to the class of oxidoreductases (EC 1.14.18.1). It is responsible for the catalysis of tyrosine hydroxylation to L-DOPA and the oxidative conversion of L-DOPA to dopaquinone (Figure 12). The active centre of tyrosinase consists of two copper atoms linked by a coordination bond with three histidine residues. Three different types of tyrosinase are involved in melanin production: oxy-tyrosinase, met-tyrosinase, and deoxy-tyrosinase. Oxy-tyrosinase and met-tyrosinase have Cu (II) copper atoms in their active centre, and deoxy-tyrosinase has two Cu (I) atoms. Deoxy-tyrosinase does not perform a catalytic function, but it is easily converted into the oxy form, which is the only form of the enzyme capable of transforming both monophenol and diphenol substrates. On the other hand, met-tyrosinase is formed during the reaction catalysed by the oxy form and is responsible only for reactions with diphenolic substrates (Figure 13)^81–84^.

**Figure 14.**
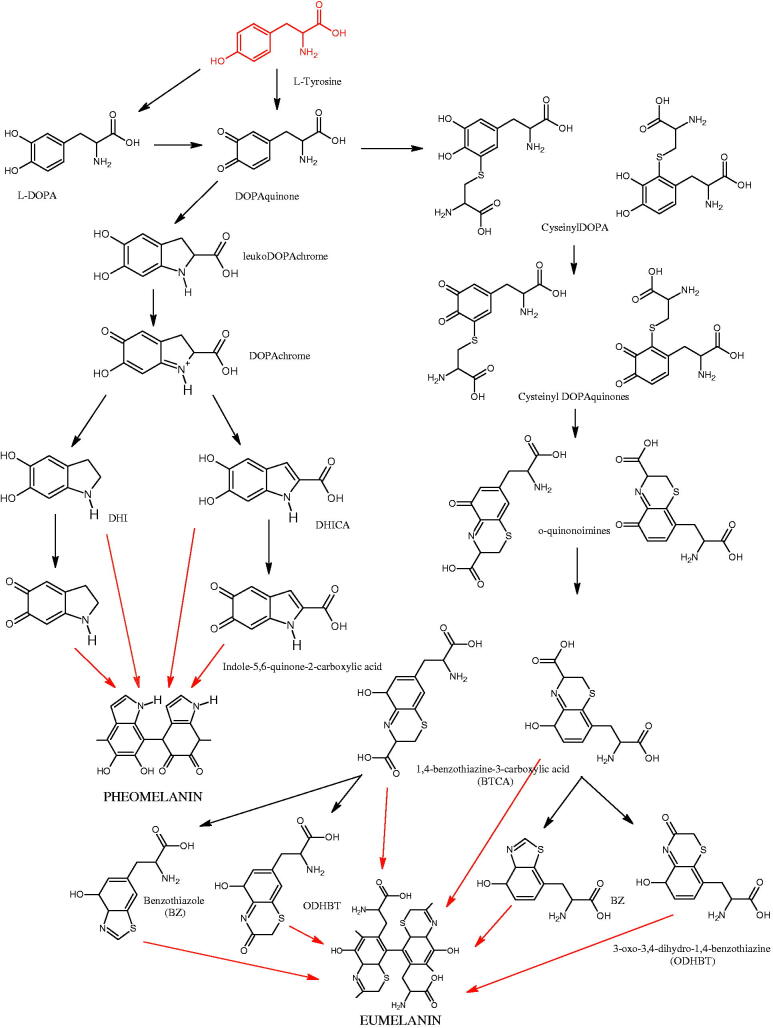
The pathway of melanin synthesis.

Tyrosinase is an enzyme widely distributed in nature, including in fungi and to a lesser extent in bacteria and algae. This enzyme is also found in plants and animals. In higher vertebrates, melatonin is produced in melanocytes present in the epidermis, hair follicles, the uveal membrane of the eye (choroid, ciliary body, and iris), the inner ear (cochlea), and the central nervous system (the arachnoid and internal filum terminale). Melanins are macromolecular polymeric pigments resulting from the oxidation and polymerisation of phenolic compounds (Figure 14). Melanin synthesis takes place in vesicles called melanosomes and it is considered to be one of the most common pigments in nature. In mammals, melanosomal tyrosinase is involved in the formation of black-brown eumelanin and yellow-reddish phaeomelanin. Eumelanin has photoprotective properties, resulting from the ability to absorb ultraviolet radiation (UV) and neutralise free radicals and reactive oxygen species (ROS). On the other hand, pheomelanin has photosensitising properties and, under the influence of UV radiation, can participate in the generation of ROS. In mammals, melanin pigmentation performs many critical physiological tasks, such as adaptive colouration, protection of essential tissues against UV radiation, thermal control of the organism, regulation of vitamin D 3 biosynthesis. Abnormal tyrosinase activity is responsible for skin abnormalities such as vitiligo or freckles. Also, tyrosinase may play a role in carcinogenesis and neurodegenerative diseases such as Parkinson’s disease. Tyrosinase also contributes to the formation of brown colour in fruits and vegetables due to the reaction of dopaquinone with amino acids and proteins present in these foods. In most studies on the inhibition of tyrosinase activity, fungal tyrosinase was used due to its widespread availability. The enzyme isolated from the mushroom *A. bisporus* is very similar in structure to tyrosinase occurring in mammals, making it a suitable model for studying the process of melanogenesis. Since tyrosinase is a reasonably significant target in agriculture, food, medicine, and cosmetology, much attention has been paid to the development and screening of tyrosinase inhibitors^85–87^.

### 5.2. Polyphenols as inhibitors of tyrosinase: a structure–activity relationship

The positive effect of polyphenols on human health is mainly related to their antioxidant properties. The antioxidant activity of individual polyphenols depends on the number of hydroxyl groups and their location. It has been shown that the more hydroxyl groups in a molecule, the more potent antioxidant activity. Compounds with redox properties effectively prevent melanin biosynthesis due to their multidirectional mechanism of action. Tyrosinase inhibition by polyphenols is based on free radical scavenging properties and the ability to chelate copper in the tyrosinase active site.

#### 5.2.1. Phenolic acids

Phenolic acids are one of the most common groups of organic compounds present in plants. They are made of a phenolic ring and a carboxylic acid residue. There are two subclasses of phenolic acids: derivatives of benzoic acid and cinnamic acid. Several studies have shown their inhibitory effect on tyrosinase activity and that activity was related to the number and position of hydroxyl groups^88^. Considering the structure of phenolic acids, three mechanisms of tyrosinase inhibition can be distinguished. The first is related to the chelation of copper ions in the active centre. The second mechanism is associated with the disturbance of the enzyme’s tertiary structure through hydrogen bond formation. The third mechanism involves constructing hydrogen bonds between the hydroxyl groups of phenolic acids and the carbonyl oxygen of the Tyr98 ORF378 protein, preventing the interaction between tyrosinase and ORF378. Consequently, ORF378 cannot serve as a Cu (II) ion transporter to the enzyme’s active site^89^^,^^90^.

##### 5.2.1.1. Hydroxybenzoic acids

Kubo et al. investigated the effect of anisic acid and its derivatives on L-DOPA oxidation by tyrosinase. As the concentration of anisic acid increased, the enzymatic activity decreased sharply but was not completely inhibited (${\mathrm{IC}}_{50}$ = 0.60 mM). The inhibition of fungal tyrosinase by anisic acid is a reversible reaction in which the tested acid is a non-competitive inhibitor ($K_{i}$ =0.603 mM). The modification of the alkyl chain in anisic acid influenced the activity and type of inhibition of the tested compounds. P-ethoxybenzoic acid showed a kind of incompetent inhibition, p-propoxybenzoic acid was of mixed, and p-butoxybenzoic acid was of competitive. With the increase in the alkyl chain’s length, the inhibitory activity of the tested compounds decreased^91^^,^^92^.

Chen et al. obtained similar results when testing p-alkoxybenzoic acid derivatives. The tested compounds behave as reversible tyrosinase inhibitors in the presence of L-DOPA substrate (λ  475 nm; spectrophotometric method, 6680 U/mg). Among them, *p*-hydroxybenzoic acid (${\mathrm{IC}}_{50}$= 1.3 mM; $K_{i}$ = 0.73 mM) is a competitive inhibitor, *p*-methoxybenzoic acid (${\mathrm{IC}}_{50}$=0.42 mM; $K_{i}$ = 0.43/0.43 mM) is non-competitive, *p*-ethoxybenzoic acid (${\mathrm{IC}}_{50}$=1.1 mM; $K_{i}$ = 1.46/0.84 mM) is of the mixed type, and the others show a type of non-competitive inhibition (*p*-propoxybenzoic acid, *p*-butoxybenzoic acid, *p*-pentoxybenzoic acid, and *p*-hexyloxybenzoic acid). Additionally, it was appeared that increasing the chain length above two carbon atoms changed the type of braking from competitive to non-competitive^93^. (22)

Another study^94^ determined the effect of methoxylation of hydroxyl groups on the tested acids’ activity and their esters. Protocatechuic acid methyl ester, protocatechuic acid, vanillic acid methyl ester, vanillic acid, isovanillic acid methyl ester, isovanillic acid, veratric acid methyl ester, and veratric acid were used in the study. Only protocatechuic acid and its methyl ester inhibited the enzyme (60.1 and 75.4% inhibition; ${\mathrm{ID}}_{50}\;$=0.42 µmol/mL and 0.28 µmol/mL). The hydroxyl group at the para and meta position is an important part of the structure of inhibitors. Protocatechuic acid methyl ester inhibited the enzyme activity more strongly than protocatechuic acid, which may result from the esterification of the carboxyl group.

Kubo et al. provided more information about the impact of an esterification on the activity of gallic acid. It was appeared that, apart from gallic acid (4.5 mM), the ${\mathrm{IC}}_{50}$ of all esters was almost comparable (<0.5 mM). Based on the above observation, it can be concluded that gallic acid esters with an increase in the number of carbon atoms of the alkyl chain (>C10) may be more challenging to incorporate into the protein pocket to reduce the rate of oxidation by the enzyme. Hence, gallates with a longer alkyl chain (>C-10) become inhibitors but not substrates, which indicates that gallates with a longer alkyl group (>C-10) can be expected as more suitable inhibitors. However, tetradecanoyl gallate (C-14) and hexadecanyl gallate (C-16) are sparingly soluble in water (Figure 12; Tables 1S and Table 13)^95^.

**Table 13.** An anti-tyrosinase activity of hydroxybenzoic and hydroxycinnaminic acids and their derivatives (NR-not reported; a-L-DOPA; b-L-tyrosin).

Substances	Substrate	Positive control (inhibitor)	Type of inhibition	Inhibition constant $K_{i}$ (mM)	Source of tyrosinase	The half-maximal inhibitory concentration ${\mathrm{IC}}_{50}$(mM)	References
**Hydroxybenzoic acids**							
Anisic acid (p-methoxybenzoic acid)	L- DOPA	NR	Non-competitive	0.603	Mushroom	0.6	Kubo et al.^90^
p-Hydroxybenzoic acid	L- DOPA	NR	Competitive	0.73	Mushroom	1.3	Kubo et al.^90^
p-Methoxybenzoic acid	L- DOPA	NR	Non-competitive	0.43/0.43	Mushroom	0.42	Chen et al.^92^
p-Ethoxybenzoic acid	L- DOPA	NR	Non-competitive	NR	Mushroom	NR	Kubo et al.^90^
	L- DOPA	NR	Mixed	1.46/0.84	Mushroom	1.1	Chen et al.^92^
p-Propoxybenzoic acid	L- DOPA	NR	Mixed	NR	Mushroom	NR	Kubo et al.^90^
	L- DOPA	NR	Non-competitive	0.84	Mushroom	1.85	Kubo et al.^90^
p-Butoxybenzoic acid	L- DOPA	NR	Competitive	NR	Mushroom	NR	Kubo et al.^90^
	L- DOPA	NR	Non-competitive	0.71	Mushroom	1.65	Chen et al.^92^
p-Pentyloxybenzoate acid	L- DOPA	NR	Non-competitive	0.6	Mushroom	1.4	Kubo et al.^90^
p-Hexyloxybenzoic acid	L- DOPA	NR	Non-competitive	0.49	Mushroom	1.15	Chen et al.^92^
Gallic acid	L- DOPA	NR	NR	NR	Mushroom	4.5	Kubo et al. ^94^
Methyl gallate	L- DOPA	NR	NR	NR	Mushroom	0.35	Kubo et al. ^94^
Propyl gallate	L- DOPA	NR	NR	NR	Mushroom	0.31	Kubo et al. ^94^
Hexyl gallate	L- DOPA	NR	NR	NR	Mushroom	0.21	Kubo et al. ^94^
Octyl gallate	L- DOPA	NR	NR	NR	Mushroom	0.33	Kubo et al. ^94^
Decyl gallate	L- DOPA	NR	NR	NR	Mushroom	0.28	Kubo et al. ^94^
Dodecyl gallate	L- DOPA	NR	Mixed	NR	Mushroom	0.49	Kubo et al. ^94^
**Hydroxycinnaminic acids**							
Cinnamic acid	L- DOPA	NR	Non-competitive	1.99	Mushroom	2.1	Shi et al.^95^
2-Hydroxycinnamic acid	L- DOPA	NR	Not effective	Not effective	Mushroom	Not effective	Shi et al.^95^
4-Hydroxycinnamic acid	L- DOPA	NR	Competitive	0.244	Mushroom	0.5	Shi et al.^95^
4-Methoxycinnamic acid	L- DOPA	NR	Non-competitive	0.458	Mushroom	0.42	Shi et al.^95^
3,4-Dihydroxycinnamic acid	L- DOPA	NR	Non-competitive	NR	Mushroom	0.33	Garcia-Jimenez et al.^96^
4-Hydroxy-3-methoxycinnamic acid	L- DOPA	NR	Non-competitive	NR	Mushroom	0.33	Garcia-Jimenez et al.^96^
2-Methoxycinnamic acid	L- DOPA L-Tyrosine	NR	Competitive	$0.51^{a}$/$0.5^{b}$	Mushroom	NR	Garcia-Jimenez et al.^96^
3-Methoxycinnamic acid	L- DOPA L-Tyrosine	NR	Competitive	$0.68^{a}$/$0.69^{b}$	Mushroom	NR	Garcia-Jimenez et al.^96^
4-Methoxycinnamic acid	L- DOPA L-Tyrosine	NR	Competitive	$1.47^{a}$/$1.54^{b}$	Mushroom	NR	Garcia-Jimenez et al.^96^
Protocatechuic acid methyl ester	L-Tyrosine	NR	NR	NR	Mushroom	NR	Miyazawa et al.^93^
Protocatechuic acid	L-Tyrosine	NR	NR	NR	Mushroom	NR	Miyazawa et al.^93^
Vanillic acid	L-Tyrosine	NR	NR	NR	Mushroom	NR	Miyazawa et al.^93^
Vanillic acid methyl ester	L-Tyrosine	NR	NR	NR	Mushroom	NR	Miyazawa et al.^93^
Isovanillic acid	L-Tyrosine	NR	NR	NR	Mushroom	NR	Miyazawa et al.^93^
Isovanillic acid methyl ester	L-Tyrosine	NR	NR	NR	Mushroom	NR	Miyazawa et al.^93^
Veratric acid	L-Tyrosine	NR	NR	NR	Mushroom	NR	Miyazawa et al.^93^
Veratric acid methyl ester	L-Tyrosine	NR	NR	NR	Mushroom	NR	Miyazawa et al.^93^
Ferulic acid	L-Tyrosine	Hydroquinone	NR	NR	Mushroom	NR	Maruyama et al.^97^
Caffeic acid	L-Tyrosine	Hydroquinone	NR	NR	Mushroom	NR	Maruyama et al.^97^
p-Coumaric acid	L-Tyrosine	Kojic acid Arbutin	NR	NR	Mushroom	0.115	Maruyama et al.^97^

##### 5.2.1.2. Hydroxycinnaminic acids

Shi et al. investigated the effect of cinnamic acid and its derivatives on the activity of fungal tyrosinase. Cinnamic acid (${\mathrm{IC}}_{50}$=2.10 mM), 4-methoxycinnamic acid (${\mathrm{IC}}_{50}$=0.42 mM), and 4-hydroxycinnamic acid (${\mathrm{IC}}_{50}$ =0.52 mM) strongly inhibited the conversion of diphenol to dichinone. Cinnamic acid and 4-methoxycinnamic acid showed a non-competitive type of inhibition (*Ki* = 1.994 and 0.458 mM), interacting with a different site of the enzyme than the active site. In contrast, 4-hydroxycinnamic acid competitively inhibited the enzyme (*Ki* = 0.244), which may result from the similar structure of the tested compound to the tyrosinase substrate. One of the tested compounds, i.e. 2-hydroxycinnamic acid did not inhibit the enzyme activity, probably because of the presence of the 2-OH group causing spherical hindrances^96^.

Another study^97^ assessed the influence of methoxylation of cinnamic acid and its derivatives (cinnamic acid, 2-hydroxycinnamic acid, 3-hydroxycinnamic acid, 4-hydroxycinnamic acid, 3,4-dihydroxycinnamic acid, 2-methoxycinnamic acid, 3-methoxycinnamic acid, and 4-methoxycinnamic acid; concentrations from 0.1 to 3 mmol/L) on the action of tyrosinase (3130 U/mg) in the presence of L-tyrosine or L-DOPA as a substrate. Cinnamic acid (${\mathrm{IC}}_{50}$=2.1 mM), 2-hydroxycinnamic acid (${\mathrm{IC}}_{50}$=0.5 mM), and the O-methyl (${\mathrm{IC}}_{50}$=0.42 mM) forms exhibited inhibitory properties compared to 3-hydroxycinnamic acid, 4-hydroxycinnamic acid, and 3,4-dihydroxycinnamic acid, which turned out to be substrates of tyrosinase. All inhibitors showed competitive inhibition. The inhibition constants are about the same for the oxidation of L-tyrosine and L-DOPA, indicating that the inhibitors bind to the same form of the enzyme. In the next study^98^, the inhibitory effect of caffeic acid and ferulic acid on tyrosinase activity isolated from murine B16 melanoma cells (90 U) was analysed. Both ferulic acid (27.4% non-toxic conc.) and caffeic acid (24.4% non-toxic conc.) effectively inhibited melanin production in B16 melanoma cells. Ferulic acid was reducing tyrosinase activity by binding directly to the enzyme, whereas no binding was observed between caffeic acid and tyrosinase.

One of the important structural elements regulating the enzyme’s activity is a type of bonds present in the inhibitor’s structure. Some compounds, such as p-coumaric acid (${\mathrm{IC}}_{50}$=115.6 µM; % inhibition 74.4) and isoferulic acid (${\mathrm{IC}}_{50}$=114.9 µM; % inhibition 77.8) lose their tyrosinase inhibitory properties after saturation of the double bond (dihydro-p-coumaric acid ${\mathrm{IC}}_{50}$=1000 µM; 74.4% inhibition and dihydroisoferulic acid ${\mathrm{IC}}_{50}$=195.7 µM; 60.6% inhibition). None of the compounds tested were more effective than kojic acid (${\mathrm{IC}}_{50}$=51.6 µM) or arbutin (${\mathrm{IC}}_{50}$=210.5 µM). All compounds tested show a non-competitive type of inhibition. The results indicate that the reduction of double bonds weakens the inhibitory activity of the test compounds. The C = C binding is necessary for the proper interaction of the inhibitor with the active site^99^.

**Figure 15.**
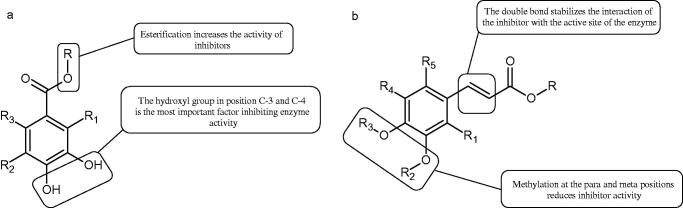
a) Potential groups engaged in an interaction hydroxybenzoic acid-tyrosinase and b) Potential groups engaged in an interaction hydroxycinnamic acid-tyrosinase.

In another study, the type of inhibition and the effect of caffeic acid, *p*-coumaric acid, and rosmarinic acid on monophenolase and diphenalase activities were determined. The most active compound was *p*-coumaric acid (L-tyr, IC_50 _=0.3 µM; L-DOPA, IC_50 _=0.62 µM), which inhibited the enzyme noncompetitively (L-tyr, *K_i_*=0.033 µM; L-DOPA, *K_i_*=2.2 µM) followed by caffeic acid (L-tyr, IC_50 _=1.50 µM; L-DOPA, IC_50 _=2.30 µM) and rosmarinic acid (L-tyr, IC_50 _=4.14 µM; L-DOPA, IC_50 _=8.59 µM). These compounds blocked the enzyme more potently than kojic acid (L-tyr, IC_50 _=33.45 µM; L-DOPA, IC_50 _=38.98 µM) (Figure 15; Tables 2S and Table 13)^100^.

#### 5.2.2. Flavonoids

Flavonoids belong to a group of compounds commonly found in the plant world. These compounds differ from each other by the presence of a double bond between the second and third carbon atom, a ketone group at position 4, and the position of the B ring. Additionally, individual flavonoids differ from hydroxyl, methyl, isoprenoid, and methoxy groups arranged in different rings. Many flavonoids have tyrosinase inhibitory activity^101^.

##### 5.2.2.1. Hydroxyl groups

The distribution and number of hydroxyl groups in flavonoid molecules significantly affect their activity. Chrysin (flavone), having no hydroxyl groups in the B-ring, does not block the activity of tyrosinase. The presence of the hydroxyl group in the C-4′ position of apigenin (${\mathrm{IC}}_{50}$=c. 40 µM) significantly increased its activity compared to chrysin. The additional hydroxyl group in the C-3′ position of luteolin (${\mathrm{IC}}_{50}$=c. 186 µM) decreased its activity compared to apigenin. Galagin (${\mathrm{IC}}_{50}$=c. 10 µM) without hydroxyl groups on the B-ring blocks tyrosinase activity more strongly than chrysin. Addition of the hydroxyl group at the C-4′ or C-3′, C-4′ position reduces the activity of kaempferol (${\mathrm{IC}}_{50}$=c. 73 µM) and quercetin (${\mathrm{IC}}_{50}$=c. 30 µM) compared to galagin. Flavonols, due to the additional hydroxyl group at the C-3 position, block tyrosinase much more strongly than flavones^102^.

Kim et al. provided more information regarding the influence of the position and number of hydroxyl groups on their inhibitory activity. Natural and synthetic flavones and flavonols were used in the study. L-tyrosine was used as a substrate for fungal tyrosinase. It appears that the hydroxyl groups at the C-7 (ring A) and C-4′ (ring B) positions may increase the inhibitory activity of flavonoids. In this study, 3′,4′,7,8-tetrahydroxy flavone showed the strongest inhibitory properties (${\mathrm{IC}}_{50}$=0.07 µM). Two catechol groups in ring A and B are probably responsible for the inhibition. Lack of the fourth hydroxyl group in 4′′,7,8-trihydroxy flavone (${\mathrm{IC}}_{50}$=12.95 µM) and 3′′,4′′,7-trihydroxy flavone (${\mathrm{IC}}_{50}$=24.1 µM) weakens their activity. The 5-OH group also has a significant influence on the activity of the inhibitors, e.g. 2′,5,7-trihydroxyflavone (${\mathrm{IC}}_{50}$=12.95 µM) and 3′,4′,5,7-tetrahydroxy flavone (${\mathrm{IC}}_{50}$=12.95 µM) block tyrosinase more strongly than 2′,7-dihydroxy flavone, and 3′,4′,7-trihydroxy flavone. Also, the hydroxyl group in the C-3 position, which divides flavonoids into flavones and flavonols, influences on the activity with a weaker inhibition for flavones. Additionally, an increase in the number of hydroxyl groups in flavonoids reduces their inhibitory activity against tyrosinase (3′,4′,5,6,7-pentahydroxy flavonol ${\mathrm{IC}}_{50}$=314.23 µM). The location of the hydroxyl groups plays a more important role than their number. Summarising the increase in the number of hydroxyl groups on the other side of the flavonoids (C-7, C-8, C-2′, C-3′, and C-4′) increases compounds’ activity^103^. In contrast, an increase in the number of hydroxyl groups on the ketone side (C-5, C-6, C-5′, C-6′) reduces the activity of inhibitors, which is related to the disturbance of the interaction with tyrosinase. Isoflavonoids are characterised by a linked ring B at the third carbon atom. The location and number of hydroxyl groups in the A-ring of isoflavonoids can strongly affect both the inhibitory power and inhibition type. 6,7,4′-trihydroxyisoflavone, daidzein, glycitin, daidzin and genistin showed strong monophenolase inhibitory activity but weak diphenolase inhibitory activity. 4′,6,7-trihydroxyisoflavone (${\mathrm{IC}}_{50}$=9 µM) shows the strongest properties. Presence of a single hydroxyl group in the C-7 position (4′,7-trihydroxyisoflavone ${\mathrm{IC}}_{50}$=203 µM; 6-methoxy-7,4′-dihydroxyisoflavone ${\mathrm{IC}}_{50}$=218 µM) or no hydroxyl groups (4′-hydroxyisoflavone-7-O-glucoside ${\mathrm{IC}}_{50}$=267 µM) in ring A significantly reduces the activity of the compounds. The presence of the hydroxyl groups in the C-7 and C-8 position can influence the type of inhibition, shifting from reversible to irreversible type^104^. Similar results were obtained in case of anthocyanins and an assessment of the impact of the number and distribution of hydroxyl groups in the B ring on tyrosinase activity. The following compounds were investigated: pelargonidin (${\mathrm{IC}}_{50}$=66 µM), cyanidin (${\mathrm{IC}}_{50}$=27.1 µM), and delphinidin (${\mathrm{IC}}_{50}$=57.4 µM) in the presence of kojic acid (${\mathrm{IC}}_{50}$=34.8 µM) as a positive control. The substrate for the reaction was L-DOPA. These results indicate that the structure with two hydroxyl groups in ring B has the greatest inhibitory effect^105^.

Another study examined the effect of isoflavonoids isolated from the roots of *Pueraria lobata*, such as daidzein and formononetin. These compounds have appeared to be of weak inhibitors with the IC_50_ values for daidzein L-tyr. 350 µM; L-DOPA 350 µM and for formononetin L-tyr. IC_50 _>350 µM; L-DOPA IC_50 _>350 µM. A significant increase in an activity was obtained by introducing OH groups into the B ring at the C-3′ position and methylation of the 4′-OH group - calycosin (L-tyr. IC_50 _=7.02 µM; L-DOPA IC_50 _=1.45 µM). This compound is more active than kojic acid (L-tyr. IC_50 _=12.10 µM; L-DOPA IC_50 _=9.14 µM). It is seen that the presence of a hydroxyl group at the C-3′ position and a methoxyl group at the C-4′ position of the isoflavone backbone plays a major role in the anti-tyrosine activity^106^. The inhibitory activity of calycosin against tyrosinase (monophenolase) was also confirmed by Kim et al. with the IC_50_ equal of 38.4 µM. In turn, the IC_50_ value for kojic acid and arbutin was 51.5 and 120.9 µM, respectively^107^. However, calycosin was more toxic than standards (LD_50 _=120 µM *vs.* LD_50 _>200 µM for kojic acid and arbutin). The results of another study conducted by Kim et al. have also shown an inhibitory activity of calycosin with the IC_50 _=30.8 µM and for kojic acid IC_50 _=50_._1 µM^108^.

The isoflavonoids isolated from the stem of *Maackia fouriei* have also exhibited their anti-tyrosinase activity. Methylation of the 4′-OH group in formononetin, texasin, and odoratin, resulted in impaired anti-tyrosinase properties. The presence of 5-OH group in genistein (IC_50_=33 µM) and tectorigenin (IC_50_=20 µM) favourably affects the activity of isoflavonoids in comparison to daidzein (IC_50_=41 µM) without 5-OH group. An interesting compound isolated from *M. fouriei* is the bishomoflavonoid derivative, mircoin (IC_50_=5 µM). This compound inhibited the enzyme competently. Due to its strong inhibition, further structure-activity studies are needed for this compound. In this study, kojic acid (IC_50_=45 µM) was used as a positive control^109^.

In another study, the effect of OH groups at the C-6, C-7 and C-4′ positions on isoflavonoid activity was investigated. 6,7,4′ -Trihydroxyisoflavone inhibited tyrosinase competently (IC_50_=9 µM; *K_i_* value of 5.72–6.24 µM). Methylation of the 6-OH group in glycitein (IC_50_=264 µM), the absence of the 6-OH group in daidzein (IC_50_=237 µM), or the presence of an OH group at the 5-OH position in genistein (IC_50_=822 µM), decreased the anti-tyrosinase activity (Table 14)^110^.

**Table 14.** Structure and activity of flavonoids with an anti-tyrosinase activity.

Structure	Name	IC_50_ (μΜ)	References
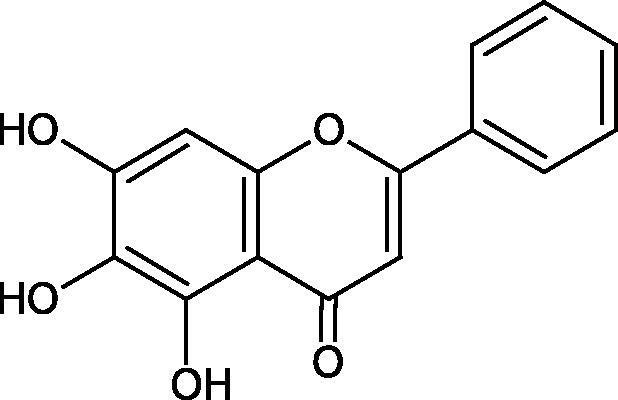	Baicalein	290	Yin et al.^113^
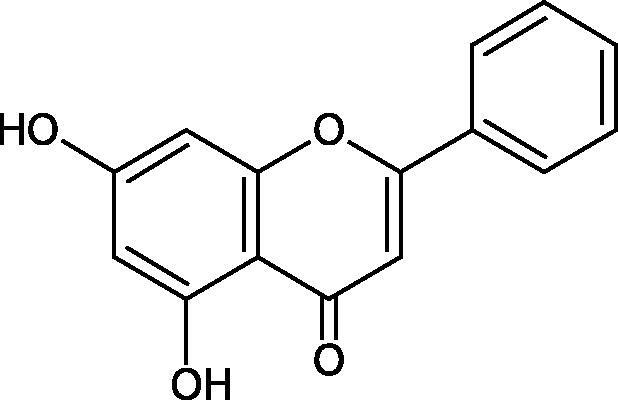	Chrysin	–	Yin et al.^113^
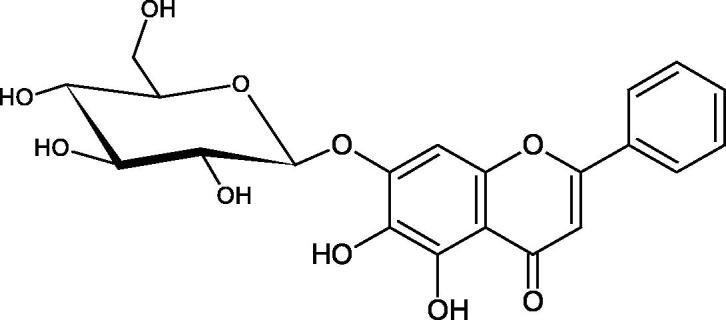	Oroxin A	500	Yin et al.^113^
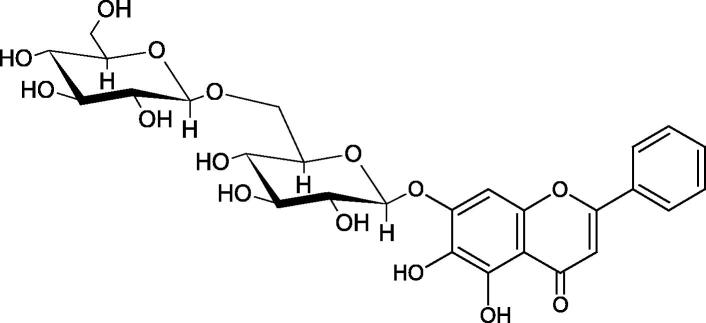	Oroxin B	–	Yin et al.^113^
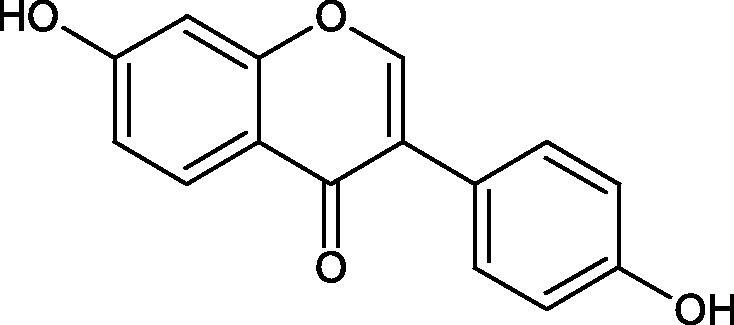	Daidzein	>350^a,b^; 41^c^	Wagle et al.^105^; Kim et al.^107^
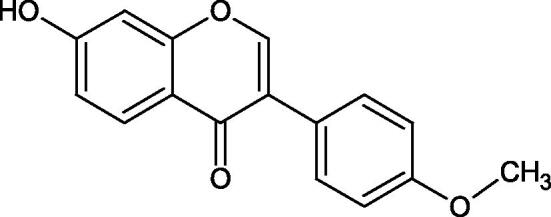	Formononetin	>350^a,b^; –^c^	Wagle et al.^105^; Kim et al.^107^
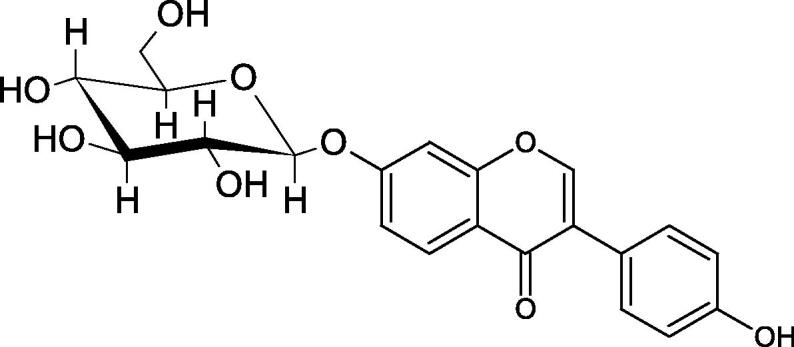	Daidzin	310.67^a^, >350^b^	Wagle et al.^105^
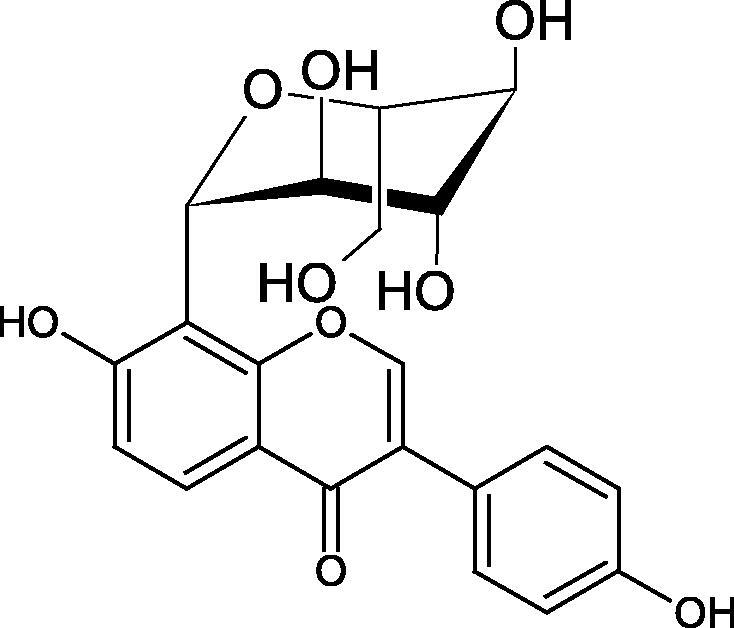	Puerarin	438.13^a^, >350^b^	Wagle et al.^105^
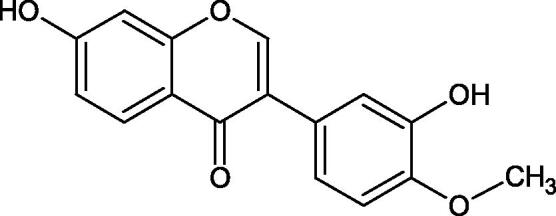	Calycosin	7.02^a^, 1.45^b^/38.4^d^	Wagle et al.^105^; Kim et al.^106^
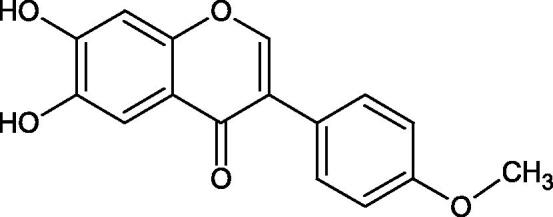	Texasin	–^c^	Kim et al.^107^
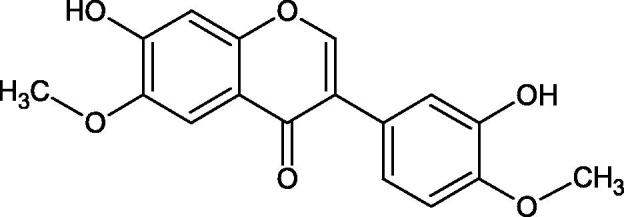	Odoratin	–^c^	Kim et al.^107^
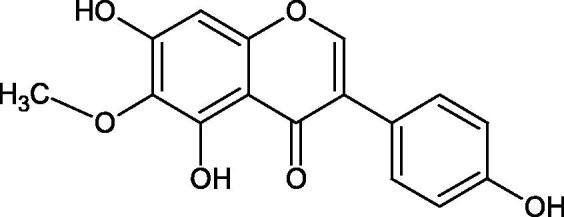	Tectorigenin	20^c^	Kim et al.^107^
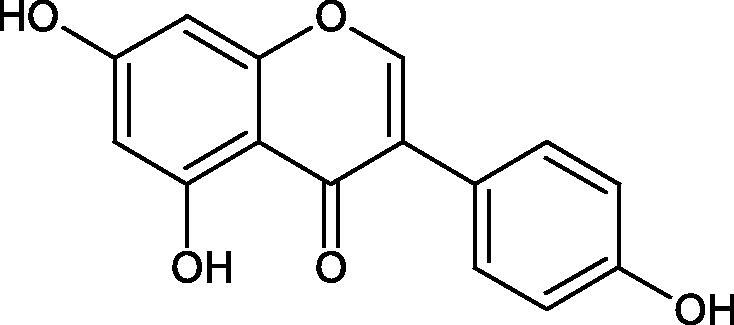	Genistein	362.54^a^, >350^b^; 33^c^	Wagle et al.^105^; Kim et al.^107^
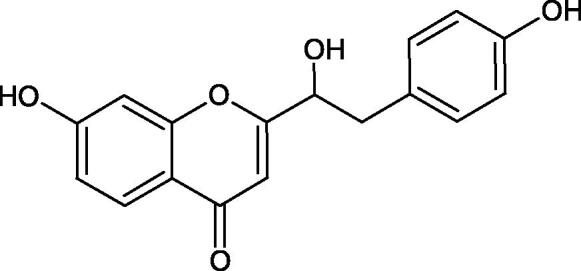	Mirkoin	5^c^	Wagle et al.^105^; Kim et al.^107^
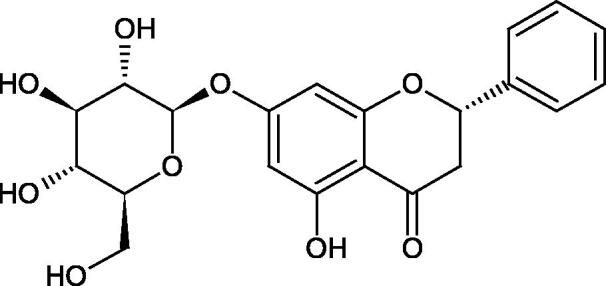	(2*S*)-pinocembrin-7-*O*-*β*-d-glycoside	115.35^a^, 122.34^b^	Yang et al.^152^
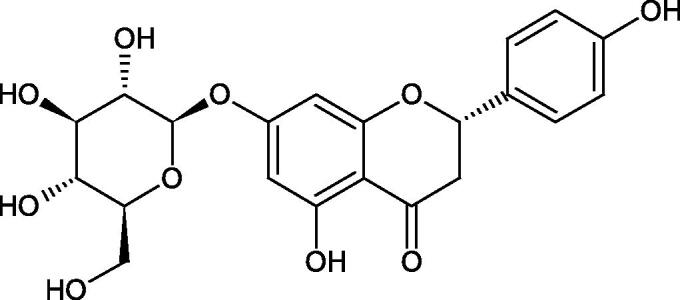	(2*S*)-naringenin-7-*O*-*β*-D-glycoside	27.49^a^, 39.26^b^	Yang et al.^152^
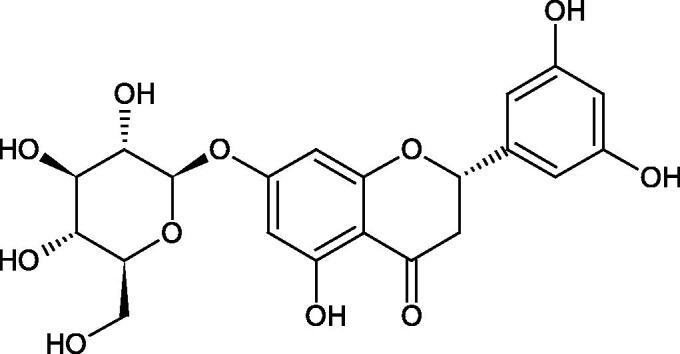	(2*S*)-5,7,*3′*,*5′*-tetrahydroxy-flavanone 7-*O*-*β*-D-glucopyranoside	16.49^a^, 20.38^b^	Yang et al.^152^

^a^L-tyrosine.^b^L-DOPA.^c^L-tyrosine for Kim et al.^108^.^d^L-tyrosine for Kim et al.^107^.

Similar effects of isoflavonoids on tyrosinase were noted by studying extracts of *Otholobium pubescens* (Pior.) J.W. Grimes. In this study, L-tyrosine as substrate and β-arbutin (IC50 = 1830 µM) as a control were used. Daidzein and its aglycone, genistein showed no effect (daidzein, IC_50 _=1580 µM; genistein, IC_50 _=7660 µM)^111^.

**Figure 16.**
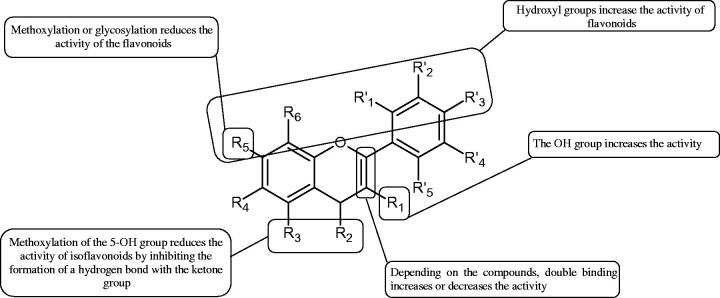
Potential groups engaged in an interaction flavonoid-tyrosinase.

**Figure 17.**
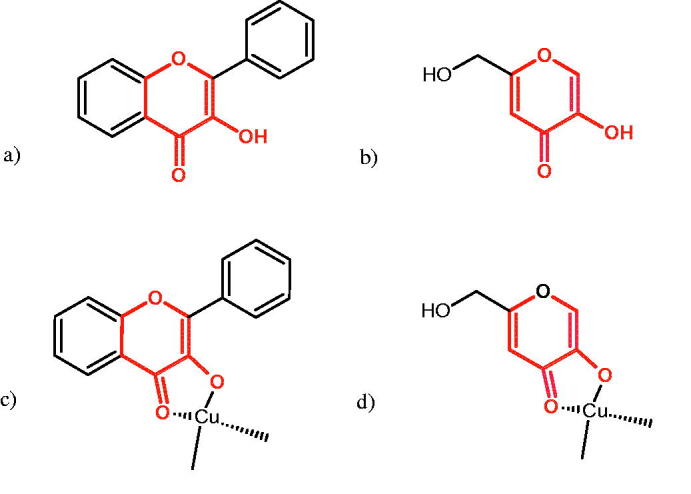
Structure relationship between flavanol (a) and kojic acid (b) and mode of copper chelation by 3-hydroxy-4-keto group in flavanol (c), and kojic acid (d).

The discrepancies in the study are due to the variation in experimental conditions such as temperature, pH, and concentrations of enzyme and substrates used. Flavonols are the most potent tyrosinase inhibitors among flavonoids. This is due to the similarity of flavonols to the structure of kojic acid − 3-hydroxy-4-keto moiety (Figures 16 and 17).

##### 5.2.2.2. Methoxylation

Methoxylation of the hydroxyl groups in the flavonoid molecules reduces their activity. Methoxylated flavones such as 5,6,7,4′-tetramethylscutellarein (5.21% inhibition), 5,7,4′-trimethylscutellarein (3.23% inhibition), and ladanein (4.24% inhibition) showed a ten-fold decrease in the inhibitory activity than kojic acid (% inhibition 80.02). It was proved that methoxylation of isoflavonoid also decreases the inhibition e.g. 2′-hydroxygenistein (37.3% inhibition) inhibited tyrosinase stronger than its methylated forms, such as 5-O-methyl-2′-hydroxygenistein (25.8% inhibition) and 7-O-methyl-2′-hydroxygenistein (31.2% inhibition). The weaker activity of 5-O-methyl-2′-hydroxygenistein may be related to the disturbance of a hydrogen bond formation between 5-OH and carbonyl oxygen (C-4). Kojic acid (${\mathrm{IC}}_{50}$=11.3 µM) was used as a control and L-DOPA as a substrate^112^.

More information about the effect of hydroxyl groups and methylation on flavonoid activity was provided by studying derivatives having a methoxy group at position 3, a hydroxy group at position 5, and oxidised aromatic carbons at C4′ and C7 (Table 15). The most potent inhibitors 1 (IC_50 _=6.71 µM), 2 (IC_50 _=13.20 µM), and 3 (IC_50 _=17.66 µM) have three hydroxy groups at the C-3′, C-4′ and C-5′ positions in the B ring. Compound 1, which contains an additional methoxy group at C6 and a hydroxy group at C7, was the most active. Comparing the IC_50_ of 1 *vs.* 2 and 5 (IC_50 _=73.03 µM) *vs.* 6 (IC_50 _=103.56 µM), it seems that compounds containing a methoxy group at position C6 are more active than those that are unsubstituted at this position (Table 15)^113^.

**Table 15.** Effect of the position and number of hydroxyl groups on the activity of flavonoids towards tyrosinase.

	Name	R_6_	R_7_	R'_3_	R'_4_	R'_5_	IC_50_ (μM)
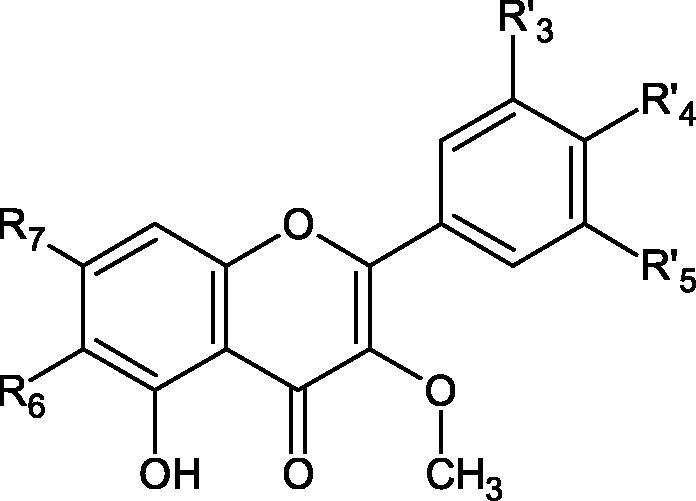	1	OCH_3_	OH	OCH_3_	OCH_3_	OH	6.71
	2	H	OH	OCH_3_	OCH_3_	OH	13.20
	3	H	OCH_3_	OCH_3_	OCH_3_	OH	17.66
	4	OCH_3_	OH	OCH_3_	OCH_3_	OCH_3_	62.68
	5	OCH_3_	OH	H	OH	H	73.03
	6	H	OH	H	OH	H	103.56

##### 5.2.2.3. Double bond

The double bond between the second and third carbon atoms is preferred for flavonoids to maintain a flat molecular structure. Naringenin (${\mathrm{IC}}_{50}$=c. 555 µM) showed less inhibitory activity than apigenin (${\mathrm{IC}}_{50}$=c. 40 µM). Dihydromyricetin (${\mathrm{IC}}_{50}$=c. 37 µM) showed greater inhibitory activity than myricitin (${\mathrm{IC}}_{50}\;$=c. 85 µM), while taxifolin (${\mathrm{IC}}_{50}$=c. 800 µM) showed less inhibitory activity than quercetin (${\mathrm{IC}}_{50}$=c. 30 µM). These results suggested that C2 = C3 binding affects the inhibitory properties of the flavonoids^102^.

##### 5.2.2.4. Glycosides

Flavonoids occur mainly in the form of 3- and 7-glycosides. Some studies have revealed that a sugar moiety can modify flavonoids’ activity, e.g. 3-O-glycosides, hyperin (${IC}_{50}$ not detected) and rutin (${\mathrm{IC}}_{50}$=c. 4571 µM) show weaker tyrosinase inhibition than aglycone – quercetin (${\mathrm{IC}}_{50}$=c. 30 µM). Similarly, 7-O-glycosides, baicalin (${\mathrm{IC}}_{50}$=c. 215 µM) and naringin (${\mathrm{IC}}_{50}$=c. 1900 µM) also inhibited tyrosinase weaker. A clear evidence was provided when monoglycosides, diglycosides and acylated monoglycosides towards tyrosinase inhibition were tested. Monoglycosides such as luteolin-7-O-glucoside (27.35% inhibition, ${\mathrm{IC}}_{50}$=74 µM), kaempferol-3-O-glucoside (24.2% inhibition, ${\mathrm{IC}}_{50}$=74 µM), and isorhamnetin-3-O-glucoside (24.22% inhibition, ${\mathrm{IC}}_{50}$=70 µM) showed stronger inhibition tyrosinases in a comparison to diglycosides such as kaempferol-3-O-rutinoside (16.05% inhibition, ${\mathrm{IC}}_{50}$=56 µM), isorhamnetin-3-O-rutinoside (13.13% inhibition, ${\mathrm{IC}}_{50}$=53 µM), and rutin (12.65% inhibition, ${\mathrm{IC}}_{50}$=55 µM). It is suggested that a presence of acyl groups on sugar residues of monoglycosides kaempferol-3-O-(6′′-pCm)-glucoside (14.69% inhibition, ${\mathrm{IC}}_{50}$=11 µM), quercetin-3-O-(6′′-pCm) -glucoside (21.86% inhibition, ${\mathrm{IC}}_{50}$=55 µM), isorhamnetin-3-O-(6′′-OAc)-glucoside (23.31% inhibition, ${\mathrm{IC}}_{50}$=64 µM), isorhamnetin-7-O-(6′′-pCm)-glucoside (21.10% inhibition, ${\mathrm{IC}}_{50}$=53 µM), apigenin-7-O-(6′′-pCm)-glucoside (17.66% inhibition, ${\mathrm{IC}}_{50}$=58 µM), apigenin-7-O-(3′′,6′′-di-pCm)-glucoside (20.69% inhibition, ${\mathrm{IC}}_{50}$=46 µM), chrysoeriol-7-O-(3′′,6′′-di-pCm)-glucoside (15.59% inhibition, ${\mathrm{IC}}_{50}$=44 µM) promotes an inhibitory effect compared to mono and diglycosides. The increase in the size of the flavonoids may prevent the flavonoids from entering the active site of tyrosinase (Tables 3S and Table 16)^102^.

**Table 16.** An anti-tyrosinase activity of flavonoids (UE-unable to establish; c-with respect to L-tyrosine; d-with respect to both L-tyrosine and L-DOPA; NR-not reported; NT-not tested).

Substances	Substrate	Positive control (inhibitor)	Type of inhibition	Inhibition constant $K_{i}$	Source of tyrosinase	The half-maximal inhibitory concentration ${\mathrm{IC}}_{50}$ (µM)	References
FLavonols							
Galangin,	L-DOPA	Koijc acid	Competitive	NR	Mushroom	10	Fan et al.^101^
Kaempferol	L-DOPA	Koijc acid	Competitive	NR	Mushroom	73	Fan et al.^101^
Isorhamnetin	L-DOPA	Koijc acid	Mixed	NR	Mushroom	303	Fan et al.^101^
Rutin	L-DOPA	Koijc acid	Competitive	NR	Mushroom	4571	Fan et al.^101^
Myricetin	L-DOPA	Koijc acid	Mixed	NR	Mushroom	85	Fan et al.^101^
Quercetin	L-DOPA	Koijc acid	Competitive	NR	Mushroom	30	Fan et al.^101^
Morin	L-DOPA	Koijc acid	Competitive	NR	Mushroom	85	Fan et al.^101^
Diosmetin	L-DOPA	Koijc acid	Competitive	NR	Mushroom	417	Fan et al.^101^
Hyperin	L-DOPA	Koijc acid	NT	NR	Mushroom	NT	Fan et al.^101^
DIHYDROFLAVONES							
Naringenin	L-DOPA	Koijc acid	Non-competitive	NR	Mushroom	555	Fan et al.^101^
Naringin	L-DOPA	Koijc acid	Competitive	NR	Mushroom	1900	Fan et al.^101^
FLAVONES							
Chrysin	L-DOPA	Koijc acid	NR	NR	Mushroom	Non effect	Fan et al.^101^
Chrysoeriol	L-DOPA	Koijc acid	NR	NR	Mushroom	83	Fan et al.^101^
Ladanein	L-DOPA	Koijc acid	NR	NR	Mushroom	53	Fan et al.^101^
5,6,7,8,4′-Pentahydroxyflavone	L-DOPA	Koijc acid	NR	NR	Mushroom	55	Fan et al.^101^
5,6,7,4′-Tetramethylscutellarein	L-DOPA	Koijc acid	NR	NR	Mushroom	49	Fan et al.^101^
5,7,4′-Trimethylscutellarein	L-DOPA	Koijc acid	NR	NR	Mushroom	50	Fan et al.^101^
Luteolin	L-DOPA	Koijc acid	Non-competitive		Mushroom	186	Fan et al.^101^
Luteolin-7-O-glukozyd	L-DOPA	Koijc acid	NR	NR	Mushroom	74	Karioti et al.^111^
Kaempferol-3-O-glucoside	L-DOPA	Koijc acid	NR	NR	Mushroom	74	Karioti et al.^111^
Isorhamnetin-3-O-glucoside	L-DOPA	Koijc acid	NR	NR	Mushroom	70	Karioti et al.^111^
Kaempferol-3-O-rutinoside	L-DOPA	Koijc acid	NR	NR	Mushroom	56	Karioti et al.^111^
Isorhamnetin-3-O-rutinoside	L-DOPA	Koijc acid	NR	NR	Mushroom	53	Karioti et al.^111^
6-OH-kaempferol-3-O-rutinoside	L-DOPA	Koijc acid	NR	NR	Mushroom	55	Karioti et al.^111^
Kaempferol-3-O-(6′’-pCm)-glucoside	L-DOPA	Koijc acid	NR	NR	Mushroom	11	Karioti et al.^111^
Quercetin-3-O-(6′’-pCm)-glucoside	L-DOPA	Koijc acid	NR	NR	Mushroom	55	Karioti et al.^111^
Isorhamnetin-3-O-(6′’-OAc)-glucoside	L-DOPA	Koijc acid	NR	NR	Mushroom	64	Karioti et al.^111^
Isorhamnetin-7-O-(6′’-pCm)-glucoside	L-DOPA	Koijc acid	NR	NR	Mushroom	53	Karioti et al.^111^
Apigenin-7-O-(6′’-pCm)-glucoside	L-DOPA	Koijc acid	NR	NR	Mushroom	58	Karioti et al.^111^
Apigenin-7-O-(3′’,6′’-di-pCm)-glucoside	L-DOPA	Koijc acid	NR	NR	Mushroom	46	Karioti et al.^111^
Chrysoeriol-7-O-(3′’,6′’-di-pCm)-glucoside	L-DOPA	Koijc acid	NR	NR	Mushroom	44	Karioti et al.^111^
Apigenin	L-DOPA	Koijc acid	Mixed	NR	Mushroom	40	Fan et al.^101^
	L-DOPA	Koijc acid	NR	NR	Mushroom	93	Karioti et al.^111^
Baicalein	L-DOPA	Koijc acid	Non-competitive	NR	Mushroom	138	Fan et al.^101^
Baicalin	L-DOPA	Koijc acid	Mixed	NR	Mushroom	215	Fan et al.^101^
DIHYDROFLAVOLS							
Dihydromyricetin	L-DOPA	Koijc acid	Mixed	NR	Mushroom	37	Fan et al.^101^
Taxifolin	L-DOPA	Koijc acid	Competitive	NR	Mushroom	800	Fan et al.^101^
ISOFLAVONOIDS							
Daidzein	L-tyrosin L-DOPA	Koijc acid	${\text{Reversibly competitive}}^{c}$	19.4	Mushroom	203/UE	Chang et al.^109^
Genistein	L-DOPA	Koijc acid	Competitive	NR	Mushroom	25	Chang et al.^109^
6,7,4′-Trihydroxyisoflavone	L-tyrosin L-DOPA	Koijc acid	${\text{Reversibly competitive}}^{c}$	1.93	Mushroom	9/UE	Chang et al.^109^
7,8,4′-Trihydroxyisoflavone	L-tyrosin L-DOPA	Koijc acid	${\mathrm{Irreversible}}^{d}$	UE	Mushroom	191/184	Chang et al.^109^
5,7,8,4′-Tetrahydroxyisoflavone	L-tyrosin L-DOPA	Koijc acid	${\mathrm{Irreversible}}^{d}$	UE	Mushroom	181/212	Chang et al.^109^
6-Methoxy-7,4′-dihydroxyisoflavone (glycitein)	L-tyrosin L-DOPA	Koijc acid	${\text{Reversibly competitive}}^{c}$	50.6	Mushroom	218/UE	Chang et al.^109^
4′-Hydroxyisoflavone-7-O-glucoside (daidzin)	L-tyrosin L-DOPA	Koijc acid	${\text{Reversibly competitive}}^{c}$	15.1	Mushroom	267/UE	Chang et al.^109^
5,4′-Dihydroxyisoflavone-7-O-glucoside (genistin)	L-tyrosin L-DOPA	Koijc acid	${\text{Reversibly competitive}}^{c}$	17.6	Mushroom	343/UE	Chang et al.^109^
ANTHOCYANIDINS							
Pelargonidin	L-DOPA	Koijc acid	NR	NR	Mushroom	66	Tsuda and Osawa^104^
Pelargonidln 3-O-β-D-glucoside	L-DOPA	Koijc acid	NR	NR	Mushroom	61.2	Tsuda and Osawa^104^
Cyanidin	L-DOPA	Koijc acid	NR	NR	Mushroom	27.1	Tsuda and Osawa^104^
Cyanldin 3-O-β-D-glucoside	L-DOPA	Koijc acid	NR	NR	Mushroom	40.3	Tsuda and Osawa^104^
Delphinldin	L-DOPA	Koijc acid	NR	NR	Mushroom	57.4	Tsuda and Osawa^104^
Delphinldin 3-O-β-D-glucoside	L-DOPA	Koijc acid	NR	NR	Mushroom	46.2	Tsuda and Osawa^104^

Comparing baicalein (IC_50 _=290 µM) with chrysin (no activity), the other hydroxyl group at the C-6 position of baicalein results in more potent tyrosinase inhibition. Comparing the inhibitory potency of baicalein (aglycone) with its glycosides, oroxin B (no activity) and oroxin A (IC_50 _=500 µM), a decrease in an inhibitory activity was noted. Glycosylation of the hydroxyl group at C7 was negatively correlated with the inhibitory activity of flavonoids. In addition, the activity of glycosides was influenced by the type of sugar moiety. The presence of the *β*-D-gentiobiosyl group reduced the inhibitory activity stronger than *β*-D-glucopyranosyl. This effect is due to spherical collapses^114^.

#### 5.2.3. Lignans

**Figure 18.**
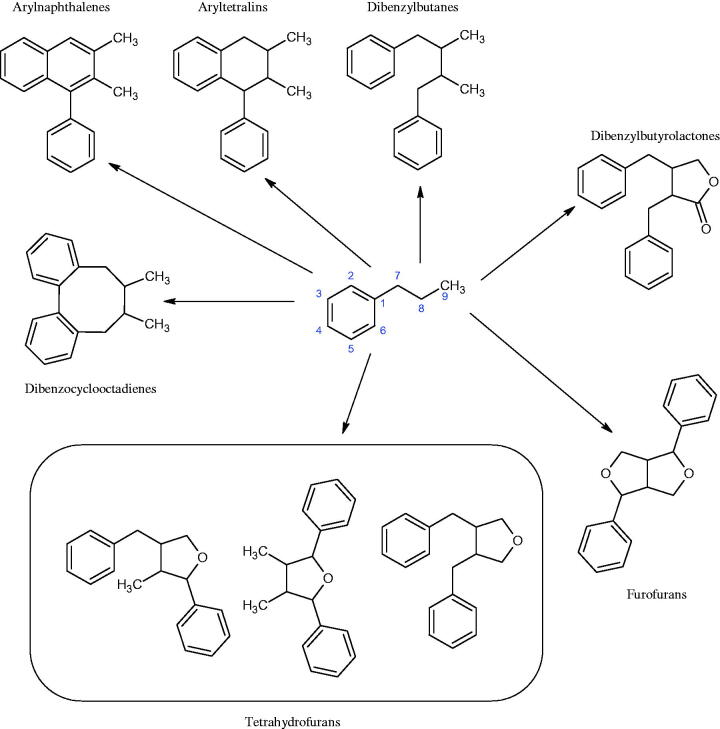
Chemical classification of lignans.

Lignans are phenylpropanoid dimers belonging to the group of plant phytoestrogens (Figure 18). These compounds are widespread in seeds (lentils), vegetables (garlic and asparagus), and fruits (pears and plums), however, the richest source is linseed and whole cereal grains. They are part of the cell wall and can be released by intestinal bacteria. Due to the similar structure to oestrogens, lignans compete for oestrogen receptors. In oestrogen deficiency, lignans gently complement their action, and when there is an excess of them, they reduce their activity because they have a much weaker oestrogenic effect. As a result, they help maintain the hormonal balance in the body and reduce the risk of various hormone-dependent diseases. Besides, these compounds protect against osteoporosis, lower LDL cholesterol, inhibit bacteria and fungi’ growth, and lower blood glucose levels. The most important compounds in this group are sesamine, sesaminol, sesamoline, pinoresinol, secoisolaricresinol, matairesinol, schizandrin, and schizandrol^115–120^.

Eight lignans were isolated from the methanolic extract of *Vitex negundo* L., i.e. negundin A, negundin B, 6-hydroxy-4-(4-hydroxy-3-methoxy)-3-hydroxymethyl-7-methoxy-3,4-dihydro-2-naphthalodehydrate, vitrofolal E, (+)-lyoniresinol, (+)-lyoniresinol-3α-O-β-D-glucoside, (+)-(-)-pinoresinol, and (+)-diasyringaresinol. The lactam ring present in negundin A caused moderately strong (${\mathrm{IC}}_{50}$=10.06 µM) inhibition of tyrosinase as compared to kojic acid (${\mathrm{IC}}_{50}$=16.67 µM). Negundin B, with the -CH2OH group in the C-2 position and the C = C bond between C-1 and C-2, showed stronger (${\mathrm{IC}}_{50}$=6.72 µM) inhibition of tyrosinase compared to kojic acid (${\mathrm{IC}}_{50}$=16.67 µM). Compound 3, in which the CH2OH group in the C-2 position was replaced with an aldehyde group, blocked tyrosinase to a lesser extent (${\mathrm{IC}}_{50}$=7.81 µM) than negundin B. Removal of the CH2OH group in the C-3 position and introduction of the C = C bond between C3 and C-4 reduced vitrofolal E’s strength (${\mathrm{IC}}_{50}$=9.76 µM). The strongest inhibitor was (+)-lyoniresinol, in which both positions C-2 and C-3 contain the CH2OH group (${\mathrm{IC}}_{50}$=3.21 µM). Glycosylation of (+)-lyoniresinol at position C-3 rendered inactive. The presence of a sugar residue hinders the interaction between the enzyme’s active site and the inhibitor. (+)-(-)-Pinoresinol showed moderate inhibition (${\mathrm{IC}}_{50}$=15.13 µM). The introduction of -OCH3 groups in the 5′ and 3′ positions in (+)-(-)-pinoresinol, lead to the formation (+)-diasyringaresinol which strongly inhibits tyrosinase (${\mathrm{IC}}_{50}$=5.61 µM). The compound (+)-lyoniresinol could be used as a potential lead molecule in bioprospecting^121^. Also, other lignans exhibited an anti-tyrosinase activity, e.g. 5,5-dimethoxylaryresinol-4-O-β-d-glucopyranoside and eleutheroside $E_{1}$ showed significant inhibition with the ${\mathrm{IC}}_{50}$ value of 42.1 and 28 µM, respectively^122^.

Lignan glycosides showed a moderate inhibitory effect on tyrosinase (4–5 times less than kojic acid) in the presence of L-DOPA as a substrate (4-O-lariciresinol-glucoside − 17.74% inhibition and 4′-O-lariciresinol-glucoside − 12.27% inhibition). When compared to lignan diglucoside (11.06% inhibition), they showed a lower activity, probably due to the complete absence of free hydroxyl groups (Table 17)^112^.

**Table 17.** An anti-tyrosinase activity of lignans.

Substances	Source of compounds	Substrate	Positive control (inhibitor)	Type of inhibition	Inhibition constant $K_{i}$ (mM)	Source of tyrosinase	The half-maximal inhibitory concentration ${\mathrm{IC}}_{50}$(µM)	References
Negundin A	*Vitex negundo*	L-DOPA	Kojic acid L-mimosine	NR	NR	Mushroom	10.06	Malik et al.^120^
Negundin B		L-DOPA		NR	NR	Mushroom	6.72	Malik et al.^120^
6-Hydroxy-4-(4-hydroxy-3-methoxy)-3-hydroxymethyl- 7-methoxy-3,4-dihydro-2-naphthaledehyde		L-DOPA		NR	NR	Mushroom	7.81	Malik et al.^120^
Vitrofolal E		L-DOPA		NR	NR	Mushroom	9.76	Malik et al.^120^
(+)-Lyoniresinol		L-DOPA		NR	NR	Mushroom	3.21	Malik et al.^120^
(+)-Lyoniresinol-3α-O-β-D-glucoside		L-DOPA		NR	NR	Mushroom	NA	Malik et al.^120^
(+)-(−)-Pinoresinol		L-DOPA		NR	NR	Mushroom	15.13	Malik et al.^120^
(+)-Diasyringaresinol		L-DOPA		NR	NR	Mushroom	5.61	Malik et al.^120^
Eleuterozyd $E_{1}$	*Opilia amentacea*	L-DOPA	Kojic acid	NR	NR	Mushroom	28	Magid et al.^121^
4-O-Lariciresinol-glucoside	*Marrubium velutinum and Marrubium cylleneum*	L-DOPA	Kojic acid	NR	NR	Mushroom	64	Myose et al.^78^
4′-O-Lariciresinol-glucoside		L-DOPA	Kojic acid	NR	NR	Mushroom	64	Myose et al.^78^
4,4′-O-Lariciresinol-bis-glucoside		L-DOPA	Kojic acid	NR	NR	Mushroom	49	Myose et al.^78^

NR: not reported; NA: not active

#### 5.2.4. Flavonolignans

Phytochemicals composed of part flavonoid and part phenylpropanoid, which are commonly found in nature. The richest source of these compounds is *Silybum marianum* from the *Asteraceae* family. They show hepatoprotective, anticancer, and anti-inflammatory effects^123^.

For example, isosilybin A (IC_50 _=2.1 µM) was more effective than its three mother compounds 3′-O-methyltaxifolin (IC_50 _=51.2 µM), dihydrokaempferol (IC_50 _=73.6 µM), and taxifolin (IC_50 _=23.0 µM). Analysing the structure of the mother compounds, deletion or methylation of the 3′-OH group 2.5–3 times reduces the activity of the compounds. Silychristin A (IC_50 _=3.2 µM; IC_50 _=28.8 µM) and silychristin B (IC_50 _=4.5 µM; IC_50 _=44.9 µM) having a double bond between C-2 and C-3 more potently inhibited tyrosinase activity than 2,3-dihydrosilychristin (IC_50 _=7.6 µM; IC_50 _=35.9 µM) having no double bond. The isolated compounds showed a mixed type of inhibition (Ki: L-tyr, 0.7–4.7 µM; L-DOPA, 8.5–36.7 µM). The mother compounds inhibited the enzyme in a competent manner (Table 18)^124^.

**Table 18.** An anti-tyrosinase activity of flavonolignans.

Structure	Name	IC_50_ (μM)	Ref.
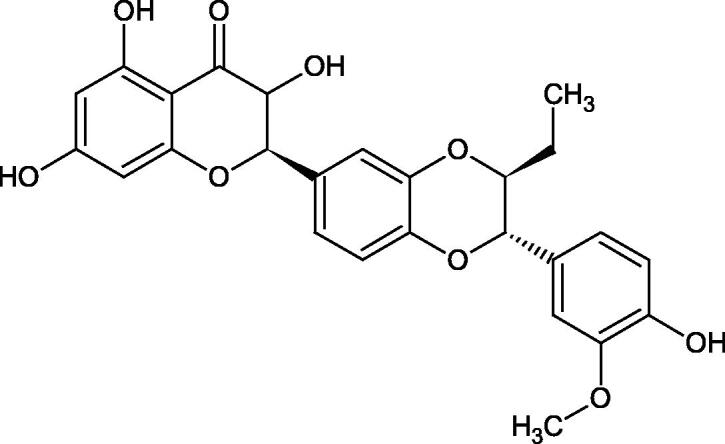	Isosilybin A	2.1^a^; 16.7^b^	Kim et al.^123^
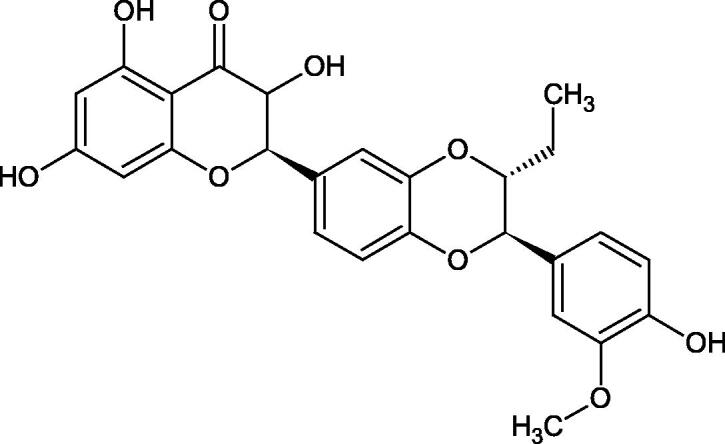	Isosilybin B	4.9^a^; 19.8^b^	Kim et al.^123^
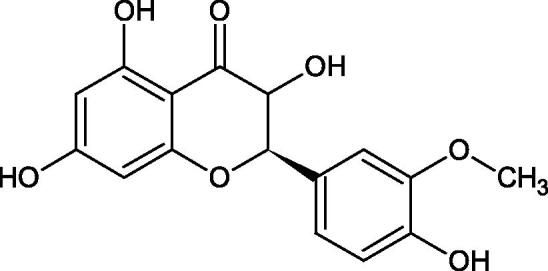	3′-O-Methyltaxifolin	51.2^a^; 150.0^b^	Kim et al.^123^
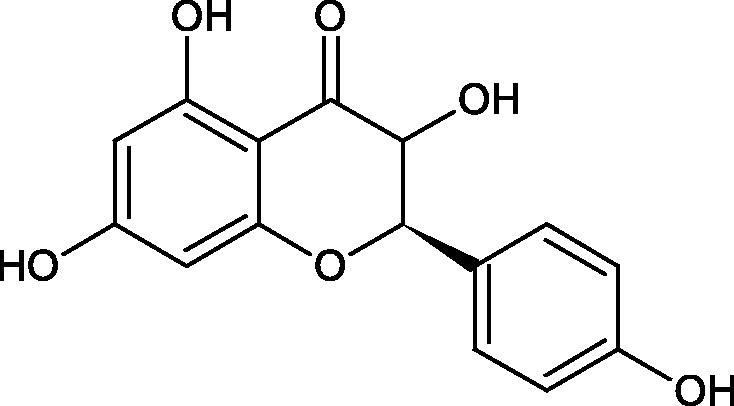	Dihydrokaempferol	73.6^a^; >200^b^	Kim et al.^123^
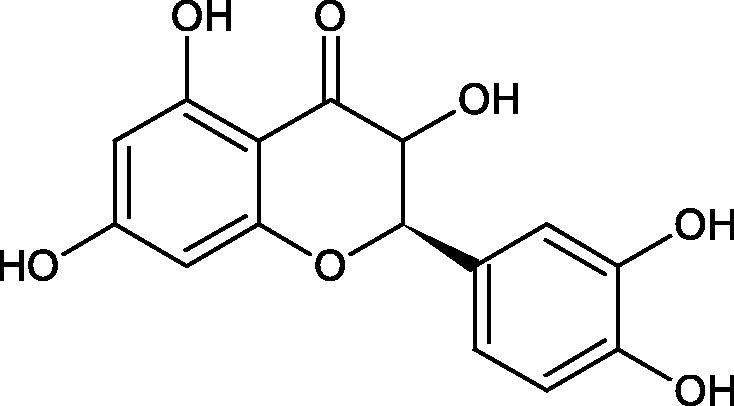	Taxifolin	23.0^a^; 27.0^b^	Kim et al.^123^
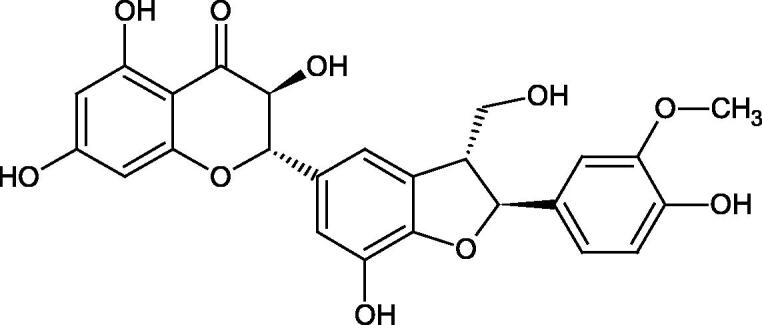	Silychristin A	3.2^a^; 28.8^b^	Kim et al.^123^
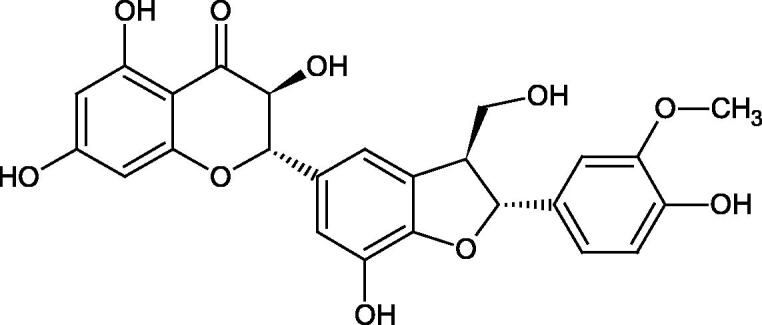	Silychristin B	4.5^a^; 44.9^b^	Kim et al.^123^
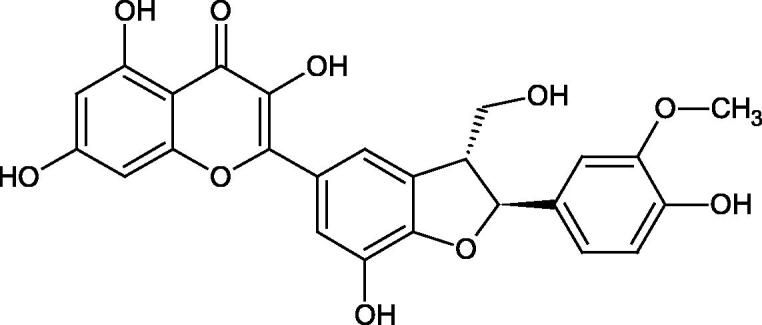	Dihydrosilychristin	7.6^a^; 35.9^b^	Kim et al.^123^

^a^L-tyrosine; ^b^L-DOPA.

#### 5.2.5. Stilbenes

These compounds belong to phytoalexins, low molecular weight cell components with antibacterial and antifungal properties. Besides, they show other biological properties such as antioxidant, anti-inflammatory, and antiproliferative effects. A characteristic feature of their structure is the presence of a 1,2-diphenylethylene core. More than 400 natural stilbenes have been discovered, but due to the low abundance of the critical enzyme stilbene synthase, they are not widely distributed in nature. The primary source of stilbenes in the human diet are grapes, red wine, and peanuts. The most famous representative of this group is resveratrol^125^.

##### 5.2.5.1. Hydroxyl groups

Many naturally occurring stilbenes exhibit tyrosinase inhibitory activity, that is related to the characteristic elements of their structure. The inhibitory properties are due to the number and distribution of oxygen atoms attached to the aromatic rings. Dioxyl stilbene, pinosylvin, showed weak inhibitory properties (${\mathrm{IC}}_{50}\;$=46 µM), while resveratrol, a stilbene representative with three hydroxyl groups, inhibited tyrosinase even more strongly than kojic acid. However, when compared to oxyresveratrol, representative of tetroxyl stilbenes, that compound showed a nine-fold increase in the inhibition than resveratrol (${\mathrm{IC}}_{50}$=1.5 *vs.* 14.4 µM)^126^. To better understand, the structure–activity relationship in a model hydroxystilbene-tyrosinase new derivatives of trans-stilbene were synthesised^127^. Monohydroxy trans stilbenes showed no inhibitory effect on tyrosinase, only after attachment of another hydroxyl group to the aromatic ring resulted in an increase of inhibition. The braking force depended on the position of the hydroxyl groups to each other, e.g. 3,3′-dihydroxy-transstilbene (26.3% inhibition ${\mathrm{IC}}_{50\;}\;$>200 µM) has a more substantial inhibitory effect than 2,3-dihydroxy-trans-stilbene (4.4% inhibition ${\mathrm{IC}}_{50}$>200 µM) and 3,4-dihydroxy-trans-stilbene (9.5% inhibition ${\mathrm{IC}}_{50}$>200 µM) or 3,5-dihydroxy-trans-stilbene (18.6% inhibition ${\mathrm{IC}}_{50\;}$>200 µM). The 3,3′,4-trihydroxy-trans-stilbene (87.7% inhibition ${\mathrm{IC}}_{50\;}$=74.3 µM) and 3,3′,4,4′-tetrahydroxy-trans-stilbene (98.3% inhibition ${\mathrm{IC}}_{50}\;$=29.1 µM) showed more potent activity against the enzyme than 3,3′-dihydroxy-trans-stilbene. 3,3,4,4′-tetrahydroxy-trans-stilbene (${\mathrm{IC}}_{50}\;$=29.1 µM) inhibited the tyrosinase activity almost completely. It is seen that an increase of the inhibitory power of the hydroxystilbenes is correlated with an increase of the number of hydroxyl groups. O-methylation decreased the action of the stilbenes.

##### 5.2.5.2. Stilbene glycosides

Other studies have examined the difference in an action between stilbene glycosides and their aglycones. Several hydroxystilbenes were isolated from the methanolic extract of *Veratrum patulum* L. (${IC}_{50}$=100 µM), including piceid, the aglycone of which is resveratrol. The inhibitory activity of piceid was 6.9 and 8.2 (L-DOPA and L-tyrosine) times lower than that of resveratrol (phenylthiourea was used as a positive control). The tested compounds showed a more significant effect on the monophenolase activity than on the diphenolase activity^128^. Kim et al. who studied the impact of mulberroside A (isolated from the ethanolic *Morus alba* L. root extract) enzymatic biotransformation to oxyresveratrol and their anti-tyrosinase activity. The inhibitory activity of oxyresveratrol was approximately 110-fold higher than that of mulberroside A (${\mathrm{IC}}_{50}\;$= 0.49 and 53.6 µM, respectively). Kojic acid and arbutin were selected as controls in the study. Inhibition of tyrosinase activity by oxyresveratrol (L-tyrosine) was 43-fold and 1503-fold higher than that of kojic acid and arbutin (${\mathrm{IC}}_{50\;}$=21.1 and 736.5 µM, respectively). Tyrosinase was almost completely inhibited by oxyresveratrol, mulberroside A, and kojic at concentrations of 2.5, 500, and 250 µM, respectively. Arbutin weakly inhibited tyrosinase (about 85%, 3000 µM). On the basis of the kinetic parameters, it has been shown that mulberroside A is a competitive inhibitor of fungal tyrosinase with L-tyrosine and L-DOPA as a substrate, oxyresveratrol showed mixed inhibition and non-competitive inhibition to L-tyrosine and L-DOPA as the substrate, respectively. Tyrosinase catalyses two different reactions: the hydroxylation of monophenols to o-diphenols (monophenolase activity) and the oxidation of o-diphenols to o-quinones (diphenolase activity). Considering the ${IC}_{50}$(L-tyrosine 0.49; L-DOPA 11.9) and Ki (L-Tyrosine 1.093, 0.521; L-DOPA 1.272) values, oxyresveratrol had a more significant impact on the activity of monophenolase than on the diphenolase activity^129^.

Isolated from the water-methanol extract of the rhizome of *Rheum officinale* Baill galloyl glucosides of resveratrol, e.g. 3,4′, 5-trihydroxystilbene-4′-O-β-D- (2′′-O-galloyl) glucopyranoside (A) and 3,4′, 5-trihydroxystilbene-4′-O-β-D- (6″ -O-Galloyl) glucopyranoside (B) inhibit the activity of tyrosinase. These compounds showed a competitive type of inhibition and blocked the enzyme stronger than kojic acid. The compounds inhibited the conversion of L-tyrosine to L-DOPA more strongly than L-DOPA to DOPA quinone. Inhibitory effect for compounds A (${\mathrm{IC}}_{50}\;$=6.71 µM) and B (${\mathrm{IC}}_{50\;}$=14.7 µM) was higher than for kojic acid (${\mathrm{IC}}_{50\;}$=28.9 µM), when L-tyrosine was used as a substrate. Inhibitory effect of compound B (${\mathrm{IC}}_{50}\;$=82.3 µM) was significantly less than that of kojic acid (${\mathrm{IC}}_{50}\;$=23 µM),when L-DOPA was used as a substrate. In the case of compound A the ${\mathrm{IC}}_{50}$ value was comparable to that of kojic acid (${\mathrm{IC}}_{50\;}$=24.6 µM)^130^.

The obtained results indicate that the deglycosylation of stilbenes has a positive influence on their activity and indicates that the aglycones are more active. This is probably related to particle size because as larger compounds, glycosides have restricted an access to the active site of the enzyme.

##### 5.2.5.3. Isoprenyl chain

The inhibition of tyrosinase may also be stimulated by the compounds with isoprenyl chain in their structure, e.g. 4-[(2′′E)-7′′-hydroxy-3′’,7′′-dimethyloct-2′′-enyl] − 2′,3,4′,5-tetrahydroxy-trans-stilbene (compound A) and chlorophorin, both isolated from *Chlorophora excels* (Welw.) Benth. core, showed a different inhibitory activity dependent on the presence of the isoprenyl chain. The ${\mathrm{IC}}_{50}$ values for compound A and chlorophorin were equal 96 and 1.3 µM, respectively (kojic acid, ${\mathrm{IC}}_{50}\;$=20 µM). Attaching a water molecule to the geranyl chain in compound A reduced its tyrosinase activity. This is probably due to reducing the chain’s interaction and the hydrophobic protein pocket close to the active site^131^. The other investigations have shown that the presence of a prenyl chain in a compound having a 4-substituted resorcinol backbone increased the inhibitory activity compared to that of oxyresveratrol (${\mathrm{IC}}_{50}$=0.66 and 0.98 µM, respectively).

In another study, the impact of chain length, functional groups (polarity), and cyclisation of isoprenyl chains on the anti-tyrosinase activity was investigated. An increase in chain length from one isoprene unit to two resulted in a 4-fold increase in activity (IC50 = 15.87 µM, IC50 = 60.14 µM). In turn, the introduction of hydroxyl groups or cyclisation of the isoprenyl chain caused a drastic decrease in anti-tyrosinase activity. Similar results of isoprenyl chain influence were observed in stilbene derivatives isolated from *Angelica keiskei* roots.

##### 5.2.5.4. Double bond

**Figure 19.**
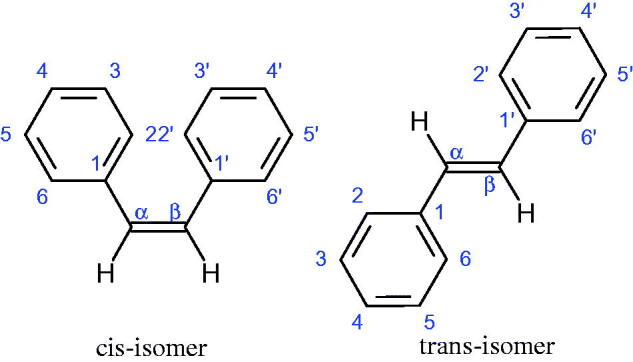
Chemical structure of *cis*- and *trans*-isomer of stilbenes.

Another compound inhibiting tyrosinase is gnetol, a tetrahydroxystilbene isolated from *Gnetum gnemon* L. It was appeared that gnetol is approximately 30 times more potent than kojic acid. Additionally, gnetol inhibited tyrosinase much more than dihydrognetol (100% and 20%, respectively). The double bond is crucial for the activity. The double bond’s role in the stilbene backbone in inhibiting tyrosinase examined the cis-olefin structure. The cis isomer of 3,3′-dihydroxystilbene (% inhibition c. 1) compared to the trans isomer (26% inhibition) shows no inhibitory effect. The saturation of the double bond in the oxyresveratrol (${\mathrm{IC}}_{50}\;$=0.98 µM) significantly reduces the activity (${\mathrm{IC}}_{50\;}$=58 µM). However, dihydrooxyresveratrol showed eight times more inhibitory effect on the activity of fungal tyrosinase than oxyresveratrol (${\mathrm{IC}}_{50}\;$=1.6 and 12.7 µM, respectively). The higher activity of dihydrooxyresveratrol, compared to oxyresveratrol, was probably due to its dibenzyl structure, which provided greater flexibility, and thus allowed for a more effective interaction of phenolic groups with the enzyme (Figure 19)^132^.

Similar results of the effect of double bond saturation were obtained by studying dihydrostilbene derivatives isolated from the 80% ethanolic extract of the *Dendrobium loddigesii* Rolfe stem. It was appeared that 3,4,5-trihydroxy-3′,4′-dihyroxyhydrostilbene; 3,5-dihydroxy-3′,4′-dihyroxyhydrostilbene and their methoxy derivatives did not inhibit tyrosinase activity. The exception to this rule is 3,5-dihydroxy-3′-dihyroxyhydrostilbene (IC_50_ = 37.90 µM). Attachment of dioxolane (aphyllals C) to the B ring of 3,5-dihyroxyhydrostilbene results in a marked increase in an activity (IC_50 _=152.56 µM). Methylation of the 3-OH group in aphyllals C abolishes the compound’s activity. The above examples indicate that methylation of the 3-OH group inactivates the compound. Kojic acid was used as a positive control (IC_50 _=8.02 µM). A noteworthy compound for (Q)SAR studies is 1,3-benzodioxol derivative (benzene ring linked to dioxolane). Due to the ambiguity of the results, further studies on the effect of a double bond in stilbenes are needed.

##### 5.2.5.5. Stilbene oligomers

**Figure 20.**
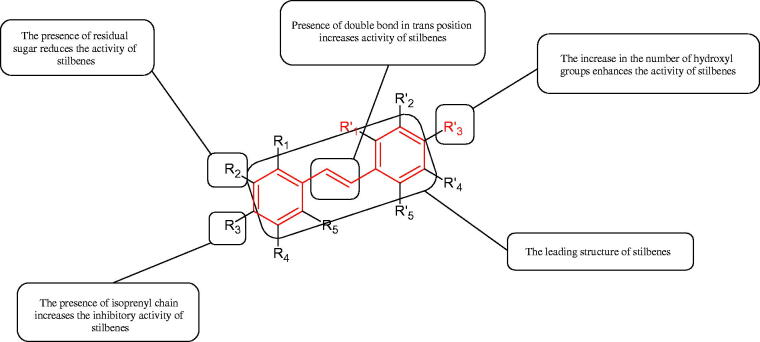
Potential groups engaged in an interaction stilbene-tyrosinase.

The next group with an anti-tyrosinase activity is oligomers of stilbenes. The following resveratrol oligomers: ε-viniferin as a dimer; vaticanol A, vaticanol G, and α-viniferin as trimers; vaticanol B, vaticanol C and (-)-hopeaphenol as tetramers were tested towards tyrosinase from murine B16 melanoma cells and L-DOPA as the substrate and kojic acid as a control (${\mathrm{IC}}_{50}\;$=119.7 µM; c. 49.3% inhibition). Resveratrol (${\mathrm{IC}}_{50\;}$=10.8 µM) at a concentration of 100 µM inhibited the activity of tyrosinase at the level of 98%. However, the oligomers have appeared to be weak inhibitors, e.g. dimer-ε-viniferin at a concentration of 100 µM showed approx. 24.6% inhibition, the trimmers, and tetrameters showed an inhibition level of less than 8%. It may be suggested that the inhibitory potency of the resveratrol oligomers decreases with increasing molecular weight (Figure 20; Tables 4S and Table 19)^133^.

**Table 19.** An anti-tyrosinase activity of stilbenes.

Substances	Source of compounds	Substrate	Positive control (inhibitor)	Type of inhibition	Inhibition constant $K_{i}$	Source of tyrosinase	The half-maximal inhibitory concentration ${\mathrm{IC}}_{50}$ (µM)	References
Pinosylvin	Gnetum cleistostachyum	L-tyrosine	Kojic acid	${\mathrm{Competitive}}^{\mathrm{ab}}$	NR	Mushroom	46.0	Likhitwitayawuid^125^
Resveratrol-4′-O-(6′’-galloyl)glucoside	Rheum officinale	L-tyrosine L-DOPA	Kojic acid	${\mathrm{Competitive}}^{\mathrm{ab}}$	NR	Mushroom	$6.71^{a}\;$ $24.6^{b}$	Iida et al.^129^
Resveratrol-4-O-(6″-O-galloyl)glucoside		L-tyrosine L-DOPA	Kojic acid	${\mathrm{Competitive}}^{\mathrm{ab}}$	NR	Mushroom	$14.7^{a}\;$ $82.3^{b}$	Iida et al.^129^
Mulberroside A	Morus alba	L-tyrosine L-DOPA	Kojic acid, arbutin	${\mathrm{Competitive}}^{\mathrm{ab}}$	49.82^a^ 124.764^b^	Mushroom	$53.6^{a}$	Kim et al.^128^
Oxyresveratrol		L-tyrosine L-DOPA	Kojic acid, arbutin	Competitive – non-competitive^a^ Non-competitive^b^	1.093^a^ 0.521^b^	Mushroom	$0.49^{a}$ $11.9^{b}$	Kim et al.^128^
Resveratrol	Veratrum patulum	L-tyrosine L-DOPA	Kojic acid	NR	NR	Mushroom	43.5 123.3	Kim et al.^127^
Resveratrol	Dipterocarpaceae plants	L-DOPA	Kojic acid	NR	NR	Murine	10.8	Ohguchi et al.^131^
Piceid	Veratrum patulum	L-tyrosine L-DOPA	Phenylthiourea	NR	NR	Mushroom	${>100}^{a}$ ${>\;500}^{b}$	Kim et al.^127^
Piceid	Dipterocarpaceae plants		Kojic acid	NR	NR	Murine	71.3	Ohguchi et al.^131^
Pinostilben (3,5-Dihydroxy-4′- methoxystilbene)	Veratrum patulum	L-tyrosine L-DOPA	Kojic acid	NR	NR	Mushroom	$86.8^{a}$ $187.9^{b}$	Kim et al.^127^
Pterostilbene (3,4′-Dimethoxy-5- hydroxystilbene)		L-tyrosine L-DOPA	Kojic acid	NR	NR	Mushroom	${>100}^{a}$ $398.3^{b}$	Kim et al.^127^
Rhaponticin		L-tyrosine L-DOPA	Kojic acid	NR	NR	Mushroom	${>100}^{a}$ ${>500}^{b}$	Kim et al.^127^
Chlorophorin	Chlorophora excelsa	L-DOPA	Kojic acid	Competitive	NR	Mushroom	1.3	Shimizu et al.^130^
Chlorophorin	Artocarpus incisus	L-tyrosine	Kojic acid	${\mathrm{Competitive}}^{b}$	${>13.4}^{b}$	Mushroom	0.26	Shimizu et al.^130^
4-Prenyloxyresveratrol		L-tyrosine	Kojic acid	${\mathrm{Competitive}}^{b}$	$8.7^{b}$	Mushroom	0.66	Shimizu et al.^130^
4-[(2”E)-7”-Hydroxy-3”,7”-dimethyloct-2”- enyl]-2′,3,4′, 5-tetrahydroxy-trans-stilbene	Chlorophora excelsa	L-DOPA	Kojic acid	NR	NR	Mushroom	96.0	Shimizu et al.^130^
Gnetol	Gnetum gnemon	L-DOPA	Kojic acid	NR	NR	Murine		Ohguchi et al.^126^
Dihydrooxyresveratrol		L-tyrosine	Kojic acid	Competitive		Mushroom	58.0	Shimizu et al.^130^
Vaticanol A	Dipterocarpaceae plants	L-DOPA	Kojic acid	NR	NR	Murine	NR	Ohguchi et al.^131^
Vaticanol G		L-DOPA	Kojic acid	NR	NR	Murine	NR	Ohguchi et al.^131^
α-Viniferin		L-DOPA	Kojic acid	NR	NR	Murine	NR	Ohguchi et al.^131^
Vaticanol B		L-DOPA	Kojic acid	NR	NR	Murine	NR	Ohguchi et al.^131^
Vaticanol C		L-DOPA	Kojic acid	NR	NR	Murine	NR	Ohguchi et al.^131^
(−)-Hopeaphenol		L-DOPA	Kojic acid	NR	NR	Murine	NR	Ohguchi et al.^131^

^a^L-tyrosine; ^b^L-DOPA; NR: not reported.

#### 5.2.6. Chalcones

Chalcones belong to the group of unsaturated aromatic ketones and are thought to be the precursors for the synthesis of flavonoids. The core of chalcones consists of two aromatic rings linked by a three-carbon α, β-unsaturated carbonyl system (1,3-diphenyl-2-propen-1-one) (Figure 17). Chalcones can exist in trans (E) and cis (Z) forms, but cis isomers are unstable due to spherical effects. Chalcones are characterised by a broad spectrum of biological properties, including anticancer, antioxidant, antidiabetic, anti-inflammatory, antibacterial, and antiviral activities^133^.

Chalcones have shown an anti-tyrosinase activity dependent on their structure and concentration. Khatib et al. studied the effect of catechol and resorcinol groupings in the A and B rings of chalcones. Two substrates for tyrosinase, L-tyrosine (first step – L-tyrosine to L-DOPA) and L-DOPA (second step – L-DOPA to o-quinone), were used. All of the compounds tested showed greater activity in blocking the first step than the second step. 3,4,2′,4′- Hydroxychalcone, with a resorcinol moiety (at the 2′ and 4′ positions) in the A ring and catechol in the B ring inhibited the first step more potently (${\mathrm{IC}}_{50}$=29.3 µM) than the second step (${\mathrm{IC}}_{50}$>100 µM). 2,4,3′,4′- Hydroxychalcone, with the opposite structure to 3,4,2′,4′- hydroxychalcone, inhibited tyrosinase about 146.5-fold more potently (${\mathrm{IC}}_{50}$=0.2 µM – stage 1, and ${\mathrm{IC}}_{50\;}$=7.5 µM stage 2). The compound 3,5,2′,4′- hydroxychalcone, in which the OH groups of the B ring are located at positions 3 and 5 while maintaining the identical position of the catechol group as in 3,4,2′,4′- hydroxychalcone, blocked the enzyme weaker (${\mathrm{IC}}_{50}$=31.7 µM for step 1, ${\mathrm{IC}}_{50\;}$>1000 µM for step 2). Compound 2,4,2′,4′- hydroxychalcone, made up of two resorcinol groups in rings A and B, blocked tyrosinase activity most strongly (${\mathrm{IC}}_{50}$=0.02 µM for stage 1 and up to 90 µM for Stage 2). Additionally, 2,4,3′,4′- hydroxychalcone is 7.5 times more active than trans-stilbene, with the same catechol and resorcinol arrangement. Moreover, the position of OH groups in the A and B ring affects the mechanism of chalcone inhibition. Compounds with a catechol group showed the ability to chelate copper ions, while 2,4,2′,4′- hydroxychalcone (resorcinol structure) did not chelate copper ions. The catechol group in ring A acted as a chelator of copper ions, while the catechol in ring B is oxidised to o-quinone. However, catechol groups in the A or B ring had no significant impact on tyrosinase inhibition. The compound with two resorcinol moieties has the most potent effect on tyrosinase activity^134^.

A promising group of tyrosinase inhibitors may be 2′,4′,6′-trihydroxychalcone derivatives^135^. It was confirmed, that the absence of OH groups at the 4′ or 6′ position in 2′,4′,6′-trihydroxychalcones results in loss of an inhibitory activity (less than 20% inhibition at 400 µM concentration). Comparing the 2′,4′,6′-trihydroxychalcone activity to kojic acid, chalcone has exhibited a 10-fold weaker inhibition (${\mathrm{IC}}_{50}$=120 and 12 µM, respectively). Methoxylation of hydroxyl groups at the 4′ and 6′ position of compounds: 2,2′,3,4′,6′-trihydroxychalcones, 2′,3,4,4′,5,6′-trihydroxychalcones, and 2′,3,4,4′,6′-trihydroxychalcones also cause loss of their activity. 2′,2,4,4′,6′-Trihydroxychalcones (${\mathrm{IC}}_{50}$=1 µM) were found to exhibit the highest activity, even better than 2,2′,4,4′-tetrahydroxychalcone (${\mathrm{IC}}_{50}\;$=5 µM) and kojic acid (${\mathrm{IC}}_{50}$=12 µM). In contrast, methoxylation of the 6′-OH group in 2′,2,4,4′,6′-trihydroxychalcones impairs activity (${\mathrm{IC}}_{50}$=3.1 µM).

**Figure 21.**
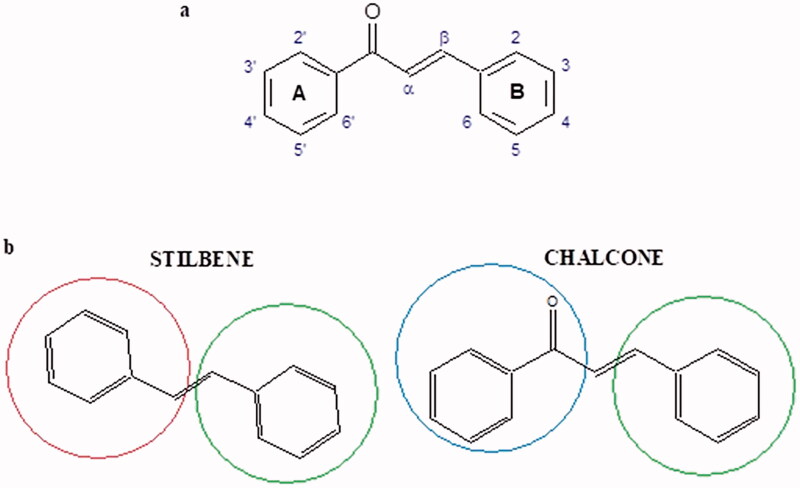
a) A basic structure of chalcones (1,3-diphenyl-2-propen-1-one); b) The difference in structure between stilbenes and chalcones.

Nguyen et al. provided more information about the influence of the position and number of OH groups on the activity of chalcones. It was appeared that a presence one or two hydroxyl groups in the A ring do not strength the inhibition, e.g. inhibition for 4-hydroxychalcone, 2-hydroxychalcone, and 2,4-dihydroxychalcone was about 14% at 50 µM, respectively. However, the changes had been observed after the introduction of the 4′-OH group in the B ring, which resulted in the activity’s increase. The inhibition at 50 µM for 4′-hydroxychalcone, 2,4,4′-trihydroxychalcone was 71% and 67%, respectively. Additionally, the presence of the 2-OH group in the A ring drastically reduces the activity of 2,4′-dihydroxychalcone (10% inhibition at 50 µM concentration). The weakening effect of the 4′-OH group on tyrosinase activity is due to conformational changes. These changes are due to the formation of hydrogen bonds between 2-OH and the carbonyl group. The importance of the arrangement of hydroxyl groups is related to the structure of chalcones. Ring A is associated with a carbonyl carbon atom, while ring B is related to a vinyl carbon atom. This is in contrast to stilbenes, in which the rings are bound to identical carbon atoms (Figure 21)^136^.

**Figure 22.**
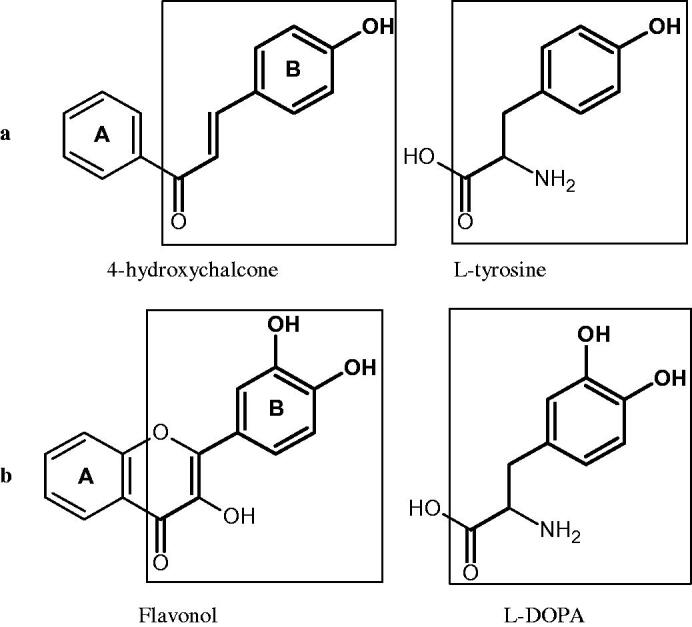
a) Similarity in the structure of L-tyrosine and 4-hydroxychalcones; b) Similarity in structure of L-DOPA and flavonol.

The anti-tyrosinase properties have also been confirmed in the group of the chalcone glycosides, e.g. licuraside, isoliquiritin, and the aglycone licochalcone A isolated from two species of *Glycyrrhiza* (*Glycyrrhiza uralensis* Fisch and *Glycyrrhiza inflate* Bat., respectively). The ${\mathrm{IC}}_{50}$ for licuraside, isoliquiritin, and licochalcone A, in the presence of L-tyrosine, were of 72, 38, and 25.8 µM, respectively. The compounds inhibited enzyme competitive (L-tyrosine). The inhibitory effect of chalcones on diphenolase activity (L-DOPA as substrate) was much lower. The difference in inhibition between monophenolase and diphenolase activity is related to the structure of the chalcones, those ones which inhibit monophenolase more strongly show a similarity to L-tyrosine (Figure 22)^134^.

The 4-OH group in the B ring of chalcones affects the potency of the inhibitor (similarity to the tyrosine backbone). Licochalcone A is a more potent inhibitor than licurad and isoliquiritin because it has a free 4-OH group in the B ring and has less steric hindrance. The sugar residue at the 4′-OH position hinders access to the enzyme’s active centre, resulting in reduced inhibitory activity. In addition, the 3,3- dimethylpropylene group at position 5 (ring B) of licochalcone A disrupts the quaternary structure of tyrosinase, inhibiting the enzyme^134^.

**Figure 23.**
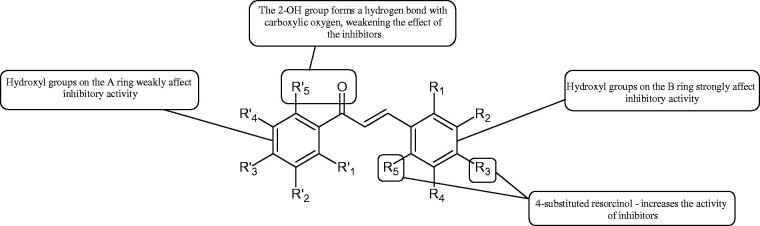
Potential groups engaged in an interaction chalcone-tyrosinase.

In the case of diphenolase activity, for which the substrate is L-DOPA, the presence of both 3′-OH and 4′-OH groups in the B ring is necessary in the inhibitor’s structure in order to resemble L-DOPA. The absence of the 3′-OH group in the B ring of chalcones resulted in the lack of diphenolase inhibitory activity of tyrosinase^137^. The tyrosinase activity may be also regulated *via* the introduction of alkyl chains into chalcone molecules. It has been proved that prenylated chalcone, curaridine, blocked the enzyme activity very strongly (${\mathrm{IC}}_{50}$=0.6 µM) compared to kojic acid (${\mathrm{IC}}_{50}$=20.5 µM). The important elements of the studied compound are the 2′-OH and 4′-OH groups in the B ring, and 4-OH, and the prenyl chain at C-5 (lavandulyl) in the A ring^138^. On the other hand, the addition of two isoprenyl groups at the C-5 and C-3′ positions to 4,4′,6-trihydroxychalcone abolishes tyrosinase inhibition. This is probably due to steric hindrance (Figure 23; Tables 5S and Table 20)^134^.

**Table 20.** An anti-tyrosinase activity of chalcones.

Substances	Substrate	Positive control (inhibitor)	Type of inhibition	Inhibition constant $K_{i}$	Source of tyrosinase	The half-maximal inhibitory concentration ${\mathrm{IC}}_{50}\;$(µM)	References
3,4,2′,4′-Hydroxychalcone	L-tyrosine L-DOPA	Kojic acid	NR	NR	Mushroom	$29.3^{a}/{>100}^{b}$	Khatib et al.^133^
2,4,3′,4′-Hydroxychalcone		Kojic acid	NR	NR	Mushroom	$0.2^{a}/{>7.5}^{b}$	Khatib et al.^133^
3,4,2′,4′-Hydroxychalcone		Kojic acid	NR	NR	Mushroom	$31.68^{a}/{>1000}^{b}$	Khatib et al.^133^
2,4,2′,4′-Hydroxychalcone		Kojic acid	NR	NR	Mushroom	$0.02^{a}/90^{b}$	Khatib et al.^133^
2′,4′,6′-Trihydroxychalcone	L-tyrosine	Kojic acid	NR	NR	Mushroom	120	Jun et al.^134^
2′,4′-Dihydroxychalcone		Kojic acid	NR	NR	Mushroom	NA	Jun et al.^134^
2′,6′-Dihydroxychalcone		Kojic acid	NR	NR	Mushroom	NA	Jun et al.^134^
2′,3,4,4′,6′-Pentahydroxychalcone		Kojic acid	NR	NR	Mushroom	193	Jun et al.^134^
2′,3,4,4′,5,6′-Hexahydroxychalcone		Kojic acid	NR	NR	Mushroom	200	Jun et al.^134^
2′,3,4,4′,6′-Pentahydroxychalcone		Kojic acid	NR	NR	Mushroom	NA	Jun et al.^134^
3,3′,4,4′-Tetrahydroxychalcone		Kojic acid	NR	NR	Mushroom	NA	Jun et al.^134^
2′,3,4-Trihydroxychalcone		Kojic acid	NR	NR	Mushroom	NA	Jun et al.^134^
2′,4′,6′-Trihydroxy-3,4-dimethoxychalcone		Kojic acid	NR	NR	Mushroom	150	Jun et al.^134^
2,2′,3-Trihydroxy-4′,6′-dimethoxychalcone		Kojic acid	NR	NR	Mushroom	NA	Jun et al.^134^
2′,3,4,5,-Tetrahydroxy-4′,6′-dimethoxychalcone		Kojic acid	NR	NR	Mushroom	NA	Jun et al.^134^
2′,3,4-Trihydroxy-4′,6′-dimethoxychalcone		Kojic acid	NR	NR	Mushroom	NA	Jun et al.^134^
2,2′,4,4′-Tetrahydroxychalcone		Kojic acid	NR	NR	Mushroom	5	Jun et al.^134^
2,2′,4,4′-Tetrahydroxy-6′-methoxychalcone		Kojic acid	NR	NR	Mushroom	3.1	Jun et al.^134^
2,2′,4,4′,6′-Pentahydroxychalcone		Kojic acid	Competitive	3.1	Mushroom	1	Jun et al.^134^
Licochalcone A	L-DOPA	Kojic acid	Competitive	NR	Mushroom	25.8	Fu et al.^136^
Licuraside		Kojic acid	Competitive	NR	Mushroom	72	Fu et al.^136^
Isoliquiritin		Kojic acid	Competitive	NR	Mushroom	38	Fu et al.^136^

^a^L-tyrosine; ^b^L-DOPA; NR: non reported; NA: non active

#### 5.2.7. Phenylpropanoid sucrose esters (PSEs)

**Figure 24.**
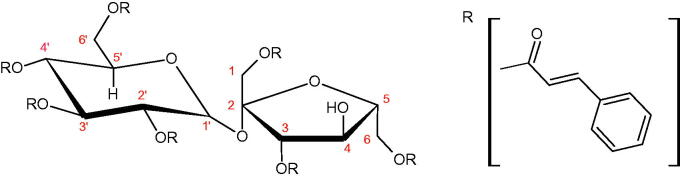
Structure of Phenlpropanoid Sucrose Esters – PSEs (R: phenylpropanoid residues).

Phenylpropanoid sucrose esters (PSEs) are composed of a sucrose core linked to one or more phenylpropanoid residues (Ph-CH = CH-CO-) *via* an ester bond. PSEs include substituted/unsubstituted caffeic, coumaric, ferulic, cinnamic, and sinapic acids. PSEs have been isolated from various species of medicinal plants in the families *Arecaceae*, *Boraginaceae*, *Brassicaceae*, *Caryophyllaceae*, *Liliaceae*, *Melanthiaceae*, *Polygonaceae*, *Poaceae*, *Polygalaceae*, *Rutaceae*, and *Rosaceae*. These compounds exhibit anti-inflammatory, antioxidant, hypoglycaemic, and anticancer activities (Figure 24)^139–141^.

The PSEs isolated from *Persicaria orientalis* (L.) Spach showed low to medium tyrosinase inhibitory abilities [hydropiperoside (L-tyr, IC_50 _=27.1 µM; L-DOPA, IC_50 _=166.15 µM), vanicoside A (L-tyr, IC_50 _=37.29 µM; L-DOPA, IC_50 _=135.91 µM), vanicoside B (L-tyr, IC_50 _=62. 0 µM; L-DOPA, IC_50 _=113.13 µM), vanicoside C (L-tyr, IC_50 _=39.0 µM; L-DOPA, IC_50 _=91.38 µM), and vanicoside E (L-tyr, IC_50 _=45.23 µM; L-DOPA, IC_50 _=189.96 µM)]. In this study, kojic acid (L-tyr, IC_50 _=14.15 µM; L-DOPA, IC_50 _=181.40 µM) was used as a positive control^142^. Cho et al. investigated the inhibitory effect of PSEs isolated from the *Oryza sativa* roots on tyrosinase activity. The most active compounds were 3,6-diferuloyl-3′,6′-diacetylsucrose (IC_50 _=47.33 µM) and smilaside A (IC_50_ =45.13 µM). The IC_50_ for 3-feruloyl-4′,6′-diacetyl sucrose, 3-feruloyl-6′-acetylsucrose 3,6-diferuloylsucrose was >400 µM. None of the isolated compounds inhibited the enzyme more strongly than the positive control, kojic acid (IC_50 _=28.60 µM)^143^.

Summarising results of the above studies, they indicate the significance of the presence of a feruloyl group at C-6, an acetyl group at C-6′, and another acetyl group at C-3′/C-4′ in inhibiting of the enzyme. Additionally, it seems that the introduction of additional feruloyl groups in the fructose moiety increases the activity of the compounds (Table 21).

**Table 21.** Structure and an anti-tyrosinase activity of phenylpropanoid sucrose esters.

Structure	Name	*R*_1_		*R*_2_	*R*_3_	IC_50_ (μM)	Ref.
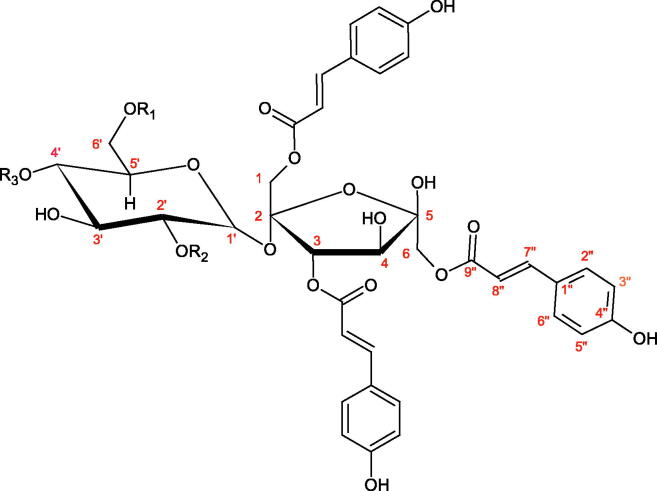	Hydropiperoside	H		H	H	27.1^a^/166.15^b^	Masum et al.^141^
	Vanicoside A	feruloyl		Ac	H	37.29^a^/135.91^b^	Masum et al.^141^
	Vanicoside B	feruloyl		H	H	62.0^a^/113.13^b^	Masum et al.^141^
	Vanicoside C	H		Ac	H	39.0^a^/91.38^b^	Masum et al.^141^
	Vanicoside E	feruloyl		Ac	Ac	45.23^a^/181.40^b^	Masum et al.^141^
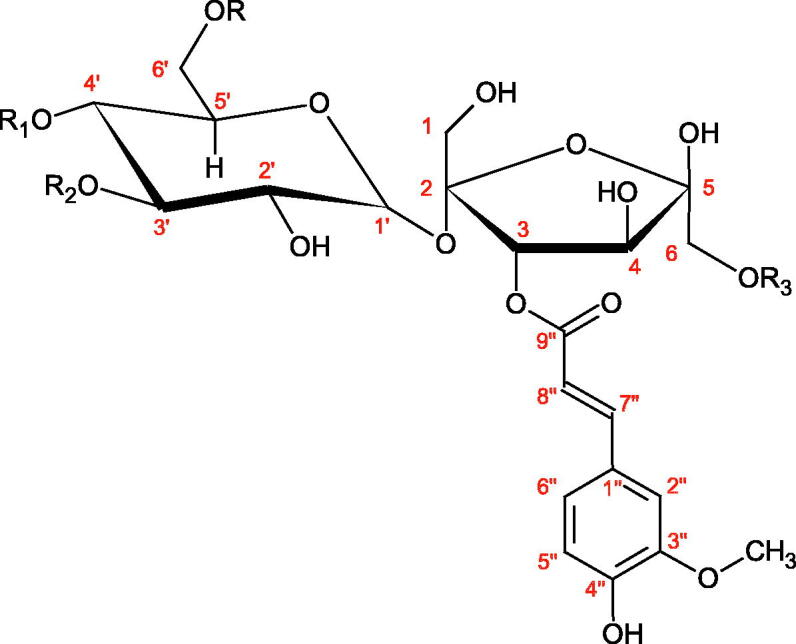	3-Feruloyl-4′,6′-diacetyl sucrose	Ac	Ac	H	H	>400	
	3,6-Diferuloyl-3′,6″-diacetylsucrose	Ac	H	Ac	feruloyl	47.33	Cho et al.^142^
	Smilaside A	Ac	Ac	H	feruloyl	45.13	Cho et al.^142^
	3,6-Diferuloyl-6′-acetylsucrose	Ac	H	H	feruloyl	372.6	Cho et al.^142^
	3-Feruloyl-6′-acetylsucrose	Ac	H	H	H	>400	Cho et al.^142^
	3,6-Diferuloylsucrose	H	H	H	feruloyl	>400	Cho et al.^142^

^a^L-tyrosine; ^b^L-DOPA.

### 5.3. Coumarin

**Figure 25.**
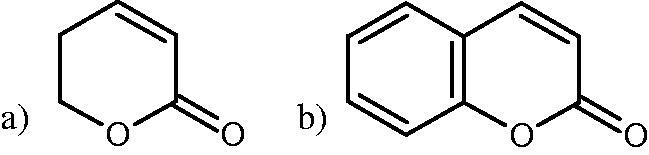
Structure of α-piron (a); benzo-α-piron (b).

Coumarins are derivatives of α-pyrone, condensed with benzene. Benzo-α-pyrone is usually substituted at C-7, less often at C-5, C-6, and C-8 positions with a hydroxyl group to which methyl groups or sugar moieties may be attached. A furan or pyran ring may be condensed with the benzo-α-pyrone structure (Figure 25).

Coumarins isolated from *Euphorbia lathyris* seeds showed weak inhibitory properties against tyrosinase. The exception was esculetin, whose IC_50_ was 43 µM (kojic acid; IC_50 _=10 µM) l^144^. Different results were obtained for the constituents present in the leaf extract from *M. alba* L. Scopoletin showed the strongest anti-tyrosinase properties (IC_50 _=0.2 µM), while esculetin and scopoline appeared to be less potent with the IC_50_ equal 6.9 and 15.9 µM, respectively. All inhibitors blocked the enzyme competently^145^.

Coumarin glycosides present in the *Morus nigra* roots showed weak inhibitory properties against tyrosinase, probably the presence of a sugar residue reduced the inhibitory activity^146^.

Some coumarins present in *Rhododendron collettianum* inhibited tyrosinase more strongly than kojic acid (IC_50 _=16.67 µM). 8′-Epi-cleomiscosin A (IC_50 _=1.33 µM) blocked the enzyme activity most strongly. Cleomiscosin A (IC_50 _=18.69 µM) differs from 8′-epi-cleomiscosin A by the position of the proton at position 8. Due to the change in stereochemistry of the single proton, the inhibitory activity of the compounds changes drastically. This may be due to stereochemically favourable binding conditions at the enzyme active site. Aquillochin (IC_50 _=15.69 µM) and 5,6,7-trimethoxycoumarin (IC_50 _=8.65 µM) also inhibited the enzyme more strongly than caffeic acid. This study also showed a negative impact of the sugar residue on an anti-tyrosinase activity (8-O-β-D-glucopyranosyl-6-hydroxy-2-methyl-4H-1-benzopyrane-4-one; IC_50 _=256.97 µM)^147^.

More information on the structure–activity relationship was provided by examining semi-synthetic/synthetic coumarin derivatives. In a study conducted by Matos et al., the effect of 3-phenylcoumarin and 3-thiophenylcoumarin derivatives on tyrosinase activity was investigated. L-DOPA was used as a substrate for the enzyme. The results showed that some synthesised derivatives exhibited an inhibitory activity against mashroom tyrosinase. The two most active compounds (5,7-dihydroxy-3–(3-thiophenyl)coumarin and 3–(4′-bromophenyl)-5,7-dihydroxycoumarin) showed tyrosinase inhibitory activity (IC_50_=0.19 and 1.05 µM, respectively), higher than kojic acid (IC_50_=17.90 µM). The presence of two hydroxyl groups at the C-5 and C-7 positions of the coumarin scaffold improved the inhibitory activity. The presence of the resorcinol grouping enhanced the ability to chelate copper ions^148^.

In subsequent studies, the effect of the position of hydroxyl, methoxyl, ethoxyl, and bromine groups in 3-phenylcoumarin derivatives on the activity against tyrosinase was examined. 3-Phenyl-6-hydroxy-8-bromocoumarin (IC_50 _=215 µM) inhibited tyrosinase more strongly than kojic acid (IC_50 _=420 µM). In addition, the introduction of more hydroxyl groups in the coumarin grouping increased the inhibitory activity. Compared to 6-hydroxy-8-bromocoumarin (IC_50 _=302 µM), one more hydroxyl group was introduced in the 3-phenyl-6-hydroxy-8-bromocoumarin, which improved the inhibitory activity about 1.5 times. A bromine substituent and a C-4′ hydroxyl group at the C-6 positions increase the inhibitory activity. In turn, methoxy and ethoxy derivatives weakly inhibited the enzyme. It seems that brominated hydroxycoumarin derivatives may be promising inhibitors of tyrosinase^149^.

In the study of Asthan et al., the effect of the position of the hydroxyl group in benzo-α-pyrone on tyrosinase activity was checked. The position of the hydroxyl group at C-6 and C-7 causes the molecule to behave as a weak substrate for the enzyme. This is related to the interaction of the hydroxyl group with the copper ion in the enzyme’s active centre, which means that the compounds with the OH group in the pyrone ring cannot be substrates for tyrosinase. Among investigated compounds, only 3-hydroxycoumarin inhibited the enzyme activity. The studies indicate the possibility of application of 3-hydroxycoumarin structure as a new class of tyrosinase inhibitors^150^.

In another study, thiosemicarbothioamide derivatives, such as 2–(1-(coumarin-3-yl)ethylidene)hydrazinecarbothioamide inhibited tyrosinase stronger than kojic acid with the IC_50_ values 3.44 and 23.0 µM, respectively. The compound blocked the enzyme irreversibly^151^.

**Figure 26.**
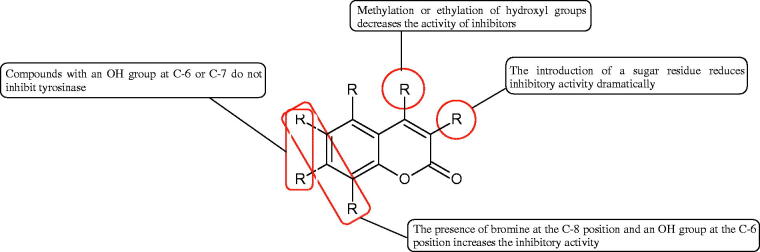
Potential groups engaged in an interaction coumarin-tyrosinase.

Ashraf et al. synthesised several umbelliferone derivatives and studied their effects on tyrosinase. Compounds 4e and 4c, having 2,4-dihydroxy and 3,4-dihydroxyphenyl group, inhibited fungal tyrosinase most potently with the IC_50_ values 8.96 and 118.48 µM, respectively. The other umbelliferone derivatives showed weak activity against mashroom tyrosinase compared to kojic acid (IC_50 _= 16.69 µM). On the other hand, umbelliferone derivatives obtained in this study inhibited tyrosinase more potently than umbelliferone (IC_50 _= 420 µM) (Figure 26; Table 22)^152^.

**Table 22.** Structure and activity of coumarin against tyrosinase.

Structure	Name	IC_50_ (μM)	Ref.
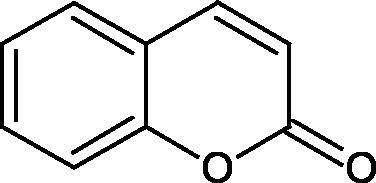	Coumarin	8100	Masamoto et al.^143^
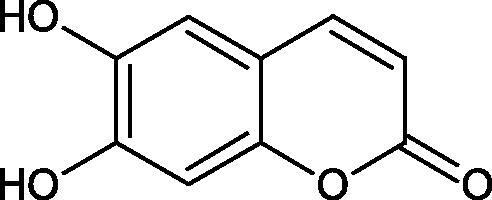	Esculetin	43/6.9	Masamoto et al.^143^; Li et al.^144^
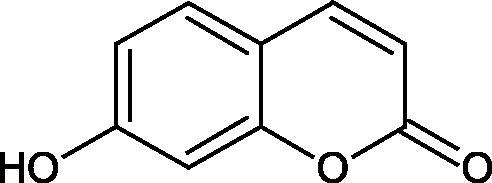	Umbelliferone	420	Masamoto et al.^143^
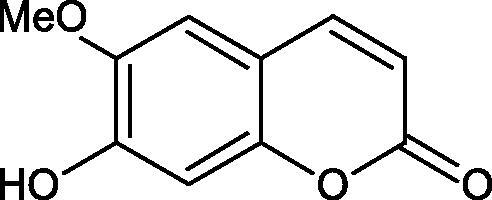	Scopoletin	2600/0.2	Masamoto et al.^143^; Li et al.^144^
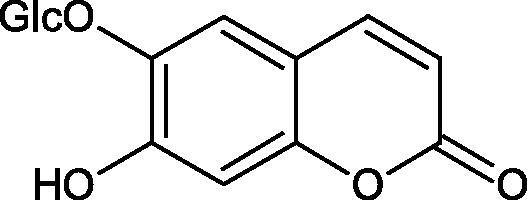	Esculin	>14000	Masamoto et al.^143^
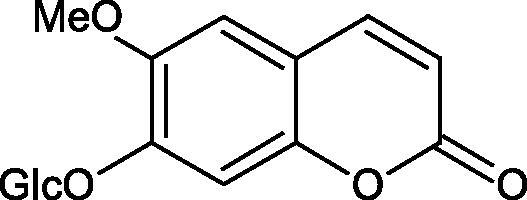	Scopoline	15.9	Li et al.^144^
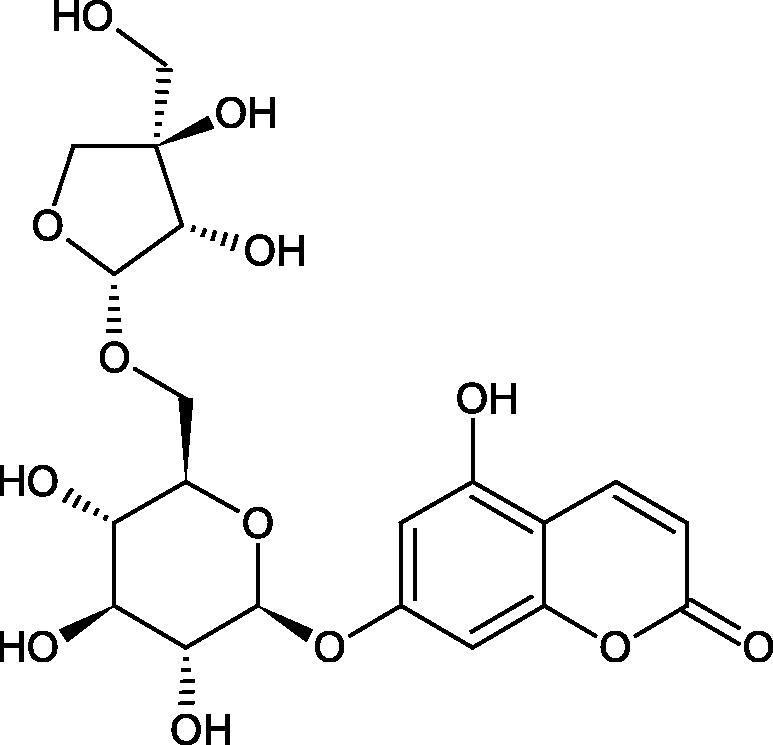	5,7-dihydroxycoumarin-7-(6-O-β-D-apiofuranosyl-β-Dglucopyranoside)	>400	Zheng et al.^145^
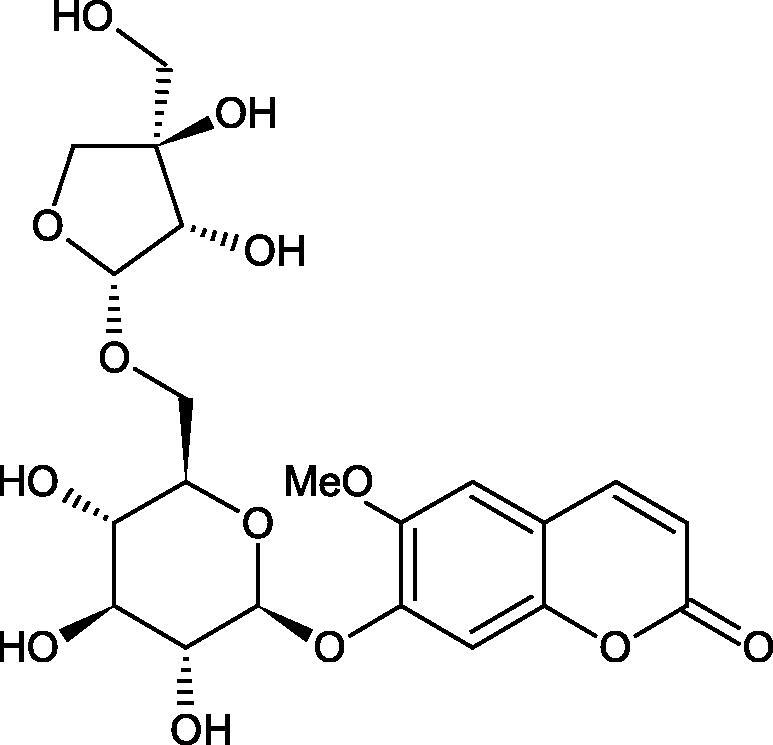	xeroboside	>400	Zheng et al.^145^
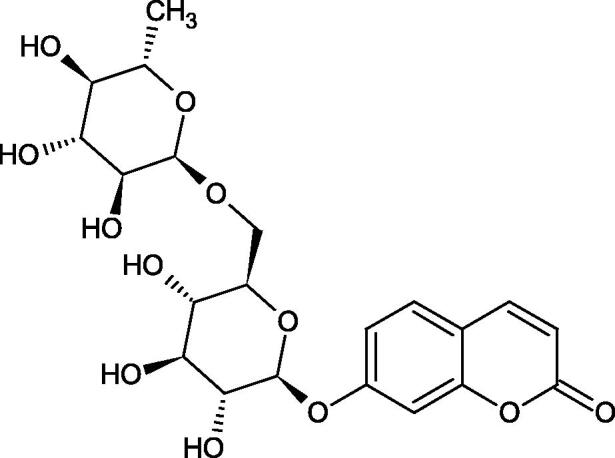	7-[[6-O-(6-deoxy-R-L-mannopyranosyl)-β-Dglucopyranosyl]oxy]-2H-1-benzopyran-2-one	>400	Zheng et al.^145^
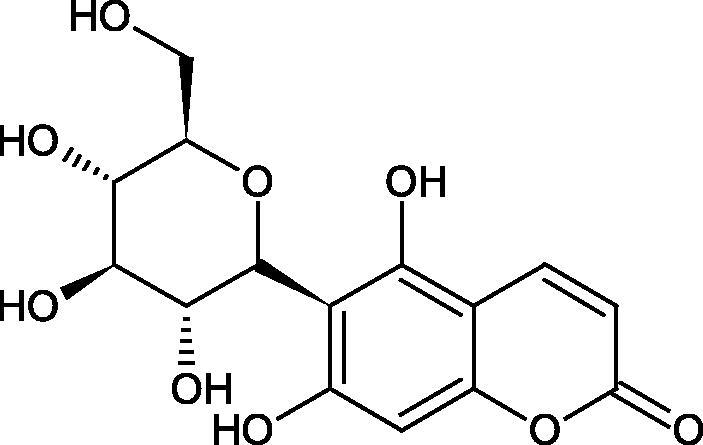	mulberroside B	>500	Zheng et al.^145^
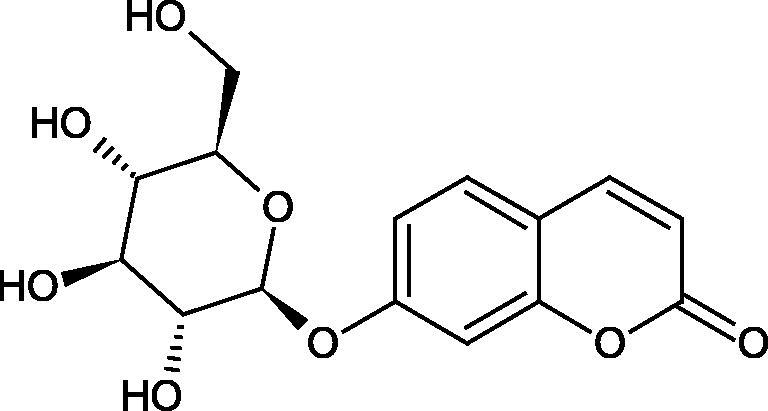	5,7-dihydroxycoumarin-7-O-β-D-glucopyranoside	>400	Zheng et al.^145^
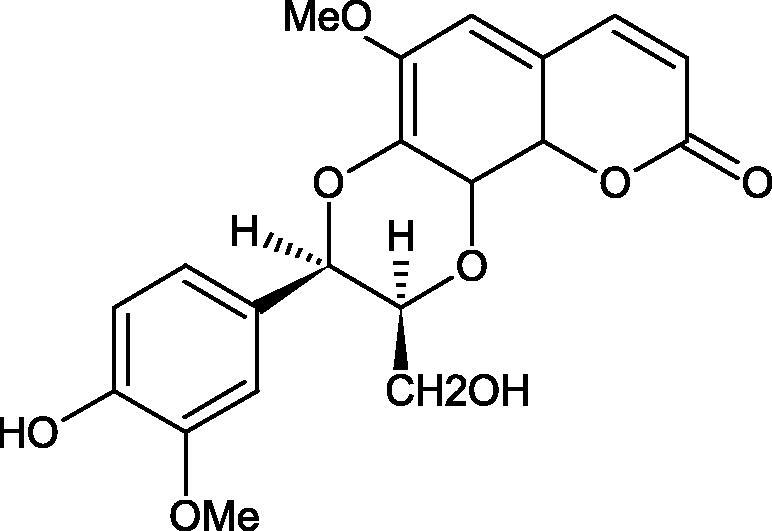	8′-epi-cleomiscosin A	1.33	Ahmad et al.^146^
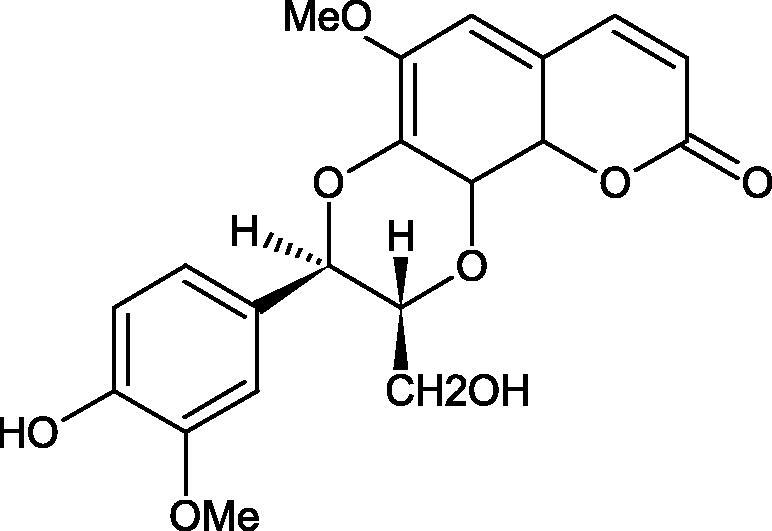	Cleomiscosin A	18.69	Ahmad et al.^146^
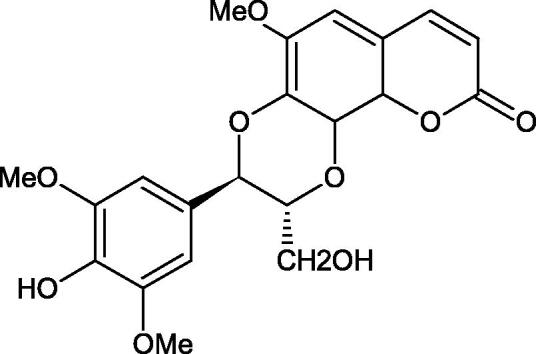	aquillochin	15.69	Ahmad et al.^146^
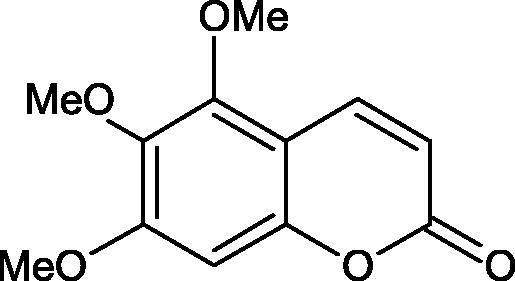	5,6,7-trimethoxycoumarin	8.65	Ahmad et al.^146^
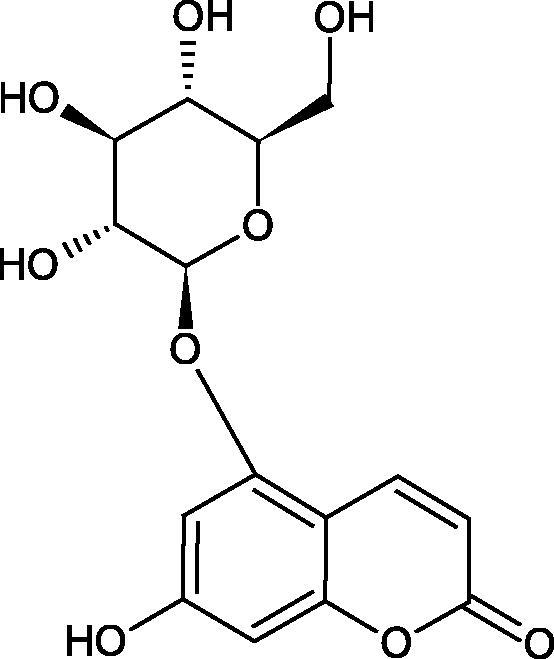	8-O-b-Dglucopyranosyl-6-hydroxy-2-methyl-4H-1-benzopyrane-4- one	256.97	Ahmad et al.^146^
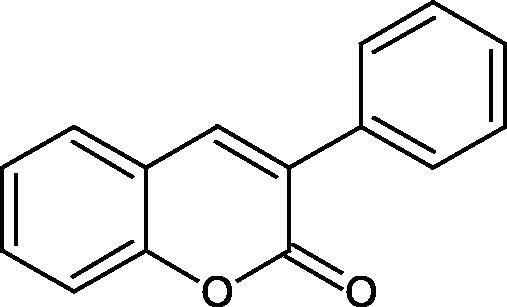	3-Phenylcoumarin	>1000	Matos et al.^147^
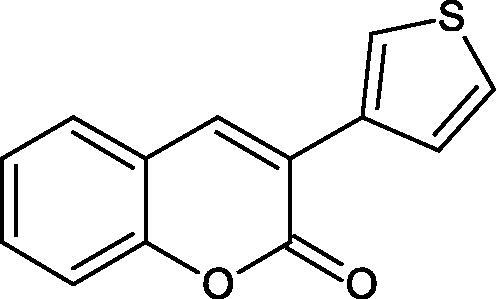	3-Thiophenylcoumarin	>1000	Matos et al.^147^
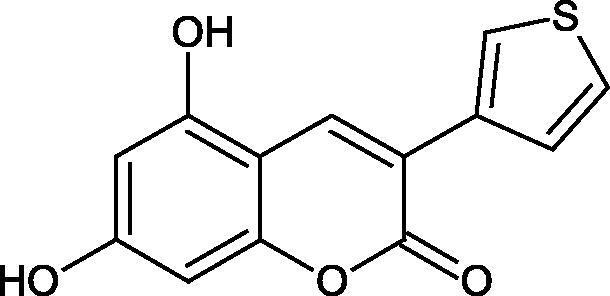	5,7-Dihydroxy-3-(3-thiophenyl)coumarin	0.19	Matos et al.^147^
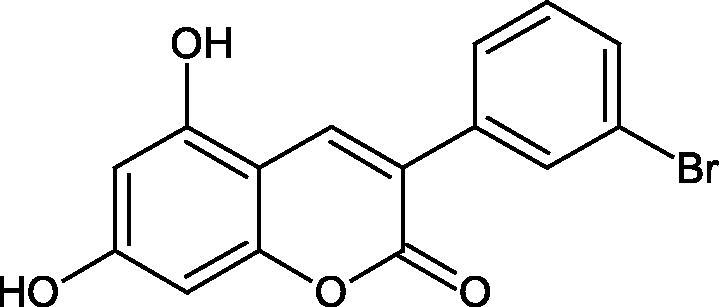	3-(4′-Bromophenyl)-5,7-dihydroxycoumarin	1.05	Matos et al.^147^
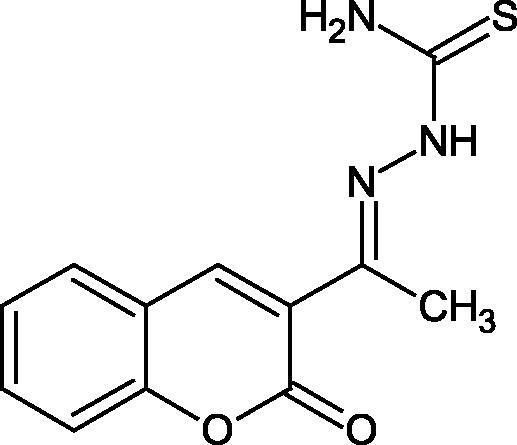	2-(1-(Coumarin-3-yl)ethylidene) hydrazinecarbothioamide	3.44	Liu et al.^150^
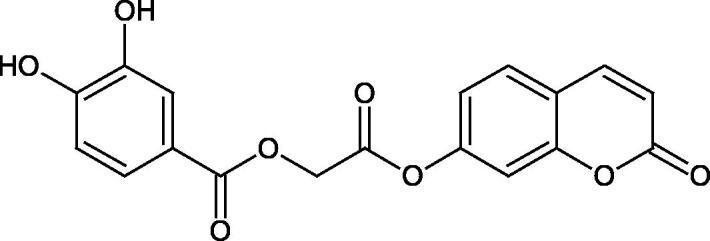	4c	118.48	Ashraf et al.^151^
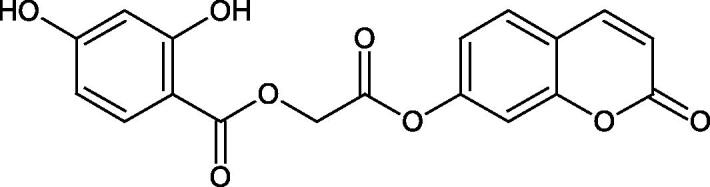	4e	8.96	Ashraf et al.^151^
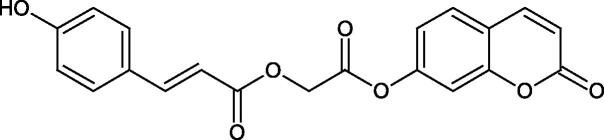	6a	123.77	Ashraf et al.^151^
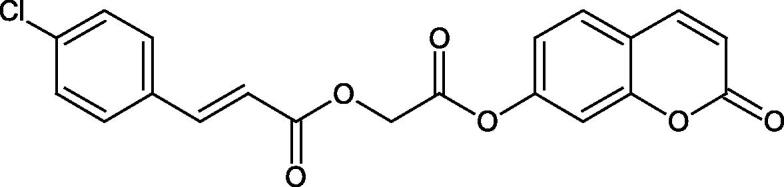	6b	80.20	Ashraf et al.^151^

### 5.4. Tannins

Another group of natural compounds with tyrosinase inhibitory potential is tannins. The flavan-3-ol derivatives isolated from *Potrntilla anserina* L. rhizome showed interesting inhibitory properties against tyrosinase. In both cases, the compounds inhibited diphenolases more strongly than monophenolases. Flavan-3-ol dimers (potenserin C – L-tyr IC_50 _= 16.53 µM, L-DOPA IC_50 _= 2.63 µM; gallocatechin-(4′→O → 7)-epigallocatechin-L-tyr IC_50 _= 18. 55 µM, L-DOPA IC_50 _= 6.62 µM; bis-6,8′-catechinylmethane – L-tyr IC_50 _= 30.56 µM, L-DOPA IC_50 _= 12.26 µM; bis-8,8′-catechinylmethane – L-tyr IC_50 _= 33. 89 µM, L-DOPA IC_50 _= 15.11 µM; catechin (4α → 8) catechin – L-tyr IC_50 _= 39.09 µM, L-DOPA IC_50 _= 25.49 µM; catechin (4α → 8) epicatechin – L-tyr IC_50 _= 37.22 µM, L-DOPA IC_50 _= 21.76 µM) showed similar or stronger inhibitory properties than kojic acid (L-tyr IC_50 _= 48.55 µM, L-DOPA IC_50 _= 21.00 µM). The presence of an additional OH group at the C-3′ position of catechins (L-tyr IC_50 _= 65.26 µM, L-DOPA IC_50 _= 42.71 µM) enhanced the anti-tyrosinase properties (gallocatechin – L-tyr IC_50 _= 41.96 µM, L-DOPA IC_50 _= 30.11 µM). In turn, methylation of the 3′-OH and 5′-OH groups in (2 R, 3S)- 3′, 5′-dimethoxy gallocatechin decreases the inhibitor activity^153^. A similar result was obtained by testing proanthocyanidin oligomers (OPC) from red wine (*Vitis vinifera*) for tyrosinase activity at a concentration of 1 mM. Procyanidin dimers as procyanidin B-3 (69.6% inhibition) and B-4 (69.4% inhibition), which possess (+)-catechin, more strongly blocked the enzyme than procyanidin B-1 (16.7% inhibition) and B-2 (26.0% inhibition). The IC_50_ values for PB3 and PB4 were 545 and 726 µM, respectively. The trimeric OPCs showed an inhibition rate of 25.3–31.1% at 1 mM, meaning no significant differences between the trimeric proanthocyanidins^154^.

The procyanidin epicatechin-(4β→8, 2β→O → 7)-epicatechin-(4β→8)-epicatechin isolated from *Guioa villosa* and procyanidin B1 have shown a minimal effect on tyrosinase activity^155^^,^^156^.

Another study examined the effect of tannins isolated from the methanolic extract of *Ecklonia stolonifera*. The extract contained five phlorotannins: phloroglucinol, eckstolonol, eckol, phlorofucofuroeckol A, and dieckol. Particularly noteworthy was dieckol (IC_50 _=2.16 µg/mL), which blocked the enzyme more strongly than kojic acid (IC_50 _=6.32 µg/mL) and arbutin (IC_50 _=112.0 µg/mL). Phloroglucinol and eckstolonol showed the competent type of inhibition. Eckol, phlorofucofuroeckol A, and dieckol inhibited the enzyme incompetently. The isolated phlorotannin derivatives owe their properties to the presence of a resorcinol group, which can chelate copper in the active tyrosinase center^157^.

Shoji et al. examined the effect of procyanidin polymerisation on tyrosinase activity. All oligomer fractions (1mer to 7mer) strongly inhibited tyrosinase activity, as did kojic acid. The IC_50_ values were similar for all groups: monomer, 74 µM; dimer, 235 µM; trimmer, 140 µM; tetramer, 149 µM; pentamer, 184 µM; hexamer, 127 µM; and heptamer 103 µM. No correlation was observed between the degree of procyanidin polymerisation and tyrosinase inhibition. Nevertheless, these observations suggest that procyanidins are effective inhibitors of tyrosinase (Table 23)^158^.

**Table 23.** Structure and activity of tannin against tyrosinase.

Structure	Name	IC_50_ (μM)	Ref.
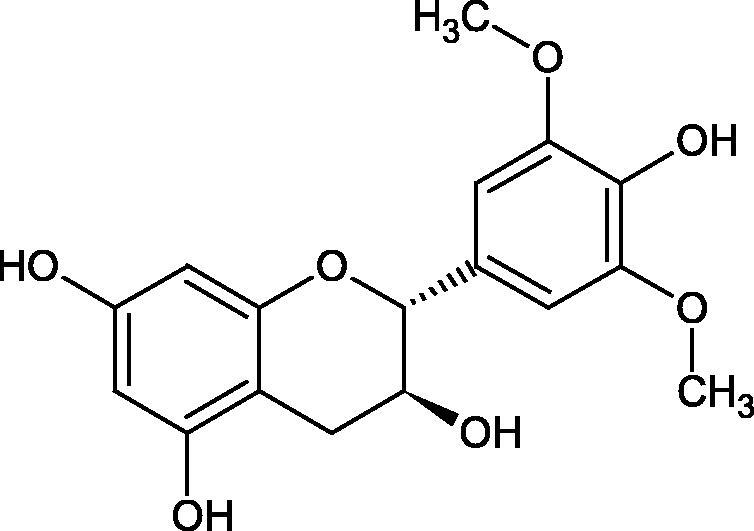	(2R, 3S)- 3′, 5′-Dimethoxy gallocatechin	47.33^a^, 35.79^b^	Yang et al.^152^
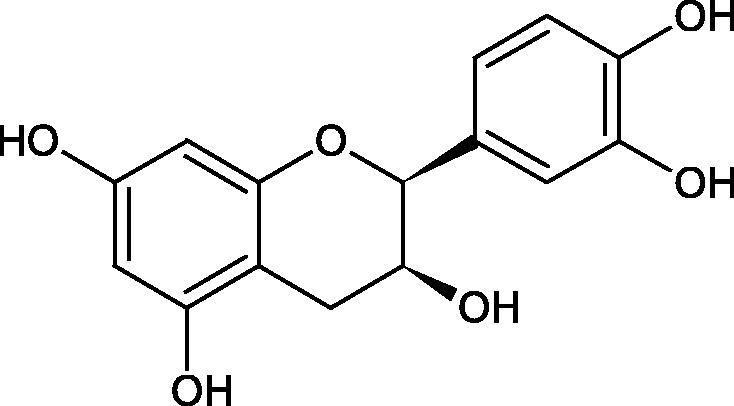	Epicatechin	73.88^a^, 48.98^b^	Yang et al.^152^
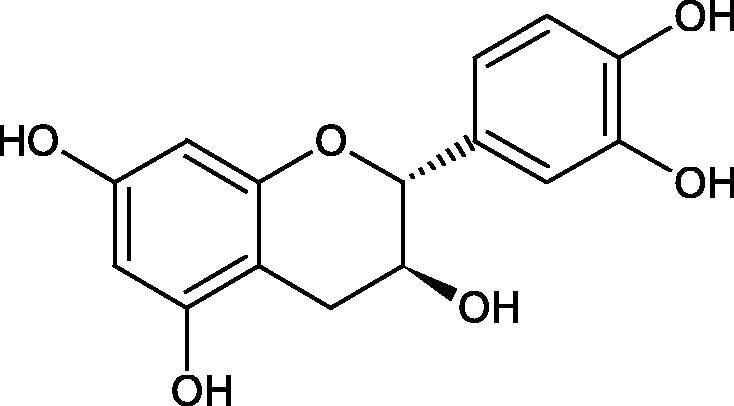	Catechin	65.26^a^, 42.71^b^	Yang et al.^152^
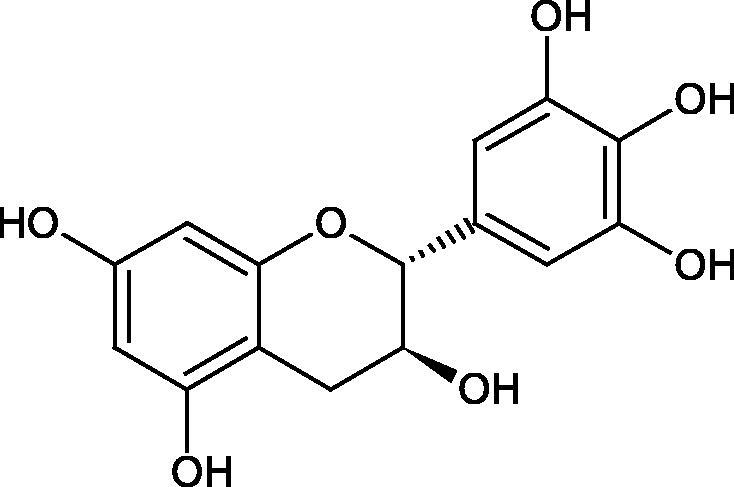	Gallocatechin	41.96^a^, 30.11^b^	Yang et al.^152^
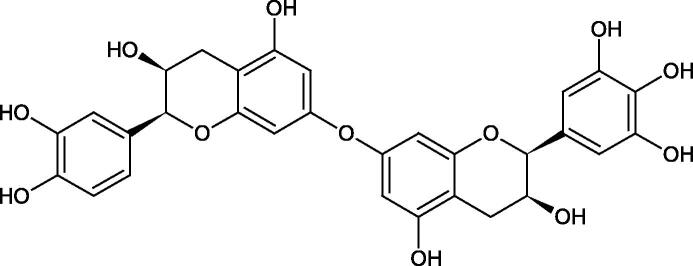	Potenserin C	16.53^a^, 2.63^b^	Yang et al.^152^
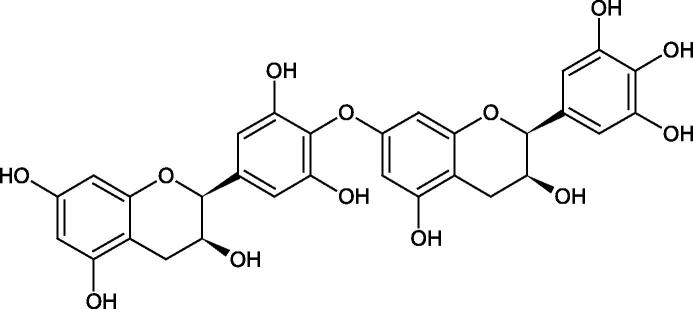	Gallocatechin-(*4′*→*O* → 7)-epigallocatechin	18.55^a^, 6.62^b^	Yang et al.^152^
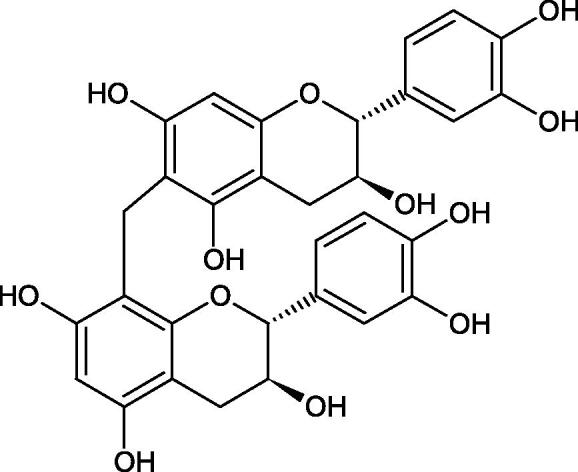	*Bis*-6,*8′*-catechinylmethane	30.56^a^, 12.26^b^	Yang et al.^152^
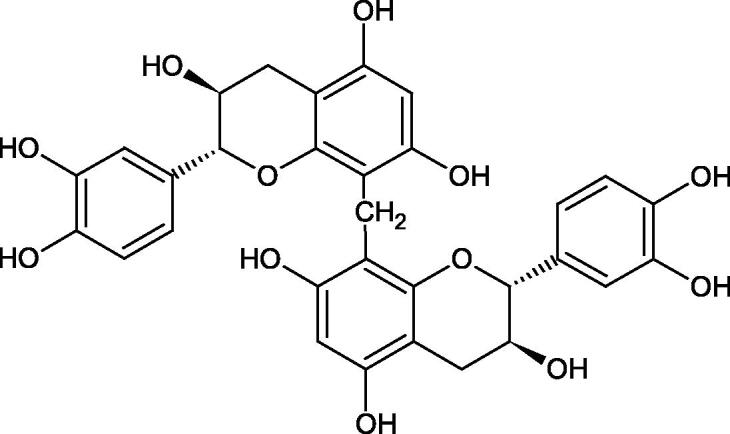	*Bis*-8,*8′*-catechinylmethane	33.88^a^, 15.11^b^	Yang et al.^152^
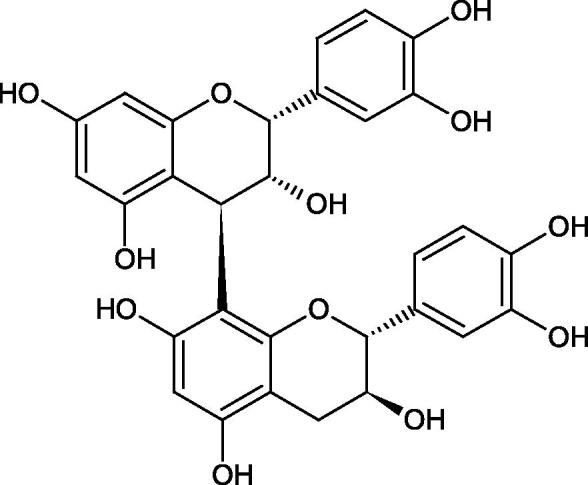	Epicatechin-(4β→8)-catechin - Procyanidin B-1	>346^a,b^	Momtaz et al.^154^
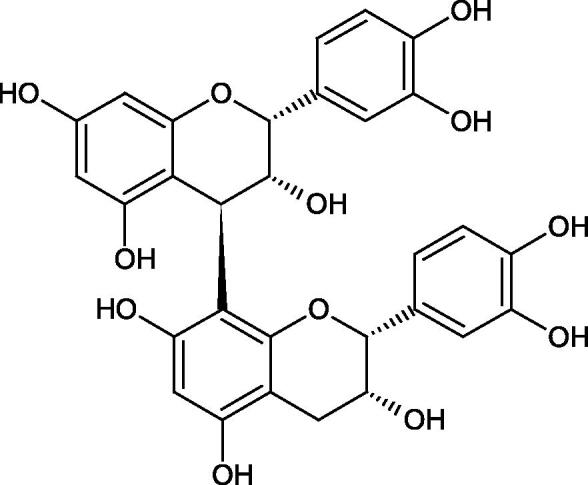	(−)-Epicatechin-(4β→8)- (−)-epicatechin Procyanidin B-2	–	Fujimaki et al.^153^
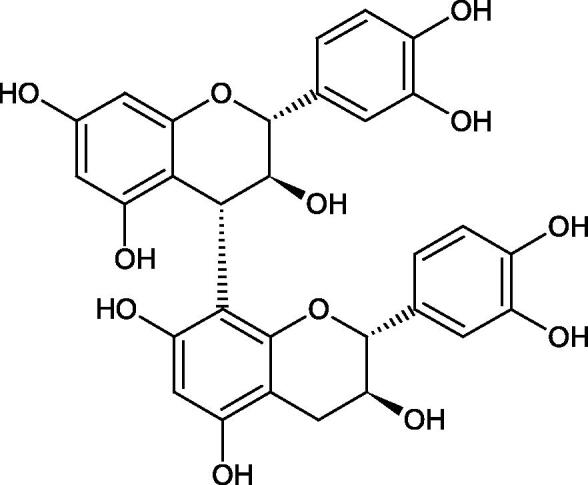	Catechin (4*α* → 8) catechin - procyanidin B-3	39.09^a^, 25.49^b^	Yang et al.^152^
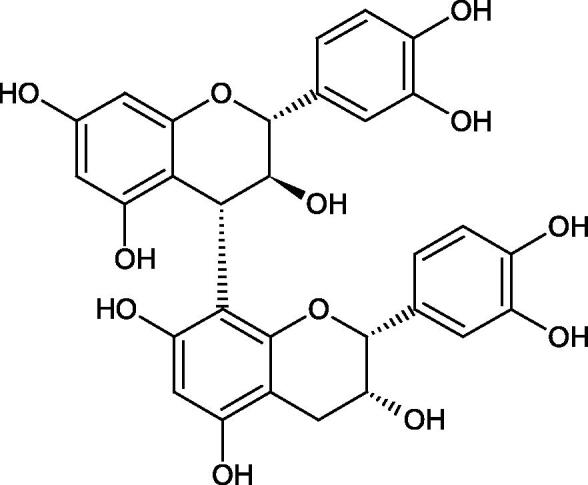	Catechin (4*α* → 8) epicatechin - procyanidin B-4	37.22^a^, 21.76^b^	Yang et al.^152^
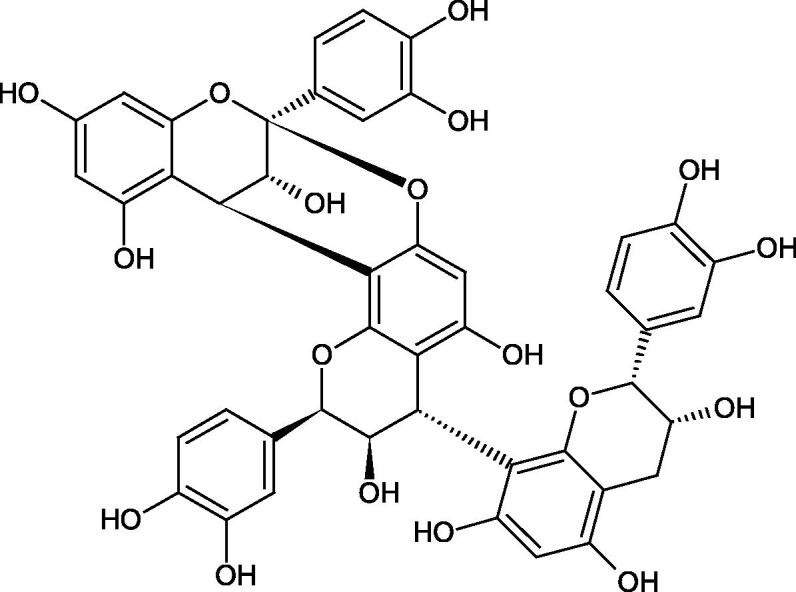	epicatechin-(4β→8, 2β→O→7)-epicatechin-(4β→8)-epicatechin	nd	Fujimaki et al.^153^
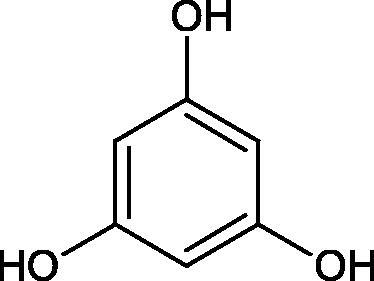	Phloroglucinol	92.8 μg/mL	Kang et al.^156^
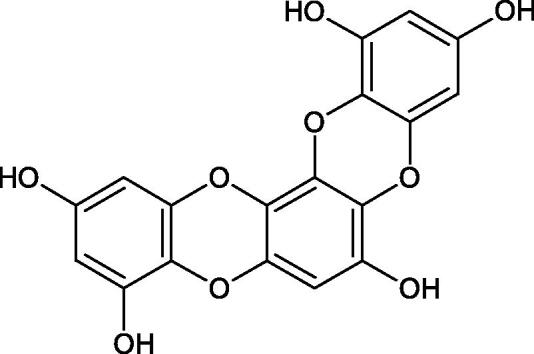	Eckstolonol	126.0 μg/mL	Kang et al.^156^
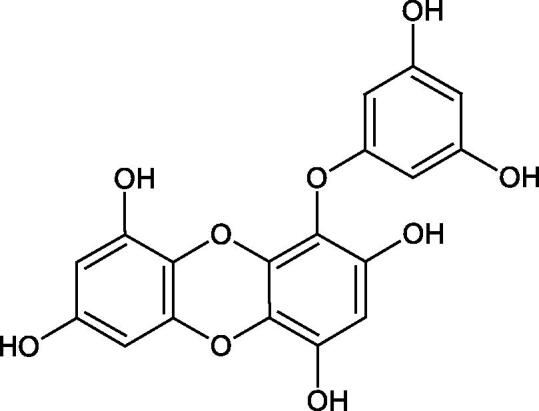	Eckol	33.2 μg/mL	Kang et al.^156^
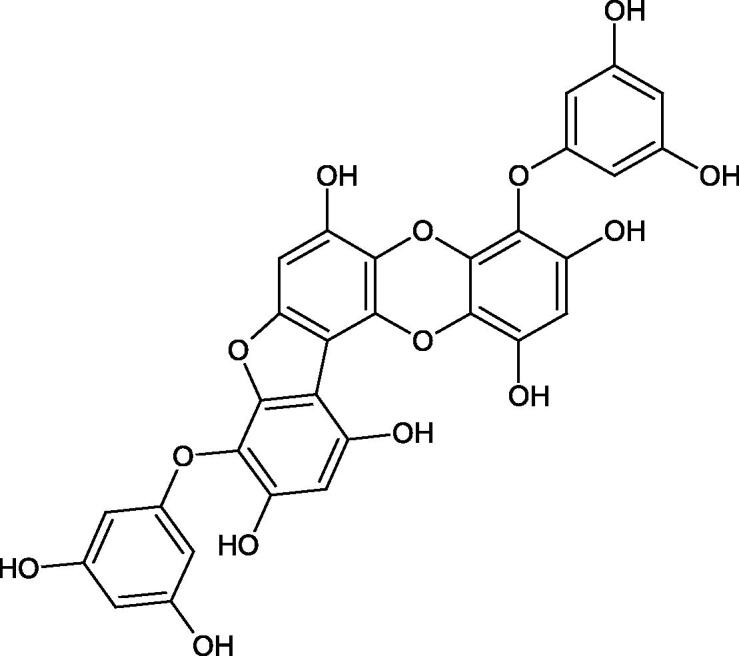	Phlorofucofuroeckol A	177.0 μg/mL	Kang et al.^156^
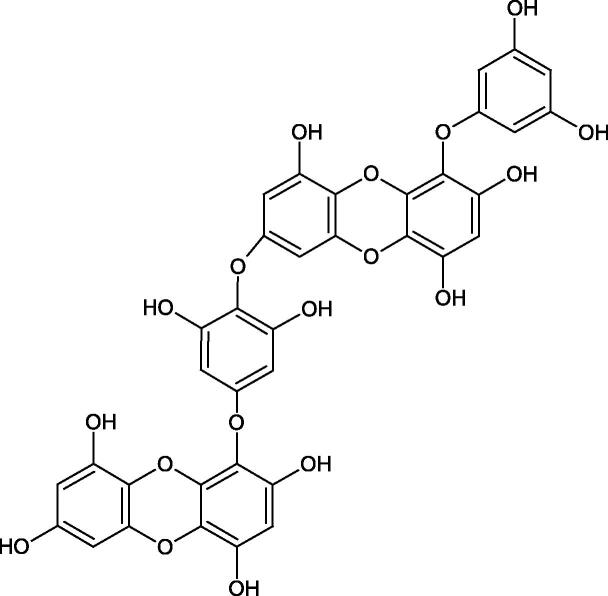	Dieckol	2.16 μg/mL	Kang et al.^156^

^a^L-tyrosine.^b^L-DOPA.

### 5.5. Terpenes

A very promising group of tyrosinase inhibitors are terpenes, for which that activity was proved by Lin et al. who examined the effect of terpenes isolated from the *Eucalyptus globulus* Labill leaves. Among few tested terpenes, the sesquiterpene, (-)-globulol (IC_50 _=9.79 µM) showed the strongest inhibition, followed by the isoiphionane sesquiterpene derivatives, 5-β-11-dihydroxy-iphionan-4-one (IC_50 _=10.08 µM) and 3-β-11-dihydroxyisoiphion-4-one (IC_50 _=14.17 µM). They have appeared to be more effective inhibitors than kojic acid (IC_50 _=17.32 µM)^159^. Isolated from the Aquilaria genus prezizane-type sesquiterpenes, agarosanol A–F weakly inhibited tyrosinase at a concentration of 100 µM (less than 30%) (Table 24)^160^.

**Table 24.** Structure and an anti-tyrosinase activity of terpenes.

Structure	Name	IC_50_ (μM)	Ref.
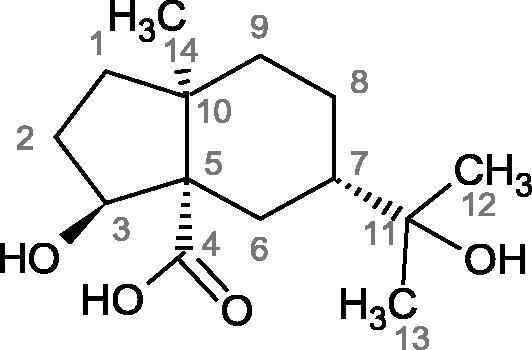	3-β-11-dihydroxyisoiphion-4-one	14.17	Lin et al.^158^
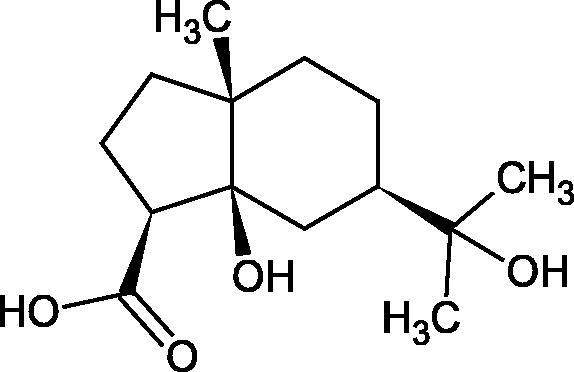	5-β-11-dihydroxy-iphionan-4-one	10.08	Lin et al.^158^
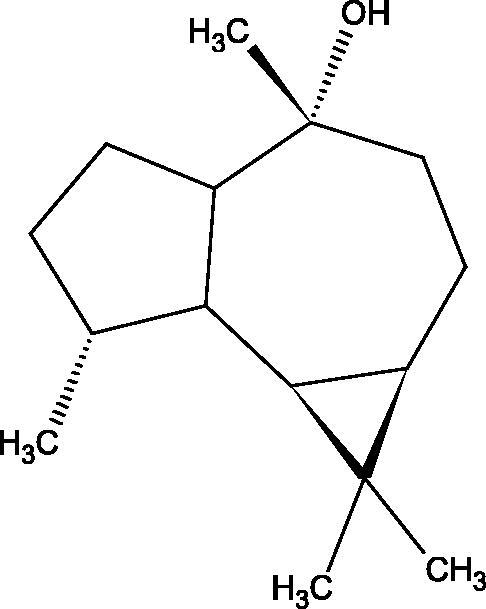	(-)-globulol	9.79	Lin et al.^158^
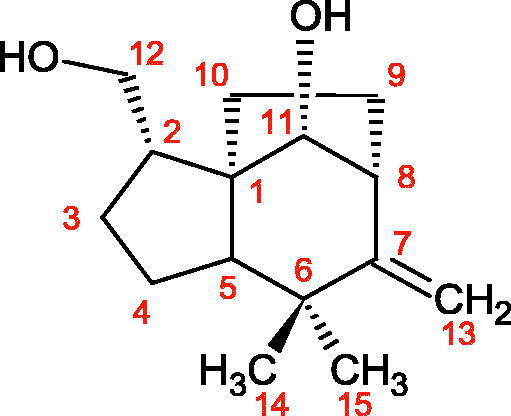	Agarozizanol A	–	Yang et al.^159^
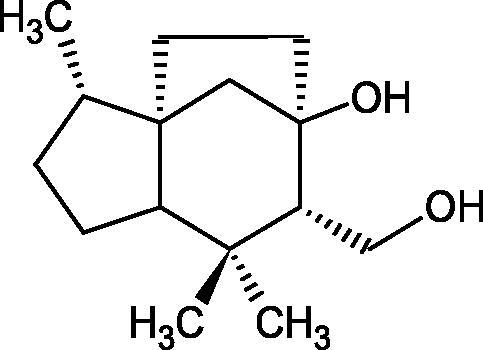	Agarozizanol B	–	Yang et al.^159^
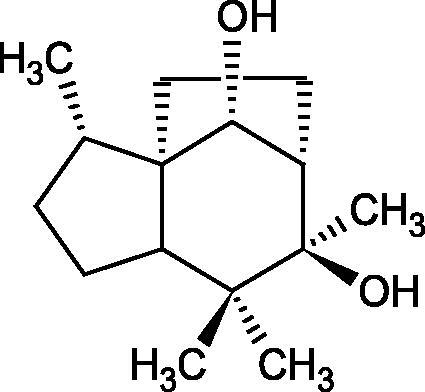	Agarozizanol C	–	Yang et al.^159^
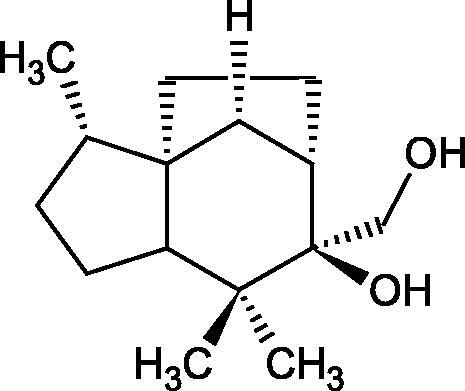	Agarozizanol D	–	Yang et al.^159^
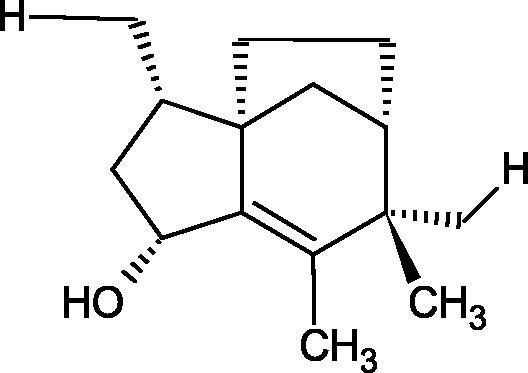	Agarozizanol E	–	Yang et al.^159^
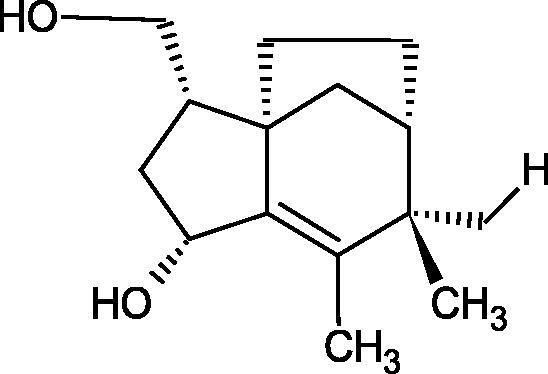	Agarozizanol F	–	Yang et al.^159^

## 6. Conclusion and future perspectives

Despite the fact that the plant-derived substances have always been a mainstay in medical treatment, the use of plants as a source of new drugs is still poorly studied. Of the 250,000–500,000 plant species, only a small number have been adequately studied. Besides the possibility of a direct use of plant materials in medicine, isolated substances can be used as a source of the structures for the synthesis of new drugs.

Commercially available Hyal and tyrosinase inhibitors are characterised by a range of adverse properties. Tyrosinase overactivity can be reduced by inhibitors such as hydroquinone, arbutin, vitamin C, azelaic acid, kojic acid, and ellagic acid. However, those inhibitors have many side effects, e.g. hydroquinone, the most commonly used inhibitor, shows mutagenic and irritating effects (dermatitis and irritation), while arbutin, a prodrug of hydroquinone, is a chemically unstable compound. Kojic acid is carcinogenic, L-AA is quickly degraded, ellagic acid, due to its poor solubility, shows a low bioavailability. Therefore, it is necessary to search for new safe inhibitors^161–165^.

One of the Hyal inhibitors available for treatment is escin (aescin). Unfortunately, due to its physicochemical properties, the compound is absorbed orally only to a small extent^166^.

From this review, it is clear that currently used tyrosinase and Hyal inhibitors are not without their drawbacks, therefore, there is a great need to search for the new inhibitors with more valuable pharmacological properties. In the further bioprospecting, the following criteria should be considered:

1. Examine the effect of extracts and newly isolated compounds on cell cultures to determine cytotoxicity. Some compounds, e.g. alkaloids or terpenes, may exhibit toxic effects. Therefore, early determination of their toxicity is necessary. In addition, the study of new inhibitors using cell cultures will allow us to initially determine the metabolic pathways and mode of absorption (active or passive transport) of the tested compounds.
2. To better understand the utility of new substances in inhibiting Hyal or tyrosinase, more studies using animal models should be planned to determine the safety and efficacy of the tested inhibitors.
3. Most of the work was done with tyrosinase isolated from the fungus *Agaricus bisporus*. Human tyrosinase is membrane-bound, while fungal tyrosinase is a cytosolic enzyme. Furthermore, the human enzyme is a monomer that undergoes an intensive glycation process, unlike the fungal tyrosinase, which is a tetramer. Knowledge about the effects of tyrosinase inhibitors on the human enzyme is limited, thus, it is necessary to determine the suitability of fungal tyrosinase inhibitors relative to the human enzyme.
4. The search for selective Hyal inhibitors is also essential. The availability of selective inhibitors that target one type of Hyal without affecting another Hyal is important because different isoforms of Hyal regulate physiological processes. Additionally, the search for selective Hyal inhibitors will allow for a better understanding of the role of Hyals in both physiological and pathological processes.
5. A significant problem when comparing the results of studies is the deficiencies in analysing enzymatic reaction kinetic. Information on Km, Vmax, and Cmax of the enzymatic reaction is often missing in studies.
6. Many studies, especially on Hyal, focus only on determining the properties of anti-Hyal extracts. A more careful analysis of the active ingredients is needed.
